# Supplementary material for: Psilocybin-assisted group psychotherapy and mindfulness-based stress reduction for frontline healthcare provider COVID-19-related depression and burnout: A randomized controlled trial
Source: PLoS Med. 2025 Sep 19;22(9):e1004519. doi: 10.1371/journal.pmed.1004519 (PMC12459851; doi:10.1371/journal.pmed.1004519)
Supplement: S1 Appendix — Supplement 1. Additional tables presenting primary and secondary outcomes, sensitivity analyses, and exploratory findings. Table A1. ITT analyses for QIDS-SR-16 and MBI-HSS-MP. Table A2. Adjusted ITT analyses for QIDS-SR-16 and MBI-HSS-MP. Table B1. Intent-To-Treat (ITT) Analysis for Demoralization Scale (DSII) and PTSD Checklist for DSM-5 (PCL-5). Table B2. Adjusted Intent-To-Treat (ITT) Analysis for DSII PCL-5. Table C1. Intent-To-Treat (ITT) Analysis for Watt’s Connectedness Scale (WCS-GC). Table C2. Adjusted Intent-To-Treat (ITT) Analysis for Watt’s Connectedness Scale (WCS-GC). Table D1. Intent-To-Treat (ITT) Analysis for Watt’s Connectedness Scale Subscales. Table D2. Adjusted Intent-To-Treat (ITT) Analysis for Watt’s Connectedness Scale Subscales. Table E. p-values for Time × Study Arm interaction across all time points. Table F. Simple Effects for significant interaction results (ITT Analysis). Table G. Preference and Expectancy Measures. Table H. Bivariate Fit of Change in Outcome Measures by Experiential Questionnaires. Table I. Bivariate Fit of Change in Outcome Measures by Experiential Questionnaires by study arm. Table J. Correlations between outcome measures from baseline to 2-week endpoint. Table K. FDR-adjusted p-values for secondary outcome measures. Table L. Baseline-Adjusted Mixed Model Results for QIDS-SR-16. Table M. Baseline-Adjusted Mixed Model Results for MBI(EE). Table N. Baseline-Adjusted Mixed Model Results for MBI (DP). Table O. Baseline-Adjusted Mixed Model Results for MBI (PA). Table P. Baseline-Adjusted Mixed Model Results for WCS(GC). Table Q. EM Covariances (Little’s MCAR test). Table R. Per-Protocol Analysis for QIDS-SR-16 and MBI-HSS-MP. Table S. Simple effect for significant interaction results, per-protocol analysis. Supplement 2. Group psilocybin protocol outlining preparatory sessions, dosing protocol, and integration sessions. Text A. Intervention Structure. Text B. Guided Meditations and Guided Imagery. Text C. Citations. (DO [file pmed.1004519.s001.docx]

**Supplementary Appendix for Psilocybin-Assisted Group Psychotherapy and Mindfulness Based Stress Reduction for Frontline Healthcare Provider COVID-19 Related Depression and Burnout: A Randomized Clinical Trial**

**Contents**

**Supplement 1: Intention-to-Treat Analyses and Per-Protocol Analyses**

1. **Table A1: ITT analyses for QIDS-SR-16 and MBI-HSS-MP………………………………………………………....………2**
2. **Table A2: Adjusted ITT analyses for QIDS-SR-16 and MBI-HSS-MP………………………………………………………2**
3. **Table B1: Intent-To-Treat (ITT) Analysis for Demoralization Scale (DSII) and PTSD Checklist for DSM-5 (PCL-5)..….3**
4. **Table B2: Adjusted Intent-To-Treat (ITT) Analysis for DSII and PCL-5……………………………………….……………3**
5. **Table C1: Intent-To-Treat (ITT) Analysis for Watt’s Connectedness Scale (WCS-GC) ……………………………….…....4**
6. **Table C2: Adjusted Intent-To-Treat (ITT) Analysis for Watt’s Connectedness Scale (WCS-GC) …………………….…...4**
7. **Table D1: Intent-To-Treat (ITT) Analysis for Watt’s Connectedness Scale Subscales………………………………....……4**
8. **Table D2: Adjusted Intent-To-Treat (ITT) Analysis for Watt’s Connectedness Scale Subscales……………………....……5**
9. **Table E: p-values for Time x Study Arm interaction across all time points……………………………………………….....5**
10. **Table F: Simple Effects for significant interaction results (ITT Analysis)…………………………………………………...5-6**
11. **Table G: Preference and Expectancy Measures………………………………………………………………………………..7**
12. **Table H: Bivariate Fit of Change in Outcome Measures by Experiential Questionnaires…………………………………..7**
13. **Table I: Bivariate Fit of Change in Outcome Measures by Experiential Questionnaires by study arm……………..…..…7**
14. **Table J: Correlations between outcome measures from baseline to 2-week endpoint……………………………..….…..…7**
15. **Table K: FDR-adjusted p-values for secondary outcome measures…………………………………. ………………...…….8**
16. **Table L: Baseline-Adjusted Mixed Model Results for QIDS-SR-16………………………………………………..………....8**
17. **Table M: Baseline-Adjusted Mixed Model Results for MBI(EE)………………………………………………….…………..8**
18. **Table N: Baseline-Adjusted Mixed Model Results for MBI(DP)………………………………………………….………..…8**
19. **Table O: Baseline-Adjusted Mixed Model Results for MBI(PA)……………………………………………………….…..…8**
20. **Table P: Baseline-Adjusted Mixed Model Results for WCS(GC)……………………………………………………..……....9**
21. **Table Q: EM Covariances (Little’s MCAR test)………………………………………………………………………..…..…..9**
22. **Table R: Per-Protocol Analysis for QIDS-SR-16 and MBI-HSS-MP…………………………………………….…………...10**
23. **Table S: Simple effect for significant interaction results, per-protocol analysis………………………………..………….....10**

**Supplement 2: Group Psilocybin Intervention**

1. **Text A: Intervention Structure………………………………………………………………………………………………..….11-13**
2. **Text B: Guided Meditations and Guided Imagery……………………………………………………………………………...13-15**
3. **Text C: Citations……………………………………………………………………………………………………………/…….16**

**Supplement 1: Intention-to-Treat (ITT) Analyses and Per-Protocol Analyses.**

|  | QIDS | | | MBI_EE | | | MBI_DP | | | MBI_PA | | |
| --- | --- | --- | --- | --- | --- | --- | --- | --- | --- | --- | --- | --- |
| *Predictors* | *Estimates* | *CI* | *p* | *Estimates* | *CI* | *p* | *Estimates* | *CI* | *p* | *Estimates* | *CI* | *p* |
| (Intercept) | 12.08 | 10.20,13.95 | **<0.001** | 42.15 | 36.98, 47.33 | **<0.001** | 17.85 | 14.25,  21.44 | **<0.001** | 28.38 | 24.22, 32.55 | **<0.001** |
| Time [2.Week] | -7.26 | -9.35 ,-5.17 | **<0.001** | -15.94 | -21.83, -10.05 | **<0.001** | -7.66 | -11.14, -4.18 | **<0.001** | 8.08 | 3.91, 12.24 | **<0.001** |
| Time [6 Month] | -5.93 | -8.01, -3.84 | **<0.001** | -20.05 | -26.11, -13.99 | **<0.001** | -9.28 | -12.86, -5.69 | **<0.001** | 7.77 | 3.47, 12.06 | **0.001** |
| Group | 0.38 | -2.39 , 3.15 | 0.786 | 0.66 | -6.98,8.31 | 0.863 | -1.85 | -7.16, 3.47 | 0.490 | 3.34 | -2.81, 9.49 | 0.281 |
| Time [2.Week] × Group | 4.60 | 1.51,7.70 | **0.004** | 7.83 | -0.89, 16.56 | 0.078 | 5.47 | 0.31, 10.63 | **0.038** | -4.58 | -10.75, 1.59 | 0.143 |
| Time [3.Month 6] × Group [2.MBSR + PAP] | 1.37 | -1.72, 4.46 | 0.379 | 10.95 | 2.10, 19.79 | **0.016** | 4.88 | -0.35, 10.11 | 0.067 | -3.57 | -9.82,   2.69 | 0.259 |
| Random Effects | | | | | | | | | | | | |
| σ^2^ | 6.69 | | | 53.26 | | | 18.46 | | | 26.47 | | |
| τ_00_ | 4.76 _participant_ | | | 33.72 _participant_ | | | 23.57 _participant_ | | | 29.80 _participant_ | | |
| ICC | 0.42 | | | 0.39 | | | 0.56 | | | 0.53 | | |
| N | 25 _participant_ | | | 25 _participant_ | | | 25 _participant_ | | | 25 _participant_ | | |
| Observations | 68 | | | 67 | | | 67 | | | 67 | | |
| Marginal R^2^ / Conditional R^2^ | 0.430 / 0.667 | | | 0.411 / 0.639 | | | 0.211 / 0.653 | | | 0.147 / 0.599 | | |

**Table A1: Intent-To-Treat (ITT) Analysis for Quick Inventory of Depressive Symptomatology (QIDS-SR-16) and Maslach Burnout Inventory – Human Services Survey for Medical Personnel (MBI-HSS-MP). Subscales: EE = emotional exhaustion; DP = depersonalization; and PA = personal accomplishment.** Mixed model analysis with group x time interactions at 2 week and 6 month endpoints. Participant as random effect. Significant effects **(p < .05**) are bolded.

|  | | | | | | | | | | **MBI_PA** | | |
| --- | --- | --- | --- | --- | --- | --- | --- | --- | --- | --- | --- | --- |
|  | **QIDS** | | | **MBI_EE** | | | **MBI_DP** | | |  |  |  |
| *Predictors* | *Estimates* | *CI* | *p* | *Estimates* | *CI* | *p* | *Estimates* | *CI* | *p* | *Estimates* | *CI* | *p* |
| (Intercept) | 11.38 | 8.65, 14.11 | **<0.001** | 28.09 | 21.56, 34.62 | **<0.001** | 24.16 | 19.79, 28.53 | **<0.001** | 28.09 | 21.56, 34.62 | **<0.001** |
| Time [2.Week 2] | -7.11 | -9.21, -5.01 | **<0.001** | 7.88 | 3.72, 12.05 | **<0.001** | -7.65 | -11.09, -4.20 | **<0.001** | 7.88 | 3.72, 12.05 | **<0.001** |
| Time [3.Month 6] | -5.78 | -7.87, -3.68 | **<0.001** | 7.39 | 3.09, 11.69 | **0.001** | -9.24 | -12.80, -5.68 | **<0.001** | 7.39 | 3.09, 11.69 | **0.001** |
| Group [2.MBSR] | 2.44 | -1.78, 6.65 | 0.252 | -0.04 | -10.10, 10.03 | 0.994 | -8.11 | -14.85, -1.37 | **0.019** | -0.04 | -10.10, 10.03 | 0.994 |
| Gender Male [1] | -0.65 | -3.23, 1.93 | 0.614 | 5.95 | -0.42, 12.31 | 0.067 | -6.79 | -10.88, -2.70 | **0.002** | 5.95 | -0.42, 12.31 | 0.067 |
| Cohort [1B] | -2.21 | -6.01, 1.60 | 0.250 | 0.92 | -8.38, 10.23 | 0.843 | 5.40 | -0.65, 11.45 | 0.079 | 0.92 | -8.38, 10.23 | 0.843 |
| Cohort [2A] | 3.62 | 0.11, 7.13 | **0.043** | -4.97 | -13.67, 3.73 | 0.257 | -5.24 | -10.91, 0.42 | 0.069 | -4.97 | -13.67, 3.73 | 0.257 |
| Cohort [2B] | -0.54 | -4.35, 3.26 | 0.776 | 0.26 | -9.05, 9.56 | 0.956 | 4.65 | -1.40, 10.70 | 0.129 | 0.26 | -9.05, 9.56 | 0.956 |
| Cohort [3A] | -0.95 | -4.18, 2.29 | 0.560 | 3.55 | -4.45, 11.56 | 0.377 | -10.86 | -16.00, -5.73 | **<0.001** | 3.55 | -4.45, 11.56 | 0.377 |
| Time [2.Week 2] × Group [2.MBSR] | 4.52 | 1.41, 7.63 | **0.005** | -4.28 | -10.46, 1.89 | 0.170 | 5.16 | 0.06, 10.27 | **0.048** | -4.28 | -10.46, 1.89 | 0.170 |
| Time [3.Month 6] × Group [2.MBSR] | 1.29 | -1.82, 4.40 | 0.409 | -3.09 | -9.36, 3.18 | 0.327 | 4.56 | -0.63, 9.74 | 0.084 | -3.09 | -9.36, 3.18 | 0.327 |
| **Random Effects** | | | | | | | | | |  |  |  |
| σ^2^ | 6.72 | | | 53.49 | | | 18.16 | | | 26.33 | 26.33 | 26.33 |
| τ_00_ | 3.32 _participant_ | | | 26.41 _participant_ | | | 7.93 _participant_ | | | 25.23 _participant_ | 25.23 _participant_ | 25.23 _participant_ |
| ICC | 0.33 | | | 0.33 | | | 0.30 | | | 0.49 | 0.49 | 0.49 |
| N | 25 _participant_ | | | 25 _participant_ | | | 25 _participant_ | | | 25 _participant_ | 25 _participant_ | 25 _participant_ |
| Observations | 68 | | | 67 | | | 67 | | | 67 | 67 | 67 |
| Marginal R^2^ / Conditional R^2^ | 0.530 / 0.686 | | | 0.495 / 0.662 | | | 0.532 / 0.674 | | | 0.299 / 0.642 | 0.299 / 0.642 | 0.299 / 0.642 |

**Table A2: Adjusted Intent-To-Treat (ITT) Analysis for Quick Inventory of Depressive Symptomatology (QIDS-SR-16) and Maslach Burnout Inventory – Human Services Survey for Medical Personnel (MBI-HSS-MP), adjusted for gender and group cohort. Subscales: EE = emotional exhaustion; DP = depersonalization; and PA = personal accomplishment.** Mixed model analysis with group x time interactions at 2 week and 6 month endpoints. Participant as random effect. Gender and group cohort as fixed effects. Significant effects **(p < .05**) are bolded.

| *Predictors* | *Estimate* | *95% CI* | *p* |
| --- | --- | --- | --- |
| **DSII (Demoralization Scale)** |  |  |  |
| (Intercept) | 14.38 | 11.09, 17.68 | **<0.001** |
| Time [2 weeks] | -10.60 | -14.15, -7.05 | **<0.001** |
| Group | 2.25 | -2.62, 7.12 | 0.355 |
| Time [2 weeks] × Group | 5.89 | 0.63, 11.15 | **0.029** |
| σ² | 18.92 |  | |
| τ₀₀ (participant) | 15.65 |  |  |
| ICC | 0.45 |  |  |
| N | 25 _participant_ |  |  |
| Observations | 46 |  |  |
| Marginal R² / Conditional R² | 0.417 / 0.681 |  |  |
| **PCL-5 (PTSD Checklist for DSM-5)** |  |  |  |
| (Intercept) | 32.00 | 25.52, 38.48 | **<0.001** |
| Time [2 weeks] | –24.25 | –31.75, –16.75 | **<0.001** |
| Group [MBSR + PAP] | 6.30 | –3.31, 15.91 | 0.195 |
| Time [2 weeks] × Group [MBSR+PAP] | 9.95 | –1.17, 21.07 | 0.079 |
| Time [6 months] | –22.42 | –29.92, –14.92 | <0.001 |
| Time [6 months] × Group [MBSR+PAP] | 6.12 | –5.01, 17.24 | 0.276 |
| τ₀₀ (participant) | 41.61 |  |  |
| ICC | 0.33 |  |  |
| N  Observations | 25 _participant_  66 |  |  |
| Observations | 66 |  |  |
| Marginal R² / Conditional R² | 0.500 / 0.665 |  |  |

**Table B1: Intent-To-Treat (ITT) Analysis for Demoralization Scale (DSII) and PTSD Checklist for DSM-5 (PCL-5).** Mixed model analysis with group x time interactions at 2 week and 6 month endpoints. Participant as random effect. Significant effects **(p < .05**) are bolded.

|  | **DSII** | | | **PCL_5** | | |
| --- | --- | --- | --- | --- | --- | --- |
| *Predictors* | *Estimates* | *CI* | *p* | *Estimates* | *CI* | *p* |
| (Intercept) | 15.77 | 10.64, 20.90 | **<0.001** | 29.32 | 20.95, 37.70 | **<0.001** |
| Time [2.Week 2] | -10.44 | -13.97, -6.90 | **<0.001** | -24.25 | -31.76, -16.74 | **<0.001** |
| Time [3.Month 6] |  |  |  | -22.42 | -29.93, -14.90 | **<0.001** |
| Group [2.MBSR] | 0.02 | -7.89, 7.93 | 0.996 | 11.41 | -2.30, 25.13 | 0.101 |
| Gender Male [1] | -0.22 | -5.27, 4.84 | 0.932 | 3.92 | -3.74, 11.58 | 0.309 |
| Cohort [1B] | -0.93 | -8.29, 6.44 | 0.800 | -10.42 | -22.38, 1.54 | 0.086 |
| Cohort [2A] | 2.61 | -4.22, 9.44 | 0.442 | 12.34 | 1.62, 23.06 | **0.025** |
| Cohort [2B] | 3.57 | -3.79, 10.94 | 0.331 | -0.58 | -12.54, 11.38 | 0.922 |
| Cohort [3A] | -5.65 | -12.01, 0.70 | 0.080 | -1.77 | -11.23, 7.69 | 0.709 |
| Time [2.Week 2] × Group [2.MBSR] | 5.67 | 0.42, 10.92 | **0.035** | 9.95 | -1.20, 21.10 | 0.079 |
| Time [3.Month 6] × Group [2.MBSR] |  |  |  | 6.12 | -5.03, 17.26 | 0.276 |
| σ^2^ | 18.62 | | | 84.21 | | |
| τ_00_ | 11.62 _participant_ | | | 19.35 _participant_ | | |
| ICC | 0.38 | | | 0.19 | | |
| N | 25 _participant_ | | | 25 _participant_ | | |
| Observations | 46 | | | 66 | | |
| Marginal R^2^ / Conditional R^2^ | 0.531 / 0.711 | | | 0.603 / 0.677 | | |

**Table B2: Adjusted Intent-To-Treat (ITT) Analysis for Demoralization Scale (DSII) and PTSD Checklist for DSM-5 (PCL-5). Adjusted for gender and group cohort.** Mixed model analysis with group x time interactions at 2 week and 6 month endpoints. Participant as random effect. Gender and group cohort as fixed effects. Significant effects **(p < .05**) are bolded.

|  | WCS_(General_Connectedness) | | |
| --- | --- | --- | --- |
| *Predictors* | *Estimates* | *CI* | *p* |
| (Intercept) | 48.01 | 39.83, 56.18 | **<0.001** |
| Time [2 Week] | 28.06 | 19.93, 36.19 | **<0.001** |
| Group | -1.15 | -13.23, 10.92 | 0.849 |
| Time [2 Week] × Group | -17.51 | -29.56, -5.47 | **0.005** |
| Time [6 month] | 24.92 | 16.79, 33.05 | **<0.001** |
| Time [6 month] × Group | -10.28 | -22.33, 1.77 | 0.093 |
| σ^2^ | 100.92 | | |
| τ_00_ | 116.31 _participant_ | | |
| ICC | 0.54 | | |
| N | 25 _participant_ | | |
| Observations | 68 | | |
| Marginal R^2^ / Conditional R^2^ | 0.381 / 0.712 | | |

**Table C1: Intent-To-Treat (ITT) Analysis for Watt’s Connectedness Scale (WCS) – General Connectedness.**

Mixed model analysis with group x time interactions at 2 week and 6 month endpoints. Participant as random effect. Significant effects (**p < .05**) are bolded.

|  | **WCS (General_Connectedness)** | | |
| --- | --- | --- | --- |
| *Predictors* | *Estimates* | *CI* | *p* |
| (Intercept) | 45.40 | 32.21, 58.60 | **<0.001** |
| Time [2.Week 2] | 28.01 | 19.87, 36.15 | **<0.001** |
| Group [2.MBSR] | -0.60 | -20.96, 19.77 | 0.954 |
| Gender Male [1] | 9.05 | -3.91, 22.01 | 0.167 |
| Cohort [1B] | 4.45 | -14.46, 23.36 | 0.639 |
| Cohort [2A] | 1.17 | -16.36, 18.69 | 0.894 |
| Cohort [2B] | -12.41 | -31.31, 6.50 | 0.194 |
| Cohort [3A] | 4.03 | -12.27, 20.32 | 0.622 |
| Time [2.Week 2] Ã— Group [2.MBSR] | -17.15 | -29.22, -5.08 | **0.006** |
| Time [3.Month 6] | 24.87 | 16.74, 33.01 | **<0.001** |
| Time [3.Month 6] Ã— Group [2.MBSR] | -9.92 | -21.99, 2.15 | 0.105 |
| σ^2^ | 100.57 | | |
| τ_00_ | 107.57 _participant_ | | |
| ICC | 0.52 | | |
| N | 25 _participant_ | | |
| Observations | 68 | | |
| Marginal R^2^ / Conditional R^2^ | 0.456 / 0.737 | | |

**Table C2: Adjusted Intent-To-Treat (ITT) Analysis for Watt’s Connectedness Scale (WCS) – General Connectedness. Adjusted for gender and group cohort.** Mixed model analysis with group x time interactions at 2 week and 6 month endpoints. Participant as random effect. Gender and group cohort as fixed effects. Significant effects (**p < .05**) are bolded.

|  | | | | | | | | | |
| --- | --- | --- | --- | --- | --- | --- | --- | --- | --- |
|  | **CTS** | | | **CTO** | | | **CTW** | | |
| *Predictors* | *Estimates* | *CI* | *p* | *Estimates* | *CI* | *p* | *Estimates* | *CI* | *p* |
| (Intercept) | 55.12 | 46.40, 63.84 | **<0.001** | 40.91 | 32.77, 49.04 | **<0.001** | 48.05 | 34.66, 61.45 | **<0.001** |
| Time [2.Week 2] | 24.18 | 15.27, 33.10 | **<0.001** | 37.76 | 27.91, 47.61 | **<0.001** | 22.38 | 10.64, 34.11 | **<0.001** |
| Time [3.Month 6] | 20.91 | 11.99, 29.82 | **<0.001** | 34.02 | 24.17, 43.87 | **<0.001** | 19.97 | 8.23, 31.70 | **0.001** |
| Group | -0.39 | -13.62, 12.83 | 0.953 | -3.91 | -16.25, 8.43 | 0.529 | -3.16 | -23.47, 17.15 | 0.756 |
| Time [2.Week] × Group | -18.79 | -32.09, -5.50 | **0.006** | -18.39 | -33.11, -3.67 | **0.015** | -13.69 | -31.16, 3.79 | 0.122 |
| Time [6 Month] × Group | -8.00 | -21.29, 5.29 | 0.233 | -15.89 | -30.61, -1.17 | **0.035** | -5.22 | -22.69, 12.26 | 0.552 |
| Random Effects | | | | | | | | | |
| σ^2^ | 121.40 | | | 149.38 | | | 209.29 | | |
| τ_00_ | 125.51 _participant_ | | | 65.55 _participant_ | | | 372.95 _participant_ | | |
| ICC | 0.51 | | | 0.30 | | | 0.64 | | |
| N | 25 _participant_ | | | 25 _participant_ | | | 25 _participant_ | | |
| Observations | 67 | | | 67 | | | 67 | | |
| Marginal R^2^ / Conditional R^2^ | 0.285 / 0.649 | | | 0.543 / 0.683 | | | 0.140 / 0.691 | | |

**Table D1: Intent-To-Treat (ITT) Analysis for Watt’s Connectedness Scale Subscales:** CTS = Connection to Self; CTO = Connection to Others; CTW = Connection to World. Mixed model analysis with group x time interactions at 2 week and 6 month endpoints. Participant as random effect. Significant effects (**p < .05**) are bolded.

|  | | | | | | | | | |
| --- | --- | --- | --- | --- | --- | --- | --- | --- | --- |
|  | **CTS** | | | **CTO** | | | **CTW** | | |
| *Predictors* | *Estimates* | *CI* | *p* | *Estimates* | *CI* | *p* | *Estimates* | *CI* | *p* |
| (Intercept) | 55.79 | 40.77, 70.81 | **<0.001** | 35.78 | 26.04, 45.51 | **<0.001** | 45.11 | 20.80, 69.42 | **<0.001** |
| Time [2.Week 2] | 24.25 | 15.29, 33.20 | **<0.001** | 37.52 | 27.73, 47.31 | **<0.001** | 22.39 | 10.60, 34.17 | **<0.001** |
| Time [3.Month 6] | 20.97 | 12.02, 29.93 | **<0.001** | 33.78 | 23.99, 43.57 | **<0.001** | 19.98 | 8.19, 31.77 | **0.001** |
| Group [2.MBSR] | -4.60 | -29.66, 20.46 | 0.715 | 0.46 | -15.41, 16.33 | 0.954 | -4.04 | -44.77, 36.69 | 0.843 |
| Gender Male [1] | 9.02 | -6.07, 24.10 | 0.236 | 7.13 | -1.37, 15.63 | 0.098 | 8.99 | -15.96, 33.95 | 0.473 |
| Cohort [1B] | 3.45 | -20.10, 27.00 | 0.770 | 6.22 | -7.05, 19.48 | 0.352 | 10.06 | -28.90, 49.01 | 0.607 |
| Cohort [2A] | 0.67 | -19.34, 20.67 | 0.947 | -1.71 | -13.25, 9.83 | 0.767 | 4.40 | -28.49, 37.28 | 0.790 |
| Cohort [2B] | -5.88 | -29.43, 17.68 | 0.619 | -13.22 | -26.48, 0.05 | 0.051 | -11.76 | -50.71, 27.20 | 0.548 |
| Cohort [3A] | -4.07 | -22.70, 14.56 | 0.663 | 13.28 | 2.79, 23.78 | **0.014** | 2.33 | -28.48, 33.14 | 0.880 |
| Time [2.Week 2] × Group [2.MBSR] | -18.86 | -32.20, -5.52 | **0.006** | -18.15 | -32.81, -3.49 | **0.016** | -13.70 | -31.23, 3.84 | 0.123 |
| Time [3.Month 6] × Group [2.MBSR] | -8.06 | -21.40, 5.27 | 0.231 | -15.65 | -30.31, -0.99 | **0.037** | -5.23 | -22.76, 12.30 | 0.552 |
| **Random Effects** | | | | | | | | | |
| σ^2^ | 121.54 | | | 148.07 | | | 209.59 | | |
| τ_00_ | 143.49 _participant_ | | | 9.02 _participant_ | | | 433.50 _participant_ | | |
| ICC | 0.54 | | | 0.06 | | | 0.67 | | |
| N | 25 _participant_ | | | 25 _participant_ | | | 25 _participant_ | | |
| Observations | 67 | | | 67 | | | 67 | | |
| Marginal R^2^ / Conditional R^2^ | 0.316 / 0.687 | | | 0.676 / 0.694 | | | 0.189 / 0.736 | | |

**Table D2: Adjusted Intent-To-Treat (ITT) Analysis for Watt’s Connectedness Scale.** Adjusted for gender and group cohort. Subscales**:** CTS = Connection to Self; CTO = Connection to Others; CTW = Connection to World. Mixed model analysis with group x time interactions at 2 week and 6 month endpoints. Participant as random effect. Gender and group cohort as fixed effects. Significant effects (**p < .05**) are bolded.

| ***Outcome*** | ***p value*** | ***Adjusted p value**** |
| --- | --- | --- |
| QIDS-SR-16 | **0.02** | **0.02** |
| MBI_EE | **0.05** | **0.05** |
| MBI_DP | 0.08 | 0.09 |
| MBI_PA | 0.31 | 0.33 |
| DSII | **0.04** | **0.04** |
| WCS (General Connectedness) | **0.02** | **0.02** |
| CTS | **0.03** | **0.03** |
| CTO | **0.03** | **0.03** |
| CTW | 0.30 | 0.30 |
| PCL-5 | 0.21 | 0.21 |

**Table E: p values for Time x Study Arm interaction across all time points.** Quick Inventory of Depressive Symptomatology (QIDS-SR-16); Maslach Burnout Inventory – Human Services Survey for Medical Personnel (MBI-HSS-MP). Mixed model analysis with participant as random effect. Adjusted p values (*) indicate controlling for gender and cohort (as fixed effects). Significant effects (**p < .05**) are bolded.

Subscales: EE = emotional exhaustion; DP = depersonalization; and PA = personal accomplishment; Demoralization Scale (DSII) (only with 2 time points at Baseline and 2-week endpoint); Watt’s Connectedness Scale (WCS) Subscales: CTS = Connection to Self; CTO = Connection to Others; CTW = Connection to World.

| ***Outcome*** | ***Group*** | ***Contrast*** | ***Estimate*** | ***SE*** | ***p.value*** |
| --- | --- | --- | --- | --- | --- |
| QIDS | MBSR only | Baseline- 2 Week | 7.26 | 1.04 | 0.00 |
| QIDS | MBSR only | Baseline – 6 Month | 5.93 | 1.04 | 0.00 |
| QIDS | MBSR only | 2 Week – 6 Month | -1.33 | 1.06 | 0.42 |
| QIDS | MBSR + PAP | Baseline- 2 Week | 2.66 | 1.14 | 0.06 |
| QIDS | MBSR + PAP | Baseline – 6 Month | 4.56 | 1.14 | 0.00 |
| QIDS | MBSR + PAP | 2 Week – 6 Month | 1.90 | 1.16 | 0.24 |
| MBI_EE | MBSR only | Baseline- 2 Week | 15.94 | 2.95 | 0.00 |
| MBI_EE | MBSR only | Baseline – 6 Month | 20.05 | 3.04 | 0.00 |
| MBI_EE | MBSR only | 2 Week – 6 Month | 4.11 | 3.07 | 0.38 |
| MBI_EE | MBSR + PAP | Baseline- 2 Week | 8.10 | 3.22 | 0.04 |
| MBI_EE | MBSR + PAP | Baseline – 6 Month | 9.10 | 3.22 | 0.02 |
| MBI_EE | MBSR + PAP | 2 Week – 6 Month | 1.00 | 3.26 | 0.95 |
| DSII | MBSR only | Baseline- 2 Week | 10.60 | 1.76 | 0.00 |
| DSII | MBSR + PAP | 2 Week – 6 Month | 4.71 | 1.93 | 0.02 |
| WCS_general_connectedness | MBSR only | Baseline- 2 Week | -28.06 | 4.07 | 0.00 |
| WCS_general_connectedness | MBSR only | Baseline – 6 Month | -24.92 | 4.07 | 0.00 |
| WCS_general_connectedness | MBSR only | 2 Week – 6 Month | 3.14 | 4.10 | 0.73 |
| WCS_general_connectedness | MBSR + PAP | Baseline- 2 Week | -10.55 | 4.45 | 0.06 |
| WCS_general_connectedness | MBSR + PAP | Baseline – 6 Month | -14.64 | 4.45 | 0.01 |
| WCS_general_connectedness | MBSR + PAP | 2 Week – 6 Month | -4.10 | 4.49 | 0.64 |
| CTS | MBSR only | Baseline- 2 Week | -24.18 | 4.46 | 0.00 |
| CTS | MBSR only | Baseline – 6 Month | -20.91 | 4.46 | 0.00 |
| CTS | MBSR only | 2 Week – 6 Month | 3.28 | 4.50 | 0.75 |
| CTS | MBSR + PAP | Baseline- 2 Week | -5.39 | 4.93 | 0.52 |
| CTS | MBSR + PAP | Baseline – 6 Month | -12.91 | 4.93 | 0.03 |
| CTS | MBSR + PAP | 2 Week – 6 Month | -7.52 | 4.93 | 0.29 |
| CTO | MBSR only | Baseline- 2 Week | -37.76 | 4.93 | 0.00 |
| CTO | MBSR only | Baseline – 6 Month | -34.02 | 4.93 | 0.00 |
| CTO | MBSR only | 2 Week – 6 Month | 3.74 | 4.99 | 0.74 |
| CTO | MBSR + PAP | Baseline- 2 Week | -19.37 | 5.47 | 0.00 |
| CTO | MBSR + PAP | Baseline – 6 Month | -18.13 | 5.47 | 0.01 |
| CTO | MBSR + PAP | 2 Week – 6 Month | 1.24 | 5.47 | 0.97 |
| CTW | MBSR only | Baseline- 2 Week | -22.38 | 5.87 | 0.00 |
| CTW | MBSR only | Baseline – 6 Month | -19.97 | 5.87 | 0.00 |
| CTW | MBSR only | 2 Week – 6 Month | 2.41 | 5.91 | 0.91 |
| CTW | MBSR + PAP | Baseline- 2 Week | -8.69 | 6.47 | 0.38 |
| CTW | MBSR + PAP | Baseline – 6 Month | -14.75 | 6.47 | 0.07 |
| CTW | MBSR + PAP | 2 Week – 6 Month | -6.06 | 6.47 | 0.62 |

**Table F. Simple Effects for significant interaction results (ITT Analysis)**

| ****Preference and Expectancy Measures**** | ****MBSR+PAP (N=13)**** | ****MBSR-only (N=12)**** | ****p-value (between groups)**** |
| --- | --- | --- | --- |
| ****Treatment Preference, Mean (SD)**** | 23.9 (9.8) | 22.2 (24.3) | 0.426 |
| ****Pre-randomization Expectancy, Mean (SD)**** |  |  |  |
| ****MBSR+PAP**** | 78.3 (13.2) | 83.3 (13.6) | 0.805 |
| ****MBSR-only**** | 72.2 (14.1) | 70.1 (11.1) | *p*=0.349 |
| ****Post-randomization Expectancy, Mean (SD)**** | 65.4 (14.7) | 37.6 (17.9) | **0.0003** |
|  |  |  |  |
| ****Correlation with Change in QIDS (Baseline to 2-week)**** (95% CI) | 0.039 (−0.55, 0.50)  p=0.903 | −0.71 (−0.93, −0.14)  **p=0.022** |  |

**Table G: Preference and Expectancy Measures.** *p-*values represent 2-tailed t-test between 2 study arms. Correlation p-values reflect significance for each study arm. Significant effects (**p < .05**) are bolded.

| ***MEQ-30*** | | ***NADA-state*** |  | |
| --- | --- | --- | --- | --- |
| ***Outcome Measures*** | ***Correlation (95% CI)*** | ***p-value*** | ***Correlation (95% CI)*** | ***p-value*** |
| **QIDS-SR-16** | -0.624 (-0.828, -0.275) | **0.0019** | -0.654 (-0.851, -0.298) | **0.0018** |
| **MBI (EE)** | -0.467 (-0.742, -0.056) | **0.0286** | -0.423 (-0.729, 0.024) | 0.0632 |
| **MBI (DP)** | -0.437 (-0.725, -0.019) | **0.0421** | -0.432 (-0.734, 0.013) | 0.0573 |
| **MBI (PA)** | 0.360 (-0.187, 0.736) | 0.1875 | 0.428 (-0.108, 0.771) | 0.1119 |
| **DS-II** | -0.355 (-0.675, 0.079) | 0.1052 | -0.401 (-0.717, 0.050) | 0.0793 |
| **WCS** | 0.616 (0.263, 0.824) | **0.0023** | 0.719 (0.405, 0.881) | **0.0004** |
| **PCL-5** | -0.564 (-0.796, -0.187) | **0.0063** | -0.646 (-0.847, -0.285) | **0.0021** |

**Table H: Bivariate Fit of Change in Outcome Measures from Baseline to 2-week Endpoint by Mystical Experience Questionnaire (MEQ-30) and by the Nondual Awareness Dimensional Assessment-State Questionnaire (NADA-state).** Analysis of entire study sample with no significant between group differences. No significant correlations found with the Challenging Experience Questionnaire (CEQ). Significant effects (**p < .05**) are bolded.

| ***Outcome Measures*** | ***MEQ-30*** | | | | ***NADA-state*** | | |  |
| --- | --- | --- | --- | --- | --- | --- | --- | --- |
|  | ***Correlation (95% CI)*** | | | | ***Correlation (95% CI)*** | | | |
|  | ***MBSR+PAP*** | ***p-value*** | ***MBSR only*** | ***p-value*** | ***MBSR+PAP*** | ***p-value*** | ***MBSR only*** | ***p-value*** |
| **QIDS-SR-16** | -0.377 (-0.782, 0.251) | 0.2273 | -0.697 (-0.922, -0.119) | **0.0251** | -0.421 (-0.831, 0.284) | 0.2255 | -0.582 (-0.887, 0.076) | 0.0777 |
| **MBI (EE)** | -0.339 (-0.764, 0.291 | 0.2807 | -0.239 (-0.755, 0.459) | 0.5043 | -0.469 (-0.848, 0.228) | 0.1720 | -0.138 (0-.706, 0.538) | 0.7029 |
| **MBI (DP)** | -0.079 (-0.625, 0.517) | 0.8056 | -0.285 (-0.776, 0.419) | 0.4240 | -0.061 (-0.665, 0.591) | 0.8662 | -0.395 (-0.821, 0.312) | 0.2588 |
| **MBI (PA)** | 0.286 (-0.457, 0.799) | 0.4552 | 0.661 (-0.325, 0.958) | 0.1532 | 0.377 (-0.383, 0.833) | 0.3176 | 0.631 (-0.369, 0.954 | 0.1790 |
| **DS-II** | -0.175 (-0.681, 0.443) | 0.5864 | -0.459 (-0.845, 0.239) | 0.1814 | -0.212 (-0.742, 0.482) | 0.5561 | -0.576 (-0.885, 0.084) | 0.0813 |
| **WCS** | 0.465 (-0.149, 0.820) | 0.1278 | 0.239 (-0.459, 0.755) | 0.5053 | 0.554 (-0.116, 0.878) | 0.0964 | 0.582 (-0.075, 0.887) | 0.0775 |
| **PCL-5** | -0.440 (-0.809, 0.179) | 0.152 | -0.641 (-0.905, -0.019) | 0.046 | -0.526 (-0.869, 0.154) | 0.1180 | -0.633 (-0.903, -0.006) | 0.0494 |

**Table I: Bivariate Fit of Change in Outcome Measures from Baseline to 2-week Endpoint by Mystical Experience Questionnaire (MEQ-30) and by the Nondual Awareness Dimensional Assessment-State Questionnaire (NADA-state) by study arm.** Analysis by study arm. No significant correlations found with the Challenging Experience Questionnaire (CEQ). Significant effects (**p < .05**) are bolded.

| **QIDS** | **MBI(EE)** | **MBI(DP)** | **MBI(PA)** | **DSII** | **WCS** | **PCL-5** |  |
| --- | --- | --- | --- | --- | --- | --- | --- |
| **QIDS** |  | 0.493 (**p=0.0198**) | 0.527 (**p=0.0118**) | -0.717 (**p=0.0026**) | 0.644 (**p=0.0012**) | -0.592 (**p=0.0037**) | 0.804 (**p<0.001**) |
| **MBI(EE)** | 0.493 (**p=0.0198**) |  | 0.701 (**p=<0.0001**) | -0.654 (**p=<0.0001**) | -0.413 (p=0.0558) | 0.064 (p=0.7772) | 0.550 (**p=0.0080**) |
| **MBI(DP)** | 0.527 (**p=0.0118**) | 0.701 (**p=<0.0001**) |  | -0.278 (p=0.3154) | 0.370 (p=0.0898) | 0.370 (p=0.0898) | 0.594 (**p=0.0036**) |
| **MBI(PA)** | -0.717 (**p=0.0026**) | -0.654 (**p=<0.0001**) | -0.278 (p=0.3154) |  | -0.572 (**p=0.0259**) | 0.523 (**p=0.0456**) | -0.663 (**p=0.0071**) |
| **DSII** | 0.644 (**p=0.0012**) | -0.413 (p=0.0558) | 0.370 (p=0.0898) | -0.572 (**p=0.0259**) |  | -0.703 (**p=0.0003**) | 0.637 (**p=0.0014**) |
| **WCS** | -0.592 (**p=0.0037**) | 0.064 (p=0.7772) | 0.370 (p=0.0898) | 0.523 (**p=0.0456**) | -0.703 (**p=0.0003**) |  | -0.633 (**p=0.0016**) |
| **PCL-5** | 0.804 (**p<0.001**) | 0.550 (**p=0.0080**) | 0.594 (**p=0.0036**) | -0.663 (**p=0.0071**) | 0.637 (**p=0.0014**) | -0.633 (**p=0.0016**) |  |

**Table J: Correlations between outcome measures from baseline to 2-week endpoint.** Significant effects (**p < .05**) are bolded.

| **Outcome** | **Raw p-value** | **FDR-adjusted p-value** |
| --- | --- | --- |
| WCS (GC) at 2 weeks | **0.005** | 0.055 |
| MBI (EE) at 6 months | **0.016** | 0.088 |
| DSII at 2 weeks | **0.029** | 0.105 |
| MBI (DP) at 2 weeks | **0.038** | 0.105 |
| MBI (DP) at 6 months | 0.067 | 0.124 |
| MBI (EE) at 2 weeks | 0.078 | 0.124 |
| WCS (GC) at 6 months | 0.090 | 0.124 |
| MBI (PA) at 2 weeks | 0.150 | 0.183 |
| MBI (PA) at 6 months | 0.260 | 0.276 |
| PCL-5 at 2 weeks | 0.079 | 0.124 |
| PCL-5 at 6 months | 0.276 | 0.276 |

**Table K: FDR-adjusted p-values for secondary outcome measures.** Significant effects (**p < .05**) are bolded.

| Effect | Num df | Den df | F | p-value |
| --- | --- | --- | --- | --- |
| Intercept | 1 | 14.97 | 4.46 | .052 |
| Treatment Group (MBSR+PAP vs. MBSR-only) | 1 | 18.34 | 11.98 | **.003** |
| Time (2 weeks vs. 6 months) | 1 | 20.00 | 0.18 | .678 |
| Treatment × Time Interaction | 1 | 20.00 | 5.77 | **.026** |
| Baseline QIDS-SR-16 Score | 1 | 18.08 | 6.61 | **.019** |

**Table L. Baseline-Adjusted Mixed Repeated Measures ANCOVA Results for QIDS-SR-16 Outcomes at 2-Week and 6-Month Follow-up**

Mixed-effects model with depression scores at 2-week and 6-month follow-ups as the dependent variable. Fixed effects included Treatment Group, Time, the Treatment × Time interaction, and baseline QIDS-SR-16 score as a covariate. Participant ID was included as a random intercept. Significant effects (p < .05) are bolded.

| Effect | Num df | Den df | F | p-value |
| --- | --- | --- | --- | --- |
| Intercept | 1 | 38 | 0.85 | .362 |
| Treatment Group (MBSR+PAP vs. MBSR-only) | 1 | 38 | 8.39 | **.006** |
| Time (2 weeks vs. 6 months) | 1 | 38 | 1.20 | .279 |
| Treatment × Time Interaction | 1 | 38 | 0.45 | .506 |
| Baseline MBI-EE Score | 1 | 38 | 4.83 | **.034** |

**Table M. Baseline-Adjusted Mixed Repeated Measures ANCOVA Results for MBI Emotional Exhaustion at 2-Week and 6-Month Follow-up**

Mixed-effects model with **MBI Emotional Exhaustion (EE)** scores at 2-week and 6-month follow-up as the dependent variable. Fixed effects included Treatment Group, Time, the Treatment × Time interaction, and **baseline MBI-EE score** as a covariate. Participant ID was included as a random intercept. Significant effects (**p < .05)** are bolded.

| Effect | Num df | Den df | F | p-value |
| --- | --- | --- | --- | --- |
| Intercept | 1 | 38 | 0.57 | .454 |
| Treatment Group (MBSR+PAP vs. MBSR-only) | 1 | 38 | 4.69 | **.037** |
| Time (2 weeks vs. 6 months) | 1 | 38 | 2.35 | .134 |
| Treatment × Time Interaction | 1 | 38 | 0.05 | .822 |
| Baseline MBI-DP Score | 1 | 38 | 11.57 | **.002** |

**Table N. Baseline-Adjusted Mixed Repeated Measures ANCOVA Results for MBI Depersonalization at 2-Week and 6-Month Follow-up**

Mixed-effects model with [insert outcome name, e.g., MBI-Depersonalization scores] at 2-week and 6-month follow-up as the dependent variable. Fixed effects included Treatment Group, Time, the Treatment × Time interaction, and **baseline MBI-DP score** as a covariate. Participant ID was included as a random intercept. Significant effects (p < .05) are bolded.

| **Effect** | **Num df** | **Den df** | **F** | **p-value** |
| --- | --- | --- | --- | --- |
| **Intercept** | 1 | 18.58 | 25.53 | **< .001** |
| **Treatment Group (MBSR+PAP vs. MBSR-only)** | 1 | 19.03 | 0.67 | .424 |
| **Time (2 weeks vs. 6 months)** | 1 | 19.40 | 0.01 | .912 |
| **Treatment × Time Interaction** | 1 | 19.40 | 0.29 | .597 |
| **Baseline MBI-PA Score** | 1 | 18.80 | 5.83 | **.026** |

**Table O. Baseline-Adjusted Mixed Repeated Measures ANCOVA Results for MBI Personal Accomplishment at 2-Week and 6-Month Follow-up**

Mixed-effects model with **MBI Personal Accomplishment (PA)** scores at 2-week and 6-month follow-up as the dependent variable. Fixed effects included Treatment Group, Time, the Treatment × Time interaction, and **baseline MBI-PA score** as a covariate. Participant ID was included as a random intercept. Significant effects (**p < .05)** are bolded.

| Effect | Num df | Den df | F | p-value |
| --- | --- | --- | --- | --- |
| Intercept | 1 | 16.90 | 10.15 | **.005** |
| Treatment Group (MBSR+PAP vs. MBSR-only) | 1 | 17.55 | 7.63 | **.013** |
| Time (2 weeks vs. 6 months) | 1 | 20.00 | 0.04 | .837 |
| Treatment × Time Interaction | 1 | 20.00 | 2.48 | .131 |
| Baseline WCS General Connectedness | 1 | 18.34 | 6.98 | **.016** |

**Table P. Baseline-Adjusted Mixed Repeated Measures ANCOVA Results for WCS General Connectedness at 2-Week and 6-Month Follow-up**

Mixed-effects model with **WCS General Connectedness** scores at 2-week and 6-month follow-up as the dependent variable. Fixed effects included Treatment Group, Time, the Treatment × Time interaction, and **baseline WCS General Connectedness** as a covariate. Participant ID was included as a random intercept. Significant effects **(p < .05)** are bolded.

|  | **QIDS.1** | **QIDS.2** | **QIDS.3** |
| --- | --- | --- | --- |
| **QIDS.1** | 15.5000 |  |  |
| **QIDS.2** | 4.069 | 14.488 |  |
| **QIDS.3** | 6.953 | 6.257 | 10.112 |

**Table Q. EM Covariances.** Little’s MCAR test: Chi-Square = 0.009, DF=1, Sig.= 0.925

**PER-PROTOCOL ANALYSIS:**

|  | | | | | | | | | | | | |
| --- | --- | --- | --- | --- | --- | --- | --- | --- | --- | --- | --- | --- |
|  | **QIDS** | | | **MBI_EE** | | | **MBI_DP** | | | **MBI_PA** | | |
| *Predictors* | *Estimates* | *CI* | *p* | *Estimates* | *CI* | *p* | *Estimates* | *CI* | *p* | *Estimates* | *CI* | *p* |
| (Intercept) | 11.73 | 9.60 – 13.85 | **<0.001** | 41.18 | 35.50 – 46.87 | **<0.001** | 17.64 | 13.75 – 21.52 | **<0.001** | 29.09 | 24.47 – 33.71 | **<0.001** |
| Time [2 Week] | -7.09 | -9.35 – -4.83 | **<0.001** | -15.27 | -21.42 – -9.13 | **<0.001** | -7.73 | -11.41 – -4.05 | **<0.001** | 8.00 | 3.45 – 12.55 | **0.001** |
| Time [6 Month] | -5.55 | -7.81 – -3.28 | **<0.001** | -21.21 | -27.55 – -14.87 | **<0.001** | -10.11 | -13.92 – -6.31 | **<0.001** | 7.67 | 2.98 – 12.37 | **0.002** |
| Group [2.MBSR + PAP] | 1.49 | -1.67 – 4.66 | 0.348 | 3.04 | -5.44 – 11.52 | 0.475 | 0.36 | -5.42 – 6.15 | 0.900 | 0.80 | -6.09 – 7.69 | 0.817 |
| Time [2 Week] × Group | 3.98 | 0.61 – 7.35 | **0.022** | 6.72 | -2.44 – 15.88 | 0.147 | 4.62 | -0.87 – 10.10 | 0.097 | -3.44 | -10.22 – 3.33 | 0.312 |
| Time [6 Month] × Group | 0.32 | -3.05 – 3.70 | 0.848 | 10.99 | 1.69 – 20.28 | **0.021** | 4.78 | -0.79 – 10.35 | 0.091 | -2.45 | -9.33 – 4.43 | 0.478 |
| Random Effects | | | | | | | | | | | | |
| σ^2^ | 7.00 | | | 51.55 | | | 18.49 | | | 28.20 | | |
| τ_00_ | 5.35 _participant_ | | | 36.68 _participant_ | | | 22.65 _participant_ | | | 30.13 _participant_ | | |
| ICC | 0.43 | | | 0.42 | | | 0.55 | | | 0.52 | | |
| N | 20 _participant_ | | | 20 _participant_ | | | 20 _participant_ | | | 20 _participant_ | | |
| Observations | 60 | | | 59 | | | 59 | | | 59 | | |
| Marginal R^2^ / Conditional R^2^ | 0.434 / 0.679 | | | 0.450 / 0.679 | | | 0.272 / 0.673 | | | 0.152 / 0.590 | | |

**Table R: Per-Protocol Analysis for Quick Inventory of Depressive Symptomatology (QIDS-SR-16) and Maslach Burnout Inventory – Human Services Survey for Medical Personnel (MBI-HSS-MP). Subscales: EE = emotional exhaustion; DP = depersonalization; and PA = personal accomplishment.** Mixed model analysis with group x time interactions at 2 week and 6 month endpoints. Participant as random effect. Significant effects **(p < .05**) are bolded.

| Outcome | Group | Contrast | Estimate | SE | *p*.value |
| --- | --- | --- | --- | --- | --- |
| QIDS | MBSR only | Baseline- 2 Week | 7.09 | 1.13 | **0.00** |
| QIDS | MBSR only | Baseline – 6 Month | 5.55 | 1.13 | **0.00** |
| QIDS | MBSR only | 2 Week – 6 Month | -1.55 | 1.13 | 0.37 |
| QIDS | MBSR + PAP | Baseline - 2.Week | 3.11 | 1.25 | **0.04** |
| QIDS | MBSR + PAP | Baseline - 6.Month | 5.22 | 1.25 | **0.00** |
| QIDS | MBSR + PAP | 2 Week – 6 Month | 2.11 | 1.25 | 0.22 |

**Table S: Simple effect for significant interaction results, per-protocol analysis.** Significant effects **(p < .05**) are bolded.

**Supplement 2: Group Psilocybin Intervention**

**Text A: Intervention Structure**

Our model employed a 1:1 participant to therapist ratio with an additional lead therapist role not assigned to any particular participant through a process of three group Preparatory Sessions, a single 8-hour group psilocybin dosing session, and three group Integration Sessions. This is the model our study team has employed in previous studies with demonstrated safety and feasibility. ^1,2^ The lead therapist led the group process during the preparation and integration sessions and provided an overseeing role during the psilocybin session. This role is important for several reasons: a) it allows individual therapists to focus primarily on the participant they were working with through what was a personally immersive process, b) it provides structure and a sense of leadership for the group of participants as a whole who have both the support from the group container but also from the dyad, c) it provides an additional layer of support for study therapists to process the experience, d) it ensures that the numerous practical necessities of the process were monitored from a perspective of more distanced objectivity and attention to the study demands. Study cohorts were 4-5 participants and groups were run with all individual therapists present as well as lead therapist and study PI in a large group space with chairs arranged in a circle around a central table with flowers and candles. Groups were 2 hours in duration with 1 hour of group process, 30 minutes of 1:1 break-out sessions with individual therapists where dyads would station themselves in semi-private areas of the group space and then 30 minutes of reconvened group process and check in. Music was played at the beginning and end of each preparatory and integration session over speakers. During the Integration Sessions the music drew from the dosing day playlist.

The break-out sessions into participant/therapist dyads was felt by participants to be a critical component of the intervention. This served to establish rapport, comfort, security/safety, and allowed for deeper exploration of autobiographical themes and intentions for the psilocybin session. We did not employ a formalized approach to assigning therapists however the team discussion about this prior to starting the intervention was an important part of our process to ensure the best fit based on available knowledge of participants throughout the screening process. Our protocol allowed for additional contact with the assigned therapist between group sessions as well as after the intervention and a majority of participants engaged with this, feeling this to be a helpful form of support.

*Group Process*

Groups were run in a supportive-expressive group therapy style which incorporated specific education and information during preparation regarding the psilocybin experience and dosing day. Groups focused on integrating mindfulness meditation techniques learned in the MBSR curriculum as well as sharing of personal experiences with symptoms of burnout and depression in relation to healthcare employment.

***Group Preparatory Sessions (3)***

Three group preparatory sessions were held over the course of a 1-week period. These were held on Monday, Thursday, and Friday prior to a Saturday dosing day. These preparatory sessions were 120 minutes in length, with 90 minutes designated for group process, and 30 minutes designated for 1-on-1 breakout sessions with assigned individual therapists. These groups included each participant (4-5), each individual therapist (4), a lead therapist, study PI, and a study coordinator.

The Preparatory Sessions focused on:

a. Establishing rapport, comfort, safety within the group and with therapist team

b. Psychoeducation regarding the study process and psilocybin subjective effects

c. Intention setting and sharing with the group

d. Mindfulness Practice with a 10-minute guided meditation at the beginning of each session.

d. Techniques for navigating the psychedelic experience (mantras such as “all is welcome”, “trust, let go, be open”, introduction of the concept of ‘inner healing intelligence’, a guided imagery session at the end of prep session #3 (see below), mindfulness techniques to stay grounded in the body.

e. Examination of themes related to burnout and depression.

f. Emphasis on collective support of the group itself, norming possible reactions during dosing day, emphasizing communal nature of the experience and interconnection of group member experiences.

During the 30-minute individual breakout portion of the preparatory groups each therapist/participant dyad moved to a different point in the auditorium. This period of time was intended to build comfort and safety with the assigned therapist, as well as to facilitate more in-depth exploration of personal narrative and history as well as deepen intention setting. Therapists were available to participants by phone for the duration of participation in the study, in case of need for additional support.

**Preparatory Session #1:**

***Assess Adverse Events (AEs) and concomitant medications.***

-10- minute guided group meditation (See Prep #1 Meditation)

-Introductions: each participant, therapist, and study team member introduces themselves, provides a little background as to why they are part of this study.

- Review of the timeline and logistics of the sessions through prep, dosing session, and integration. Review of the 1:1 assigned therapist model.   Review of the role of lead therapist.

-Discussion as to the importance of confidentiality for this intervention.

--Review of what to bring on dosing day (personal items, pillow, comfortable clothes, water bottle).

-Review of the ‘break out room’, rationale for this space, conditions that might merit use of this space.

-logistics of using the restroom, location of the restroom.

-Focus on intention setting. Discussion of initial intentions for involvement in the study. Introduced this concept with concomitant discussion as to the unpredictability of the psilocybin experience and importance of ‘letting go.’

-Review of required support person and collection of contact information for identified support person for transportation at the end of dosing day.

-Discussion and check-in regarding MBSR curriculum. Group sharing as to experience so far. Review of mindfulness skills that may be useful during the dosing session.

-Review of basics of psilocybin pharmacological effects including timing of effects and some possible subjective responses. Reviewed possible side effects and available PRN medications.

-Introduction of mantras that might be helpful during the dosing session: “trust, let go, be open”, “all is welcome”, “in and thru”.

-1:1 breakout session with individual therapists.

-Regroup as large group. Questions. Closing of group.

**Preparatory Session #2**

***Assess AEs and concomitant medications.***

-10-minute guided group meditation (see Prep #2 Meditation)

-Check in with participants.

-Review of any questions.

-Review intention setting.  Holding lightly.

-Review of personal items to bring if desired.

-Review of parameters on therapeutic touch (holding a hand or a hand on the shoulder only if indicated prior to the dosing day as a meaningful way of providing support). Review of how to communicate need for additional support during the dosing session. ,

-Review of music, the role of music in the dosing session, techniques to ground self with music, possibilities of aversive reactions to the music.

-Review of vital sign checks on dosing day.

-Review of required support person for transportation.

-Reviewed eating and drinking prior to dosing session, emphasizing having a light breakfast.

-Reviewed challenging experiences possible with psilocybin including suspiciousness, anxiety, fear, sexual feelings, ego death / dying.

-Discussion of the group nature of intervention, handling whatever arises in the room. Collective experience.

-Sharing on personal experiences and themes of burnout, depression, healthcare.

-How to contact team or therapist in between sessions, after dosing.

-1:1 breakout (30min)

--Regroup as large group. Questions. Closing of group.

**Preparatory Session #3**

***Assess AEs and concomitant medications.***

***Urine drug screen and pregnancy screen (if applicable)***

-10-minute group silent meditation

-Check in with participants.

-Review logistics for Saturday, time of arrival. Transportation and supervision.

-Review intentions with focus on both individual and group intentions.

-Discussed planning for rest of the weekend after the dosing session. Possible reactions post psilocybin experience including increased sensitivity, fatigue, emotionality. Reviewed reaching out to team if extra support needed.

-Discussed role and rationale for integration. Brief preview of what integration sessions look like.

-Open discussion, Q+A.

-1:1 breakout (30min)

-Regroup, discussion, questions.

-Guided Imagery (15 min) w music at end. (See Prep #3 Guided Imagery)

-Closing of group.

***Group Psilocybin Session***

The group psilocybin dosing day took place in the Huntsman Mental Health Institute auditorium Ion the Saturday following the last preparatory session. This space is partitioned into semi-private bays with inflatable mattresses on the floor. Each space had a vitals machine and a small designated area for personal items. This space was prepared prior to the session with decorative tapestries flowers, and personal items of significance to create a less sterile or medicalized ambiance. A restroom was immediately accessible to the space.

Participant/therapist dyads were stationed in the bays to allow for some degree of privacy while ensuring that all participants could easily hear each other within the room. An office space near by was prepared as a ‘break-out room’ in case a given participant had an experience that required increased privacy.

On the morning of the dosing day, participants were given time to settle into their spaces. Prior to administration of psilocybin we held a brief group session to check in with each participant, engage in a 10-minute silent meditation, review the timeline for the day, answer any questions, share intentions. Dosing was performed in a ceremonial manner without overt religious language with psilocybin capsules served to participants in decorated bowls. A music playlist was played over a speaker system in the room. This playlist was designed by a study team member and mixed into a single 6 hour piece of music without pauses or gaps between tracks. The playlist can be found here:

<https://lilnasy.synology.me:5001/as/sharing/7RBcFw9S/L211c2ljL1BBUFJfbWl4ZG93bl9maW5hbC5tcDM=>

Therapist engagement during the medicine session followed protocols similar to those employed in prior psilocybin-assisted therapy clinical trials with an emphasis on non-directive supportive interventions, and directing and reminding participants to move inwards. Light snacks and refreshments were made available later in the session. At approximately 6 hours post psilocybin administration, we reconvened as a group in the space for participants to share some brief reflections of their experience. Participants left the site with a pre-arranged support person and had the ability to contact their therapist by phone the following day (Sunday) but there was no formal contact until the first integration session (the following Monday). This delay until the first integration session reflected practical scheduling constraints rather than deep intentional design.

Participants completed questionnaires and assessments on site and had food and drinks available. Each participant was assessed by MD for psychiatric and medical safety to leave the site, including a Columbia-Suicide Severity Rating Scale and AE assessment. Transportation with pre-identified support person was facilitated with a direct hand off and engagement with support person.

***Group Integration Sessions (3)***

The first integration session took place on the Monday following the Saturday dosing day and the integration period extended a total of 2 weeks with sessions on Monday, Friday, and the following Friday. These sessions (120 minutes each, again with a 30 minute ‘break-out’ session with assigned individual therapists) were held in the same auditorium at the Huntsman Mental Health Institute using the same format as the preparatory sessions with participants and therapists arranged in a circle. Again, the lead therapist led the group process with input from other study therapists. Integration Sessions followed a less structured format than Preparatory Sessions given the heterogeneity in group needs and response and intention to meet these therapeutic requirements in a flexible and adaptable fashion.

Integration Sessions included the following points of emphasis:

1. Individual sharing of experiences. Reflections on the experiences of other participants.
2. Incorporation of mindfulness meditation at the beginning of each Integration Session (however at beginning of Integration Session #1 we had the group listen to a track from the session playlist instead of a group meditation).
3. Revisiting of intentions and how these surfaced or didn’t surface during the experience.
4. Revisiting themes of depression, burnout, existential issues. Personal life narrative related themes.
5. Exploration of spiritual themes that arose.
6. Reflection and exploration of the group experience during the session.
7. Strategies for ongoing integration of insights and psychological material including mindfulness practices, returning to the music playlist, journaling/writing/artwork/other creative expressions.
8. Reflections on the temporary nature of this group and the experience itself, the impending end of the group process.
9. Reflections on how, when, and if to share this experience with others in participants’ lives: partners, friends, etc.
10. Gathering feedback as to the group format and protocol.

**Preparatory Session #1 Guided Meditation (10 minutes)**

**(adapted from the guided meditation ‘Rest and Allow’ by Stephen Bodian on the Waking Up app)**

Take a few minutes to sit comfortably. Allow your body and mind to settle.

Instead of focusing on the breath, or on any object in particular I’d like to invite you to just sit quietly and just let everything be the way it is. Don’t make a big effort to focus, don’t manipulate your attention, don’t follow your breathing, don’t do anything in particular.

Just let everything be without trying to change, avoid, or get rid of anything.

You may find this difficult. In fact, your mind isn’t really good at this. It is good at holding on, perhaps good at focusing. Not as good at letting go. Just sit with this, with this lack of need to do anything. (Pause) Maybe there is a way where this letting go has already happened. Already there, in a way. A natural state of openness. So you just need to drop back into this openness, this letting go, that is always already available.

Think of clouds in the sky. The sky doesn’t have to do anything to hold them: by it’s nature it is open, all-inclusive. Clouds come and go.

This may be confusing. We are so used to using our attention. Our minds are so accustomed to holding on, to doing. Not so much to letting go.

When you hear this instruction to ‘let go’ you might feel like this something special for you ‘to do’. Some kind of task or project that will have certain outcomes. Instead, see this as an invitation to rest in this openness that is already taking place. No goals, no agenda.

When your mind focuses in on objects, or thoughts, you may experience this as a kind of contraction. Just notice that contraction and then relax, drop back into that condition where all of these phenomena are just coming and going.

What is this moment like when there is no project, no goal?

This space of awareness is silent and always present independent of the contents that are arising and passing away. Let yourself rest as this silent, open awareness. No cultivation, no doing, no need to constantly check to see if you are doing it the right way. Let everything be exactly as it is, inside and outside.

Just rest in this space.

Once you have settled into this space I’d like you to take a few moments to bring to mind another person in this group. Another participant. Maybe you have already made a connection with this person in the MBSR groups, maybe you haven’t interacted with them at all. But bring them to mind, all of your impressions and feelings. And I’d like you to direct positive wishes of wellbeing towards them: may you be happy, may you be at peace, may this process be helpful for you, may it offer you healing. Just repeat those phrases internally as you bring this person to mind. And really connect with those feelings, with your genuine wish that this person be free from suffering, find the healing that they need. Notice changes in your mind and in your body as you direct this energy towards this person. I’d like you now to bring yourself to mind and direct these same thoughts to yourself: may I be happy, may I be at peace, may I find the healing I need. This can be harder to direct these well wishes towards ourself. Notice that just being here is already an expression of this loving intention towards yourself.

**Text B: Guided Meditations and Guided Imagery**

**Preparatory Session #2 Guided Meditation**

Sit comfortably. See if you can sit a little straighter, a little more erect, with a straight spine. Close your eyes. Take a few deep breaths. Now bring to mind someone in the group that you feel like it is easy to feel loving kindness for. You’ve gotten to know each other through this process so far, and even if you don’t know this person deeply, you have a sense of what they are carrying, and what you might hope for them through this process. Maybe there is one person that initially comes to mind- either someone that you have resonated with or connected with more deeply, or maybe not. I want you to think positive thoughts towards this person. May you be happy. May you be free from suffering. May you be safe. May you be at peace.

Direct these intentions towards this individual in your mind. Notice that you really want this person to be happy, that you really do hold this wish for this person. If you could ease their suffering right now you would do that.

May you be happy. May you be free from suffering.

See if you can just follow these intentions down the rabbit hole. Notice any changes in the body that happen for you as you follow these intentions down. See if you can notice a feedback loop here and dive deeper into that.

See if you can bring to mind some of what you know of this person’s suffering. Maybe there is a way they have shared here or in MBSR that has stuck with you, or that you have resonated with. Connect with your desire to alleviate that suffering. And radiate this wish. May you be at peace, may you be free from suffering. May you be free of fear or worry. You are all healthcare providers, and engaged deeply in the project of alleviating suffering in others. But I want you now to really connect with that wish for this specific person. Notice the specifics that come to mind, notice who this person is, how they carry this.

Now I’d like you to bring to mind another member of the group. Just notice who comes to mind and what strikes you. Again, notice the specifics. It can be surprising how much we notice, how much we know. And feel how you can welcome ALL of it. This person, too, has a unique burden that is all their own. And radiate these loving intentions: may you be happy. May you be free from suffering. May you be at peace. Feel your desire for them to be happy. Imagine yourself radiating this out like light. Feel what you would wish for them in our experience tomorrow. Really feel your desire for them to have the experience that they need to have in our group psilocybin experience. Really connect with these wishes.

Feel these intentions for our group. Include in this all of the participants and the therapist and study team. See if you can radiate these well wishes like the sun. Connect with your intentions for our group tomorrow and as we go forward through this process. We are embarking on a unique journey together. And everyone is playing a critical role with this process. Your intentions towards each other are playing a critical role with this process. Everyone is carrying exactly what they need to be carrying for this process together. Expand the scope of these intentions to include the whole hospital system. All of your colleagues, all of the patients coming and going in the ED. Really direct your intentions towards all of this. May you be at peace, may you be free from suffering.

Finally, I’d like you to turn your focus on yourself. Directing loving kindness towards ones’ self can be more difficult than towards others. If this is the case, just notice this difficulty and hold it gently. Hold it with compassion for yourself. Maybe notice how hard you have been trying, how hard you have been working. All that you have been holding. Maybe you’ve been holding this for a long time. See if you can bring to mind yourself as a young child. There is a history here for your own challenges, your own suffering. Review the journey you’ve had in your life to be right where you are right now: all the joys and the sorrows. Direct these loving wishes towards yourself: May I be happy, may I be healthy, may I be at peace, may I be free from suffering. Recognize that being here right now, being a part of this project, is already an expression of this wish for yourself. Bring to mind your intentions that you have been cultivating through this process and connect with this wish, these desires. Feel that our group is holding this space for you, supporting these intentions for you. Feel that these specific intentions you have are also – in a way - supporting the group.

Preparatory Session #3 Guided Imagery

“Red Butte Garden” (this is a public garden adjacent to the Huntsman Mental Health Institute and well known to locals).

*I invite you to close your eyes, steady your breathing, in and out through your nose,*

*slowly. Bring your attention to the tip of your nostrils and how the air feels cooler here on thein- breath and warmer on the out-breath. Keep your focus on this feeling in your nostrils as you breathe in and out. Keep breathing slowly.*

*Notice sensations in the body, sensations of sitting. Feet on the ground. Back against the chair. Notice sounds in the room. Notice sounds coming and going. Notice that you can’t control the sounds, they come and go on their own.*

*I’d like you to imagine that you are taking a walk through Red Butte Garden. Yes, Red Butte Garden. We are all going there as a group. All of us sitting here today. A field trip of sorts. You have probably been there many times. In fact, it is right over there. We can see it out of the window here, right down the road. Right in front of our noses. We aren’t going to visit tulip fields in the Netherlands, or Buckingham palace, or Narnia, or the garden of Eden. Just Red Butte garden. Notice that that place, that location perhaps brings up a range of memories or associations. Maybe visiting with family. Maybe just a familiar name. Close to where you work. Spend a few moments noticing the thoughts, memories, emotions, associations that come up with Red Butte Garden, with this familiar place.*

*You have a map of the garden. You can see the various sections and areas. You have ideas as to what may be in store for you there, perhaps you have visited these sections before. It is all laid out for you on the map which has names, plant names, species. As a group we point to the map, discuss. Consider pros and cons of various areas. We plan our adventure together.*

*I invite you to now approach a specific area within Red Butte garden and bring your attention closer, more focused. Bring your attention to the feeling of your footsteps along the gravel path. Notice the sounds of your feet, moving slowly and deliberately. Our group is still together, we are in the same area but our attention is becoming more focused, perhaps in different directions. Notice the feelings in your feet and legs as you move deliberately towards a specific group of flowers. Notice the sun on your back, your neck, your head. You can feel the others in the group moving to their own distinct areas. Notice you still feel connected to them. Everyone is in the right place for them. Feel the warmth of the sun, the radiance. Notice a coolness in the air that you can feel at the same time as this sensation of heat. Notice how it shifts almost moment by moment as you walk through shade back out into sunlight.*

*Your attention is now centered on a single, specific flower in the garden. Look closely. Notice the texture of the petals, the colors, perhaps grains of pollen. Maybe you know the name of this flower, you have seen them many many times before. You know some details about its biology, its habitat. Notice the green stalk, leaves, stem. You are still aware of the rest of the group who are around you, but also now increasingly immersed in their own exploration. There is curiosity here. Notice that all the parts of the flower support all the parts of the flower: without the roots there is no stem, without the stem there can be no leaves, without all of this there is no flower. Bring your attention very close. The other sounds of the garden are quiet now. Maybe you feel a growing sense of intimacy here. Bring your face closer. What is the smell? What does that evoke for you? Are there memories there? Emotions? Notice that these smells are occurring in the same space as the visual experience of the flower, in the same space as your thoughts and feelings. Just stay with this, whatever is coming up. Stay with the bare, raw details of what you are experiencing. What is this experience like when you are not labeling it, categorizing it? What is this experience like when it doesn’t have a name?*

*Is there liking or disliking here? Maybe a pleasant smell or sensation or mental associations. Or maybe a brief unpleasant memory that flickers through the mind or a chill in the air with a passing cloud. What is this experience like if you don’t have to like or dislike anything?*

*Notice that this experience is not on the map.*

*Gently reach out a fingertip. With the most gentleness you can muster, slowly touch the stem. What is the texture? What is this relationship between your finger, the plant, and this experience? Where does your finger end and the plant begin? Close your eyes and see if you can feel only the raw sensory experience of touching this plant. Where is this experience happening? Bring your fingers gently up to the flower itself. So delicate but also with a surprising strength. This flower survives rain, wind, the elements, animals. Bring your hand down to the soil at the base of the flower. Press your hand into the dirt, dark, rich, full of nutrients. Is there a smell here? Are there insects? Animal tracks, animal bones? Decomposing vegetable matter? Take in the richness of this soil. And feel how it provides the conditions for the flower to grow, to exist, to flower.*

*Just stay here with these sensations…*

*Allow yourself to feel the transitory nature of this flower. It is here now but maybe even in a few days it will be changed. Later in the season it will be becoming part of the soil. Stay with this now and fully feel all of the sensations with this awareness in the background.*

*Lift your head from this specific flower. You have been developing a relationship with this specific flower, all of its unique attributes. But notice that there are other flowers around it, many look similar but they are not identical. And adjacent to those are different flowers, different plants. Look further afield and see trees, other plants, see the trees and plants outside of the garden, the sage brush off the shoreline trail, the scrub oak leading up the foothills.*

*You survey the area around you and see your group members. Some are still immersed headlong with their flower, or tree, or rock. Some are walking around looking at the sky. One person is lying on their back, eyes closed. Gradually, slowly, silently, the group reconvenes. There are smiles but not a lot of talking. Slowly we begin our walk back down the gravel path, out through the gates of the garden. Of Red Butte Garden.*

**Integration #1:**

No specific guided meditation but listened as a group to this track from the dosing day playlist: ”Baba Hanuman” by Krisha Das https://open.spotify.com/track/468YB4iLb2YJyncuHGJ62i?si=391365603b774b2d

**Integration #2 Meditation**

Sit comfortably. Close your eyes, or leave them open if you would prefer and with a gentle gaze a short distance in front of you. Bring your attention to the breath and the sensations of breathing for a few breaths.

I’d like you to feel the presence of another participant in this experience. You know some things about their experience on Saturday at this point. You know some things about what they have been carrying. Feel your compassion for this person.

Feel our group as a whole, sitting here. Just this moment right now. Notice the emotions or thoughts or feelings that come up. Notice all of it, all of the specifics with each other. This may include feelings of connection, of openness, of compassion, of sympathetic joy or sorrow. Your wishes for them going forward. This may also include some feelings of awkwardness or distance. Or questions. Or **doubt**. We are all coming to this group with our own idiosyncratic selves. We are all works in progress. Is there something you are not welcoming here right now? Can we welcome even loneliness, sorrow? Can we welcome doubt? Can we welcome all of it?

Bring to mind the journey we have taken as a group. And the journey with MBSR this is embedded in. Recognize the changes in yourself. They may be big. They may be small. Maybe there is just a seed here: a possibility of change. Ask yourself: what is important to me in this group today? How would I like to show up here today in this group in light of this experience, in light of this change, in light of what I am working on? In light of cultivating this seed?

Bring to mind the fact that this group will end soon, and notice whatever feelings this brings up for you. These feelings will also change: maybe they will fade, shift, evolve, unfold. This space can hold all of it.

Bring to mind your experience last Saturday. There were many parts to this, many chapters, many places visited on that journey. What comes up in your heart? Is there an experience or a feeling tone there that feels important to you? That you feel is important to connect with, or carry forward in some way? Move deeper into that. Notice the feelings in your body. See if you can let go and fall back into that space. Notice if there is any grasping or reaching for this. Can you drop that too? Was there a core to this experience for you?

What does this ask of you? How will you carry this forward from here? Is there a way you have already been carrying it?

What does this ask of you?

(Play Music)

**Integration #3 Meditation**

10 minutes of silent group meditation.

**Text C: Citations**

1. Lewis BR, Garland EL, Byrne K, et al. HOPE: A Pilot Study of Psilocybin Enhanced Group Psychotherapy in Patients With Cancer. *Journal of Pain and Symptom Management*. 2023;0(0). doi:10.1016/j.jpainsymman.2023.06.006

2. Lewis BR, Byrne K, Hendrick J, Garland EL, Thielking P, Beck A. Group format psychedelic-assisted therapy interventions: Observations and impressions from the HOPE trial. *Journal of Psychedelic Studies*. 2023;1(aop). doi:10.1556/2054.2022.00222
