# Supplementary material for: Psilocybin-assisted group psychotherapy and mindfulness-based stress reduction for frontline healthcare provider COVID-19-related depression and burnout: A randomized controlled trial
Source: PLoS Med. 2025 Sep 19;22(9):e1004519. doi: 10.1371/journal.pmed.1004519 (PMC12459851; doi:10.1371/journal.pmed.1004519)
Supplement: S1 — Full study protocol and statistical analysis plan. (DOCX) [file pmed.1004519.s002.docx]

*Study Protocol*

**PAPR: Psilocybin-assisted psychotherapy + Mindfulness-based Stress Reduction (MBSR) for front-line healthcare provider COVID-19-related depression and burnout.**

**Protocol Version 7.0**

**April 2024**

*Investigator Data:*
Benjamin R. Lewis MD; John Hendrick MD – Principal Investigators

Eric Garland PhD – Co-Investigator

Kevin Byrne MD – Co-Investigator

Chris Gregg PhD – Co-Investigator

**PAPR: Psilocybin-assisted psychotherapy + Mindfulness-based Stress Reduction (MBSR) for front-line healthcare provider COVID-related burnout.**

| **Principal Investigators** | Benjamin R. Lewis MD. Huntsman Mental Health Institute, University of Utah. 501 Chipeta Way, Salt Lake City, UT. 84108. b[en.lewis@hsc.utah.edu](mailto:Ben.lewis@hsc.utah.edu)  John Hendrick MD. University of Utah Department of Emergency Medicine. 50 North Medical Drive, Salt Lake City, UT 84108. j[ohn.hendrick@hsc.utah.edu](mailto:John.hendrick@hsc.utah.edu) |
| --- | --- |
| **Statistician** | Chaorong Wu PhD |
| **Medical Monitor** | TBD |
| **Drug Manufacturer** | Usona Institute  2800 Woods Hollow Rd  Madison. WI 53711-5399 |
| **Investigational agent(s)** | Psilocybin |
| **IND Number** | 163040 |
| **NCT Number** *(CT.gov)* |  |

**TABLE OF CONTENTS**

Page

[LIST OF ABBREVIATIONS 3](#_Toc1846148171)

[PROTOCOL SIGNATURE 7](#_Toc1636847923)

[STUDY SUMMARY 8](#_Toc97624379)

[1 OBJECTIVES 14](#_Toc87503341)

[1.1 Primary Objectives 15](#_Toc1011719088)

[1.2 Secondary Objectives 15](#_Toc1626081743)

[1.3 Exploratory Objectives 16](#_Toc612150980)

[2 BACKGROUND 17](#_Toc326438347)

[2.1 Psilocybin 20](#_Toc1951789972)

[2.2 Design Rationale 22](#_Toc802794736)

[3 DRUG INFORMATION 24](#_Toc1561210315)

[3.1 Psilocybin 24](#_Toc1820999982)

[4 STUDY DESIGN 26](#_Toc1217363275)

[4.1 Description 26](#_Toc908786977)

[4.2 Number of Patients 27](#_Toc1445542814)

[4.3 Number of Study Centers 27](#_Toc144616476)

[4.4 Study Duration 27](#_Toc1665562547)

[5 ELIGIBILITY CRITERIA 27](#_Toc376586589)

[5.1 Inclusion Criteria 27](#_Toc1405930065)

[5.2 Exclusion Criteria 29](#_Toc240241357)

[5.3 Recruitment Strategies 30](#_Toc943166014)

[6 TREATMENT PLAN 31](#_Toc65687785)

[6.1 Treatment Schedule 31](#_Toc1125788958)

[6.2 Psilocybin 33](#_Toc1249744861)

[6.3 Concomitant Medications and Therapies 34](#_Toc2005800604)

[6.4 Duration of Therapy 35](#_Toc1230510529)

[7 TOXICITIES AND DOSE MODIFICATION 36](#_Toc1914965340)

[7.1 Guidelines for the Management of Adverse Events 36](#_Toc1770430526)

[7.2 Contraception 38](#_Toc1703795445)

[8 SCHEDULE OF EVENTS 38](#_Toc468838725)

[9 STUDY PROCEDURES 43](#_Toc1215792539)

[9.1 Screening 43](#_Toc1578995857)

[9.2 Randomization and study arm assignment 43](#_Toc240754214)

[9.3 MBSR Arm 43](#_Toc1523431037)

[9.4 MBSR + PAP arm 44](#_Toc1777387147)

[10 STUDY ASSESSMENTS 46](#_Toc486959029)

[10.1 Physical Examinations and Vital Signs 46](#_Toc251410618)

[10.2 Adverse Events 47](#_Toc70699184)

[10.3 COVID-19 Precautionary Measures 47](#_Toc1496735536)

[10.4 Monitoring for Suicide Ideation 48](#_Toc1430515591)

[10.5 Laboratory Assessments 48](#_Toc428145328)

[10.6 Response Assessment 49](#_Toc101057612)

[11 CRITERIA FOR EVALUATION AND ENDPOINT 49](#_Toc2066619721)

[11.1 Safety 49](#_Toc417139620)

[11.2 Efficacy 49](#_Toc870883486)

[11.3 Stopping Rules 50](#_Toc1695734902)

[12 STATISTICAL CONSIDERATIONS 50](#_Toc237344039)

[12.1 Population for analyses 50](#_Toc1189628221)

[12.2 Statistical Analyses 50](#_Toc1936058804)

[Storyline Integrative Assessment - Neurological, Psychiatric, Cognitive and Psychological 56](#_Toc989370645)

[13 REGISTRATION GUIDELINES 57](#_Toc20073928)

[14 DATA SUBMISSION SCHEDULE 57](#_Toc2097459772)

[15 ETHICAL AND REGULATORY CONSIDERATIONS 58](#_Toc1416550487)

[15.1 Human Subject Protections 58](#_Toc1740166358)

[15.2 Institutional Review 58](#_Toc120186727)

[15.3 Data and Safety Monitoring Plan (see DSMB protocol) 58](#_Toc822559573)

[15.4 Storyline Health Data Confidentiality and Security 60](#_Toc1684485963)

[15.5 Adverse Events and Serious Adverse Events 61](#_Toc4433957)

[15.6 SAE Reporting Requirements. 63](#_Toc604159865)

[Minimum data to be reported via Usona forms for SAEs. 64](#_Toc1168490235)

[Initial report 64](#_Toc614194531)

[Follow-up report: Delivery 64](#_Toc1717320086)

[15.7 Reporting of Pregnancy 65](#_Toc598707453)

[15.8 Protocol Amendments 66](#_Toc1362511985)

[15.9 Protocol Deviations 66](#_Toc702119096)

[15.10 FDA Annual Reporting 66](#_Toc150507996)

[15.11 DEA Registration and Reporting 66](#_Toc1622358015)

[15.12 Clinical Trials Data Bank 67](#_Toc138447448)

[15.13 Record Keeping 67](#_Toc83579010)

[16 BIBLIOGRAPHY 67](#_Toc220165571)

[16.1 Key References 68](#_Toc1960831732)

[Assessment Components 72](#_Toc92145306)

[PAPR Preference/ Expectancy 104](#_Toc534833713)

[VISUAL ANALOGUE SCALE 105](#_Toc286881829)

**List of Figures**

[Figure 1: Study Schema **Error! Bookmark not defined.**](#_Toc51168772)

[Figure 2. Psilocybin molecular structure 26](#_Toc51168773)

**List of Tables**

[Table 1: Adverse Event Management 38](#_Toc51168774)

[Table 2: Schedule of Events for the Psilocybin Arm 41](#_Toc51168775)

[Table 3: Laboratory Assessments 49](#_Toc51168776)

**List of Appendices**

[Appendix 1: Storyline Assessments 73](#_Toc179272918)

[Assessment Components 73](#_Toc179272919)

[16.1.1.2 The Caterpillar Question 75](#_Toc179272920)

[16.1.1.3 Storyline Association - 4 Question Set 76](#_Toc179272921)

[Storyline ASQ-5 + Video v2 76](#_Toc179272922)

[PCL-C Questionnaire 77](#_Toc179272923)

[PHQ-9 Questionnaire 78](#_Toc179272924)

[GAD-7 Questionnaire 78](#_Toc179272925)

[Basic Neurological Symptoms  Questionnaire 78](#_Toc179272926)

[Mental Health and Addiction Symptoms Questions 78](#_Toc179272927)

[Appendix 1: Quick Inventory of Depressive Symptomatology (Self-Report) (QIDS-SR16) 80](#_Toc179272928)

[Appendix 2: MBI Human Services Survey for Medical Personnel 84](#_Toc179272929)

[Appendix 3: Demoralization Scale-II (DS-II) 85](#_Toc179272930)

[Appendix 4: PCL-5 86](#_Toc179272931)

[Appendix 5: FFMQ-15 87](#_Toc179272932)

[Appendix 6: TMS 88](#_Toc179272933)

[Appendix 7: Nondual Awareness Dimensional Assessment (NADA) (trait) 90](#_Toc179272934)

[Appendix 8: Mystical Experience Questionnaire (MEQ30) 91](#_Toc179272935)

[Appendix 9: Brief Savoring Inventory 94](#_Toc179272936)

[Appendix 10: Patient-Reported Experience on Study Therapy 95](#_Toc179272937)

[Appendix 11: PHQ-9 96](#_Toc179272938)

[Appendix 12: QIDS-SR-16 97](#_Toc179272939)

[Appendix 13: Challenging Experience Questionnaire 99](#_Toc179272940)

[Appendix 14: Watts’ Connectedness Scale (WCS) 101](#_Toc179272941)

# LIST OF ABBREVIATIONS

| Abbreviation or Term^1^ | Definition/Explanation |
| --- | --- |
| AE | Adverse event |
| ALT | Alanine aminotransferase |
| ANCOVA | Analysis of covariance |
| ANOVA | Analysis of variance |
| APTT | Activated partial thromboplastin time |
| AST | Aspartate aminotransferase |
| AV | Atrioventricular |
| β-HCG | Beta-human chorionic gonadotropin |
| BID | Twice daily |
| BLQ | Below limit of quantification |
| BMI | Body mass index |
| BP | Blood pressure |
| BUN | Blood urea nitrogen |
| Ca^++^ | Calcium |
| CBC | Complete blood count |
| CFR | Code of Federal Regulations |
| CHF | Congestive heart failure |
| CI | Confidence interval |
| Cl- | Chloride |
| CL_cr_ | Creatinine clearance |
| C_max_ | Maximum observed concentration |
| C_min_ | Trough observed concentration |
| CNS | Central nervous system |
| CR | Complete response |
| CRF | Case report form |
| CT | Computed tomography |
| CTCAE | Common Toxicity Criteria for Adverse Events |
| CV | Coefficient of variation |
| CYP | Cytochrome P450 |
| D/C | Discontinue |
| DEA | Drug Enforcement Administration |
| ECOG | Eastern Cooperative Oncology Group |
| eCRF | Electronic case report form |
| DLT | Dose Limiting Toxicity |
| ECG | Electrocardiogram |
| Eg | Exempli Gratia (for example) |
| FACS | Fluorescence-Activated Cell Sorting |
| FDA | Food and Drug Administration |
| FDG-PET | Fluorodeoxyglucose (FDG)-positron emission tomography (PET) |
| GCP | Good Clinical Practice |
| GFR | Glomerular filtration rate |
| GGT | Gamma-glutamyltransferase |
| GLP | Good laboratory practice |
| HBsAg | Hepatitis B surface antigen |
| HBV | Hepatitis B virus |
| HCO_3_^-^ | Bicarbonate |
| HCV | Hepatitis C virus |
| HIV | Human immunodeficiency virus |
| HR | Heart rate |
| hr | Hour or hours |
| IC_50_ | Half maximal inhibitory concentration |
| i.e. | Id est (that is) |
| IEC | Independent ethics committee |
| IND | Investigational New Drug |
| INR | International normalized ratio |
| IRB | Institutional review board |
| IU | International unit |
| IV | Intravenous, intravenously |
| LDH | Lactate dehydrogenase |
| LLQ | The lower limit of quantitation |
| MedDRA | Medical Dictionary for Drug Regulatory Activities |
| MRI | Magnetic resonance imaging |
| MRSD | The maximum recommended starting dose |
| MTD | Maximum tolerated dose |
| NOAEL | No-observed-adverse-effect level |
| NOEL | No-observed-effect-level |
| PD | Pharmacodynamic(s) |
| PFS | Progression-Free Survival |
| PK | Pharmacokinetic(s) |
| PO | Per os (administered by mouth) |
| PR | Partial response |
| PT | Prothrombin time |
| PTT | Partial thromboplastin time |
| QC | Quality control |
| RBC | Red blood cell |
| QD | Once-daily |
| QTc | QT interval corrected |
| QTcF | QT interval corrected using Fredericia equation |
| SAE | Serious adverse event |
| SD | Standard deviation or stable disease |
| T_1/2_ | Terminal elimination half-life |
| T_3_ | Triiodothyronine |
| T_4_ | Thyroxine |
| T_max_ | Time of maximum observed concentration |
| TID | Three times daily |
| TSH | Thyroid-stimulating hormone |
| ULN | The upper limit of normal |
| ULQ | The upper limit of quantitation |
| UV | Ultraviolet |
| WBC | White blood cell |
| WOCBP | Women of childbearing potential |
| WONCBP | Women of nonchildbearing potential |

^1^ All of these abbreviations may or may not be used in the protocol.

# PROTOCOL SIGNATURE

I confirm that I have read this protocol, and I will conduct the study as outlined herein and according to the ethical principles stated in the latest version of the Declaration of Helsinki, the applicable ICH guidelines for good clinical practice, and the applicable laws and regulations of the federal government. I will promptly submit the protocol to the IRB for review and approval. Once the protocol has been approved by the IRB, I understand that any modifications made during the course of the study must first be approved by the IRB prior to implementation except when such modification is made to remove an immediate hazard to the subject.

I will provide copies of the protocol and all pertinent information to all individuals responsible to me who assist in the conduct of this study. I will discuss this material with them to ensure that they are fully informed regarding the study treatment, the conduct of the study, and the obligations of confidentiality.

Note: This document will be signed electronically through submission and approval by the Principal Investigator in the University of Utah IRB Electronic Research Integrity and Compliance Administration (ERICA) system.

# STUDY SUMMARY

| Title | PAPR: Psilocybin-assisted psychotherapy (PAP) + Mindfulness-based Stress Reduction (MBSR) for front-line healthcare provider COVID-related depression and burnout. |
| --- | --- |
| Short Title | PAPR |
| Protocol Identifiers (IRB – internal) |  |
| IND number | 163040 |
| Phase | Pilot |
| Design | Open-label, pilot trial. |
| Study Duration | 1.0 years |
| Study Center(s) | This is a single-center trial to be conducted at Huntsman Mental Health Institute |
| Objectives | **Primary Objectives**   - To assess the safety and tolerability of psilocybin in the study population. - To assess the feasibility to recruit, consent, and enroll healthcare providers in a study of psilocybin-assisted therapy + MBSR vs. MBSR and for those participants to remain in the study until completion. - To assess the effect that psilocybin-assisted therapy has on depression symptoms in healthcare providers with burnout as measured by the QIDS-SR-16.   **Secondary Objectives**   - To assess the changes in rates of provider burnout as measured by the Maslach Burnout Inventory for Medical Professionals (MBI-HSS (MP)) both in terms of rates of meeting criteria for burnout as well as mean reduction in score on the MBI. - To assess the long-term effect that psilocybin-assisted therapy and MBSR have on depression and anxiety symptoms in the study population. - To assess the effect psilocybin therapy and MBSR have on symptoms of post-traumatic stress syndrome. - To assess the effect psilocybin therapy and MBSR have on participant reported quality of life. - To assess the effect psilocybin therapy and MBSR have on participant reported feelings of connectedeness to self, other, and world.   **Exploratory Objectives**   - To assess the effect psilocybin therapy, in conjunction with an MBSR curriculum, has on state and trait mindfulness measures. - To assess participant-reported psilocybin experience on the day of medication administration and to assess the relationship between reported experience and depression and burnout response. - To use the Storyline Health AI platform to develop a model for precision research on psychedelic-assisted therapies and healthcare provider burnout and to better characterize predictors of response to mindfulness training and psilocybin therapy utilizing the Storyline Integrative Assessment tool. |
| Number of Subjects | 25 |
| Diagnosis and Main Eligibility Criteria | **Key Inclusion Criteria:**   - Male or female healthcare providers aged ≥ 25 years old. - Participants must be physicians or nurses with at least 1 month of frontline clinical experience during the COVID-19 pandemic. - Screening MBI-HSS (MP) score that meets our working definition of burnout (emotional exhaustion subscale ≥ 27) and a ‘high’ score on one other subscale (either depersonalization ≥ 13 or personal accomplishment ≤ 21). - PHQ-9 depression screen ≥ 10. - Not taking regularly scheduled medications to treat depression and/or anxiety, including benzodiazepines, for at least 4 weeks prior to initiation of the study.   **Key Exclusion Criteria:**   - Prior systemic antidepressants, anti-psychotic, or anxiolytic medication within four weeks prior to psilocybin administration. - Personal history or immediate family members with schizophrenia, bipolar affective disorder, delusional disorder, schizoaffective disorder, psychosis, dementia, or other psychotic spectrum illness. - Currently meeting DSM-5 criteria for Dissociative Disorder, or other psychiatric conditions judged to be incompatible with the establishment of rapport or safe exposure to psilocybin. - Currently meeting DSM-5 criteria for Cluster B Personality Disorder). - Current or history within the last two years of meeting DSM-5 criteria of substance use disorder (excluding caffeine and nicotine). |
| Study Product, Dose, Route, Regimen | Psilocybin 25 mg PO |
| Duration of administration | One-time administration |
| Reference therapy | MBSR |
| Statistical Methodology | Feasibility will be assessed by the ability to recruit, consent, and enroll 24 patients in the trial, and for at least 16/24 patients to remain in the study until completion.  The key secondary analysis of QIDS-SR-16 and MBI-HSS(MP) will use mixed-effects models with fixed effects for time (enrollment, 2 weeks post psilocybin), and a random intercept term. The outcome variables are the estimated change from baseline to 2 weeks post psilocybin from these models. |

**Schema**


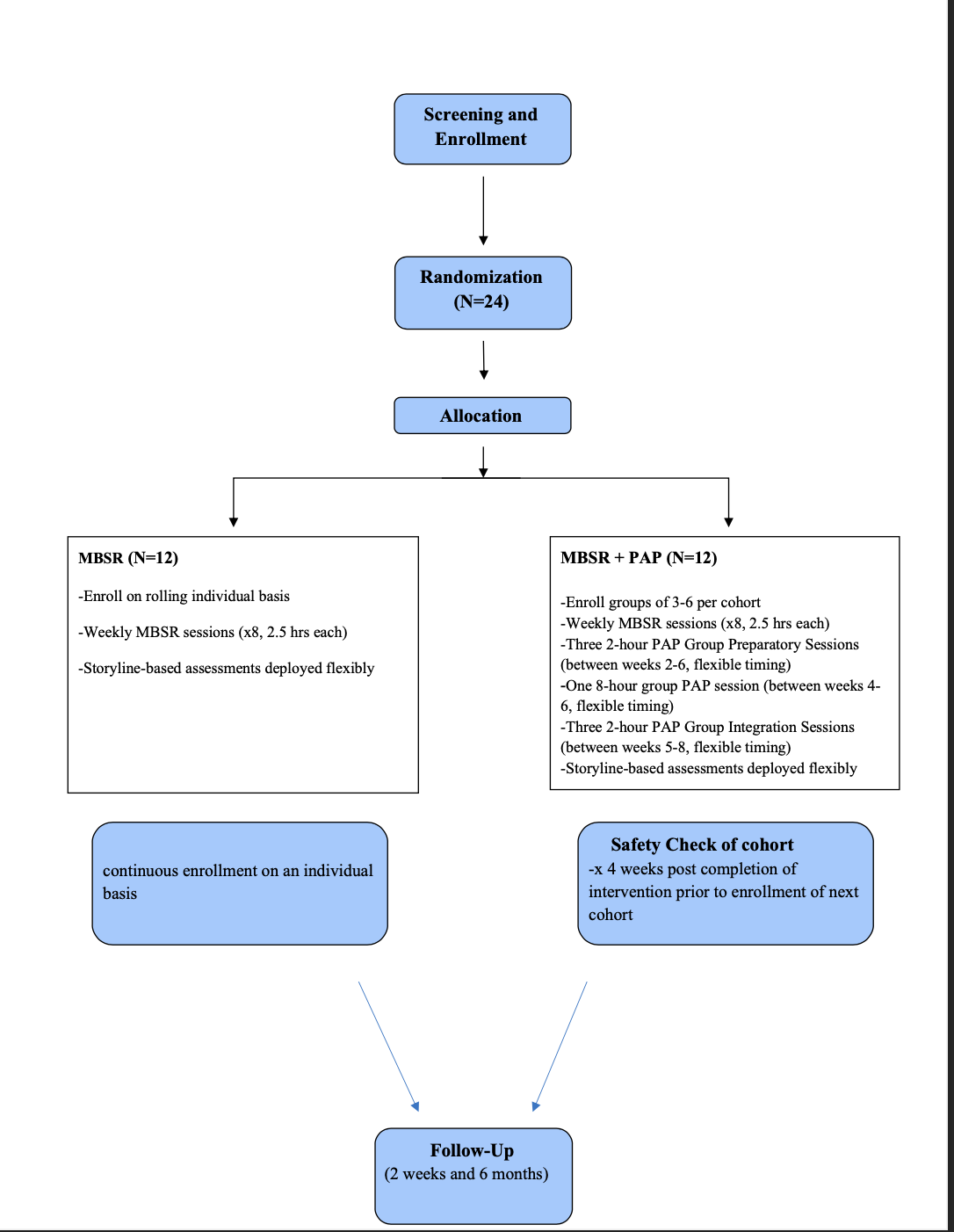


# OBJECTIVES

## Primary Objectives

### Determine the acceptability, feasibility, and safety of psilocybin-assisted therapy (PAP) plus Mindfulness-Based Stress Reduction (MBSR) for front-line healthcare worker burnout.

*Primary Endpoint:* Acceptability of each modality will be determined by ease of enrollment and retention. Feasibility will be measured by the number of enrollees who successfully complete each modality in both study arms. Safety will be measured through the monitoring of adverse events (AEs) utilizing the CTCAE (NCI Common Terminology Criteria for Adverse Events Version 5.0), suicidal ideation utilizing the Columbia Suicide Severity Rating Scale (C-SSRS), administration of the Challenging Experiences Questionnaire (ChEQ), direct participant qualitative reports, and clinician observation.

### Assess the preliminary efficacy of MBSR+PAP vs MBSR alone in reducing symptoms of depression (as measured by the QIDS-SR-16).

*Primary Endpoint:* Efficacy will be assessed through mean reduction in QIDS-SR-16 scores at 2-weeks post intervention with between-group comparison. To understand longitudinal effects, we will gather data to 6 months post intervention for both arms. Participants will be screened for eligibility with the PHQ-9 with a cut-off score of ≥ 10 required for enrollment and participants must meet DSM-5 criteria for a depressive disorder (including adjustment disorder with depressed mood).

*Primary Endpoint:* The change in depression scores as measured by the QIDS-SR-16 from enrollment to 26 weeks post-completion of the intervention (MBSR curriculum).

## Secondary Objectives

### To assess the effect of MBSR vs. MBSR+PAP has on the participant-reported symptoms of burnout (as measured by the MBI-HSS(MP)).

*Secondary Endpoint:* The change in mean MBI-HSS(MP) scores from baseline to 2 weeks post intervention with between-group comparison.

*Secondary Endpoint:* The change in mean MBI-HSS(MP) scores from baseline to 6 months post-completion of MBSR intervention.

### To assess the effect of MBSR vs. MBSR+PAP on symptoms of demoralization using the Demoralization II Scale (16 item)

*Secondary Endpoint:* The change in the Demoralization II Scale scores from baseline to two weeks post-completion of MBSR intervention.

### To assess the effect MBSR vs. MBSR +PAP has on symptoms of post-traumatic stress as assessed by the PCL-5 PTSD checklist

*Secondary Endpoint:* The change in PCL-5 scale scores from baseline to two weeks post-completion of MBSR intervention.

### To assess the effect MBSR vs. MBSR + PAP has on measures of connectedness to self, other, and world.

*Secondary Endpoint:* The change in score on the Watts’ Connectedness Scale (WCS) scale from baseline to two weeks post-­completion of the MBSR intervention.

## Exploratory Objectives

### To capture and explore behavioral data markers using the Storyline Health AI platform that may relate to other study outcomes and which may inform future research utilizing the Storyline Health Integrative Assessment tool.

We will be utilizing *Storyline Health* to track user questionnaires, experiences and other study data. This is a recently developed system that will allow us to track a variety of behavioral data points previously difficult to track including micro-expressions, pupillometry, voice tone, cadence and prosody, among other markers. Storyline Health is a platform that allows scalable, remote, and objective profiling of human cognition, expression, and behavior using a video-enabled smartphone. Storyline enables researchers to create clinical and behavioral assessments and care pathways, deploy them at scale to user smartphones and capture video responses that are securely stored in the cloud. The researcher then launches the Storyline AI pipeline, which extracts over 20,000 different measures of human facial, speech, and vocal patterns in each video, such as sub-second facial micro-expressions, facial blood flow patterns, word choice, sentiment, and vocal micro-tremors. The de-identified data is stored in storyARC and storyTIME files for download, analysis and sharing. Using Storyline, precise biomarkers and predictive models of illness, disease subtypes, drug responses and outcomes can be discovered, validated and then delivered instantly to providers and patients through **Storyline Telehealth**. The **Storyline Library** is an “App-like store” that makes proven assessments, models and care pathways available to providers. Therefore, Storyline seamlessly integrates clinical research with care delivery at scale, enabling rapid translation of biomarkers and algorithms.

*Tertiary Endpoint:*

- We will deploy the Storyline Integrative Assessment (see Appendix 1) to narrow in on specific feature selection in small cohorts of patients with deep AI phenotyping to identify behavioral phenotype predictors of burnout, which is heterogeneously characterized. This will be administered to both study arms at baseline, at the primary outcome point (2 weeks post intervention on both study arms), and at 6 months post intervention.

*Tertiary Endpoint:*

- We will deploy Storyline technology to narrow in on specific feature selection using deep AI phenotyping to identify phenotypical predictors of treatment response to both MBSR as well as MBSR+PAP to better characterize factors that predict response to both interventions, which may inform future research. This will be administered to both study arms at baseline, at the primary outcome point (2 weeks post intervention on both study arms), and at 6 months post intervention.

### To assess the effect qualitative experiential aspects of the psilocybin session have on subsequent therapeutic change.

*Tertiary Endpoints:* Patient-reported Nondual Awareness Dimensional Assessment (NADA), Mystical Experiences Questionnaire (MEQ-30).

Pearson correlation between total scores on the Mystical Experience Questionnaire (MEQ30) and NADA assessed at the end of session 1 and the effects on primary measures assessed 2 weeks after completion of the MBSR intervention.

### To assess the effect MBSR vs. MBSR + PAP have on measures of state and trait mindfulness as well as reappraisal.

*Tertiary Endpoints:* Trait mindfulness will be measured by the Five Facet Mindfulness Questionnaire (FFMQ), with comparison from baseline to two weeks post-completion of the intervention. State mindfulness will be measured by the Toronto Mindfulness Scale (TMS) with comparison from baseline to two-weeks post completion of the intervention. Reappraisal will be measured via the positive reappraisal subscale of the Cognitive Emotion Regulation Questionnaire (CERQ) with comparison from baseline to two weeks post completion of the MBSR intervention. To assess a patient-reported psilocybin experience on the day of medication administration and to assess the relationship between reported experience and symptoms of depression and burnout.

# BACKGROUND

Burnout is a recognized psychological syndrome with three dimensions: a) emotional exhaustion, b) depersonalization, and c) reduced personal accomplishment (Maslach, 2016). Healthcare provider burnout has been recognized as a worsening crisis in the U.S. medical system for many years. This has been accentuated with the onset of the SARS-CoV-2 pandemic where chronic, system-dependent stressors related to work dissatisfaction, lack of personal meaning, increasing administrative burden, and marginalization of providers have been coupled with sudden, dramatic increases in clinical demand, limited resources and resource rationing, assumption of increased personal risk, and increasing difficulties in balancing family life and multiple roles and responsibilities. Healthcare provider burnout has significant downstream effects on patient care due to its association with impaired job performance, decrease in empathy, and increases in medical errors (Panagioti et al., 2018). A 2019 analysis by Han et al. suggested that the healthcare costs attributable to physician burnout in the U.S. exceed $4.6 billion dollars per year (Han et al., 2019). Provider burnout is clearly linked to poor mental health outcomes among providers, increased problematic substance and alcohol use, as well as safety concerns with patient care (Shanafelt et al., 2011; West et al., 2012). Provider burnout is also correlated with reduced patient satisfaction scores (McKee et al., 2020). To date, there have been no firmly established meaningful interventions for this important psychological syndrome, however there is evidence to suggest that mindfulness-based interventions may lead to reductions in symptoms of burnout (Smigielski 2019).

There are compelling reasons to hypothesize that psilocybin-assisted psychotherapy (PAP) may offer a uniquely effective way of augmenting the benefits of mindfulness interventions as well as catalyzing significant improvements in symptoms of burnout. PAT has not been studied for healthcare provider burnout (however these investigators are aware of a study underway in Washington led by Anthony Back, MD).

This proposal is for a phase I, open-label, clinical pilot study to a) determine the acceptability, feasibility, and safety of PAP + an 8-week Mindfulness Based Stress Reduction (MBSR) curriculum, b) gather preliminary data as to the comparative efficacy of MBSR vs. MBSR + PAP in the management of depressive symptoms and burnout symptoms in healthcare providers, and c) inform future studies combining mindfulness-based interventions and psychedelic-assisted therapy interventions.

Mindfulness-Based Stress Reduction (MBSR) is a well-established evidence-based mindfulness training program initially developed by Jon Kabat-Zinn that has been shown to reduce symptoms of depression, anxiety, and burnout (Khoury 2015). The 8-week MBSR curriculum provides training in formal meditation practices including body scan techniques, mindfulness meditation, and yoga. Mindfulness can be understood as the non-judgmental acceptance and investigation of present experience, including body sensations, internal mental states, thoughts, emotions, impulses and memories, in order to reduce suffering or distress and to increase well-being (Kabat-Zin 2013).

Psilocybin is a classic psychedelic—understood mechanistically as a serotonin 2A agonist—functioning as a rapidly-acting antidepressant with sustained effect even after only single drug administrations in controlled settings. Several studies (Davis et al., 2020, Griffiths et al., 2016, Ross et al., 2016, Grob et al., 2013) have convincingly demonstrated robust therapeutic improvements for symptoms of depression and anxiety associated with a cancer diagnosis with a single high-dose psilocybin experience that is sustained at 6 months post treatment session. Furthermore, recently published data (Agin-Liebes et al., 2020) suggested that this response is maintained for a significant proportion of participants (60-80% of patients) for >5 years. Notably, psilocybin has also been studied for a range of other conditions including tobacco dependence (Johnson et al., 2017), OCD (Moreno 2006) and alcohol-use disorder (Bogenshutz et al., 2015, 2016), demonstrating large-magnitude effects after a single administration.

These data suggest that the therapeutic effects of psilocybin are less dependent on the specific classification of psychopathology but, rather, target underlying neurobiological commonalities across disorders. Namely, it is hypothesized that psilocybin ameliorates cognitive rigidity, which characterizes many kinds of psychopathology. As Carhart-Harris has argued (2014, 2018, 2019), classic psychedelics—through increasing whole brain entropy by downregulation of default-mode network activity—alter the precision weighting of over-weighted or pathological priors: the brain enters a temporary hyperplastic state that can leave lasting effects in previously rigidly held belief states characteristic of certain psychopathological conditions such as major depressive disorder. Maladaptive beliefs (e.g., “My work with patients just doesn’t matter – it’s just a drop in the bucket.”) may contribute to burnout. In this sense, psychedelic medicines and psychedelic-assisted psychotherapies seem poised to become the most important innovation in mental health care in several decades.

While interventions such as psilocybin-assisted psychotherapy do not address larger environmental contributions to burnout—conceptualized as a “profound lack of alignment between caregivers’ values and the reconfigured healthcare system” (Hartzband, 2020)—there are nonetheless good reasons to hypothesize that this modality may address risk factors at the individual level in a significant and sustained fashion—namely that PAP targets the intrinsic personality traits that have been shown to increase vulnerability to burnout: psychological rigidity, perfectionism, compulsiveness, and baseline neuroticism (Gazelle et al., 2015). Psilocybin has been shown to significantly alter intrinsic personality elements in a sustained fashion, specifically increasing the personality domain of openness which has been shown to be sustained at one year after single dose administration (Maclean et al., 2011). It has also been shown to enhance prosocial attitudes and behaviors and promote sustained positive changes in psychological functioning (Griffiths, 2018). The three-dimensional model of provider burnout clearly situates the individual stress experience within a social context and directly involves an individual’s conception of self and others (Maslach, 2012). If we conceive of self-related conscious experience as largely modulated by the activity of the default mode network, and certain dysfunctional cognitive and behavioral repertoires as being mediated by overactivity within this network, then psilocybin—a serotonin 2A agonist which directly reduces activity in the default mode network in a dose-dependent fashion—may offer a viable treatment for burnout, which is largely manifested in terms of dysfunction in self-experience, self-awareness, identity, and relationship between the self and others.

This project will be employing Storyline Health technology. Storyline Health is an AI platform that enables deep AI phenotyping of facial, speech and vocal patterns captured from asynchronous clinical interviews delivered over a smartphone. These innovations include the creation of technology for asynchronous clinical interviewing with simultaneous video capture, software for creating and delivering novel assessments, a military-grade data security solution for safely storing patient video data, an AI pipeline that extracts over 20,000 different measures from patient facial, speech and vocal patterns and architects them into easy to use, de-identified storyARC and storyTIME data file types that we created to enable research, discovery and model development. Storyline Health involves innovative technologies to deliver proven assessments, biobehavioral markers, care pathways and predictive models into clinical care settings at massive scale through the Storyline Library (an app-like store for AI health models) and Storyline telehealth. The clinical category of ‘burnout’ is heterogeneous in characterization and poorly understood. This limits both prediction of features of burnout among clinicians as well as meaningful interventions to address symptoms of burnout. We will be using the Storyline Integrative Assessment to build a model for diagnosing and tracking symptoms of burnout as well as predicting treatment response, particularly given how heterogeneously characterized this condition is at present. Similarly, the field of psychedelic-assisted therapy is in early stages: there are limited studies at present to inform questions as to treatment response and prediction of adverse outcomes. Storyline Health offers a unique and powerful way of informing many of these foundational questions for the field.

We have an ongoing relationship with Usona, a pharmaceutical company that manufactures psilocybin and which is providing the psilocybin for our HOPE clinical trial. Our research group is committed to the Statement on Open Science and Open Praxis with Psilocybin, MDMA, and Similar Substances: https://files.csp.org/open.pdf

## Psilocybin

Psilocybin, considered a ‘classic psychedelic,’ is a naturally occurring as well as a synthesized psychedelic prodrug that is the active compound found in ‘magic mushrooms.’ Once ingested orally psilocybin is rapidly metabolized to psilocin which acts as a serotonin-2A agonist in the brain, producing alterations in consciousness that typically last between four and six hours and which are thought to be largely related to down-regulation in the activity of high-level association cortices included within the default mode network (DMN). These alterations in conscious experience include a wide range of subjective effects including profound alterations in emotional tone, distortions in time perception, visual perceptual changes, sense of introspective insight, and experiences of spiritual or mystical connection. Interestingly, the intensity of the mystical experience is the best predictor of the magnitude of therapeutic effect post psilocybin administration, both in the immediate aftermath as well as measured longitudinally. This is found across studies on psychedelic-assisted therapy across diagnostic categories from existential distress and associated depressive and anxiety symptoms, to smoking cessation, to the maintenance of sobriety with alcohol use disorder. Mystical experience can be understood and measured along with indices such as a sense of unity (or loss of ego/sense of self), transcendence of time and space, sense of ineffability, sense of sacredness, noetic quality (or sense that a deep intuitively self-evident truth is being revealed), and positive mood- as measured with the States of Consciousness Questionnaire (SOCQ) or Mystical Experience Questionnaire (MEQ30). As such, the emphasis in psychedelic-assisted psychotherapy interventions is on the particular subjective or phenomenological elements of the experience, which is particularly unique in psychopharmacology: “the idea that a single discrete experience can result in lasting beneficial effects in an individual’s attitudes or behavior is highly unusual, if not unprecedented within the modern biomedical paradigm” (Garcia-Romeu, Griffiths, Johnson, 2015).

This dose of psilocybin (25mg) is the same dose used in the modern studies conducted by the Imperial College of London (Carhart-Harris, 2016; 2017). The Johns Hopkins group has used weight-based dosing strategies (22mg/70kg in Griffiths, 2016); however, there is insufficient data at this point—beyond a certain dose threshold—that this strategy is necessary. 25mg of psilocybin represents a high dose as the magnitude of mystical-type experience is a dose-related phenomenon.

Psilocybin is a classic psychedelic of medium duration that is well-tolerated and has a documented safety and efficacy record that makes it uniquely well-suited to the existential issues that arise in this patient population. Psilocybin has been described as an ‘existential medicine’, given patient testimonials as to its acute and lasting effects on interpersonal connection, ability to more deeply engage with meaningful activities and relationships, dramatic reductions in fear of death, and a renewed sense of well-being.

While psilocybin can cause dramatic and potentially disorienting alterations to consciousness and individual psychology, it is remarkably safe with an estimated LD50 that is 1,000 times greater than the effective dose. Common physical reactions can include pupillary dilation, tachycardia, slight elevations in blood pressure, nausea, and tremor. There are examples to be found as to destabilizing or harm-inducing psychological experiences given the diversity of settings in which this compound is used; however, within the context of controlled psychotherapeutic administration there have been no adverse reactions of this kind within the recent and existing literature. While psilocybin—along with the other ‘classic psychedelics’—is currently on Schedule I, it has been ranked as the illicit drug with the lowest risk of harm (Nutt, 2010) and minimal abuse potential given the rapid development of tolerance, minimal reinforcement learning, independence from dopaminergic circuits implicated in drugs of abuse, and empirical track record.

The best current models of explaining the therapeutic effects of psilocybin for symptoms of depression and anxiety describe network-level effects that psilocybin has on the brain both acutely as well as longitudinally, in particular down-regulation of brain activity in high-level association cortices associated with the default mode network (DMN). The DMN is a highly interconnected set of cortical brain regions (posterior cingulate cortex, medial prefrontal cortex, and angular gyrus) that is highly metabolically active at rest and is responsible for higher-level metacognitive processes such as mental time travel, self-reflection, the distinction between self and other, mind wandering or rumination, and autobiographical thinking. Hyperactivity in the DMN is implicated in a range of psychopathological conditions. Psilocybin, along with other 5HT-2A agonists, stimulates receptors in the DMN (which is highly dense in 5HT-2A receptors), dysregulating organized neurotransmission within this circuit. These effects are responsible for the transient acute effects of this psychedelic compound but are also seen to be related to the ongoing therapeutic benefits that can persist even after a single administration given enhanced neuroplasticity following treatment and increased openness to revisiting previously held—and possibly pathological—belief states (often characterized in depression as excessively negative ruminations on the self).

### Clinical Experience

The study team will be comprised predominantly of MD physicians, psychologists, and social workers who are trained in psychedelic-assisted psychotherapy. Benjamin Lewis MD and John Hendrick MD will serve as lead study monitors and at least one of these individuals will be present for all psilocybin dosing sessions along with the therapist team (in 1:1 therapist to participant ratio). Benjamin Lewis MD or John Hendrick MD will perform the final safety, medical, and psychiatric check prior to participants leaving the site on the psilocybin dosing day. Kelly Lundberg PhD (Clinical Psychology) will serve as the Lead Study Therapist. Dr. Lundberg will be present for all preparation sessions, the psilocybin session, and the integration sessions for the MBSR+PAP arm. All individuals involved in the therapeutic work with patients have completed a two-day training led by Alan Davis Ph.D., Mary Cosimano MSW, and Rafael Lancelotta MS, held in February 2020 as well as a 1-day additional training session with Mary Cosimano MSW. This study builds upon work in psychedelic-assisted therapies conducted by our research group. We have completed a 12-subject open-label safety and feasibility pilot study of psilocybin-assisted group therapy for patients with depression associated with a cancer diagnosis: “HOPE: A pilot study of psilocybin enhanced group psychotherapy in patients with cancer” (IRB_00131965, PI Anna Beck MD). We are also currently engaged in a randomized controlled trial (RCT) investigating ketamine-assisted therapy in conjunction with a group mindfulness curriculum for patients with opioid use disorder: “Mindfulness Oriented Recovery Enhancement (MORE) with Ketamine Assisted Psychotherapy (KAP) for the treatment of opioid use disorder” (IRB_00130630, PI Eric Garland PhD). Dr. Lewis is a site investigator for the COMPASS Phase III trial of psilocybin assisted therapy for treatment resistant depression. Our group is also involved in gathering survey-based data on psychedelic use to inform and guide future research questions (see IRB_00129303 (PI Kevin Byrne MD). Ben Lewis has completed a year-long training program in psychedelic therapy and research through the California Institute of Integral Studies, is certified by MAPS in MDMA-assisted psychotherapy, and also completed a three-day training in psychedelic-assisted therapy through the Psychedelic Research & Training Institute group (PRATI). Dr. Lewis also serves on the State of Utah Psychotherapy Drug Task Force determining parameters and recommendations for medical use of psychedelic-assisted therapies as well as the National Network of Depression Centers (NNDC) workgroup for psychedelic medicine.

## Design Rationale

While interventions such as psilocybin-assisted psychotherapy do not address larger environmental contributions to burnout, conceptualized as a ‘profound lack of alignment between caregivers’ values and the reconfigured healthcare system’ (Hartzband, 2020), there are nonetheless good reasons to hypothesize that both PAP and MBSR individual as modalities may address risk factors at the individual level in a significant and sustained fashion—namely by targeting the intrinsic personality traits that have been shown to increase vulnerability to burnout: psychological rigidity, perfectionism, compulsiveness, and baseline neuroticism (Gazelle et al., 2015). Psilocybin has been shown to significantly alter intrinsic personality elements in a sustained fashion, specifically increasing the personality domain of openness which has been shown to be sustained at one year after single dose administration (Maclean et al., 2011). It has also been shown to enhance prosocial attitudes and behaviors and promote sustained positive changes in psychological functioning (Griffiths, 2018). The three-dimensional model of provider burnout clearly situates the individual stress experience within a social context and directly involves an individual’s conception of self and others (Maslach, 2012). If we conceive of self-related conscious experience as largely modulated by the activity of the default mode network, and certain dysfunctional cognitive and behavioral repertoires as being mediated by overactivity within this network, then psilocybin—a serotonin 2A agonist which directly reduces activity in the default mode network in a dose-dependent fashion—may offer a viable treatment for burnout, which is largely manifested in terms of dysfunction in self-experience, self-awareness, identity, and relationship between the self and others.

There are compelling theoretical reasons to hypothesize that a single group-format psilocybin session may augment gains observed with mindfulness interventions. Franz Vollenweider’s research group completed a double-blind study of experienced meditators with half of the participants taking part in a guided psilocybin-session as part of a five-day mindfulness group retreat (Smigielski et al., 2020). Compared with placebo, psilocybin enhanced post-intervention mindfulness and produced larger positive changes in psychosocial functioning at the 4-month follow up, which were mediated by measures of reported self-dissolution.

Current studies of psychedelic-assisted therapy are limited by several factors, including limited control groups and limited studies with active comparison groups. While early results from several smaller trials are remarkably positive, there remain questions as to study design and real-world feasibility. This study will gather preliminary efficacy data on MBSR vs. MBSR + PAP to inform larger future studies that will be better powered to detect efficacy differences between these two arms and better characterize how these two treatment modalities might be used together.

An innovative element of this study design is the use of group-format psilocybin-assisted therapy sessions. This is similar to the model employed in the HOPE trial which demonstrated safety and feasibility (results not yet published). A majority of trials to date have employed individually-based sessions, including all preparatory and integration work, with a 2:1 therapist-to-participant ratio. This conservative model presents challenges in terms of scalability and feasibility. The use of psychedelic compounds in group settings for religious/spiritual purposes dates back thousands of years. Group format interventions may offer distinct therapeutic advantages for certain patient populations and indications, particularly given the capacities of classic psychedelics to engender feelings of interpersonal connectedness, pro-sociality, and empathy (Pokorny 2017). As such there are compelling reasons to hypothesize that these compounds can catalyze therapeutic effects of group therapeutic process, including a group-based mindfulness process. Similarly, group-based interventions stand to augment and amplify the clinical gains induced by the psilocybin experience, particularly during the integration phase of the interventions. These reciprocally synergistic effects may promote positive clinical outcomes as well as offer a more practical and accessible model of care delivery.

Another innovative element of this study involves the use of *Storyline Health* which is a secure, massively scalable, smart-phone-based platform for AI enabled deep behavioral profiling ([www.storylinehealth.com](http://www.storylinehealth.com)). Storyline enables researchers to design assessments and then send them at scale to study participants for completion on a video-enabled smartphone or other device. The platform securely manages messaging between the researcher and participants. The participant uses the Storyline mobile app (Apple store and Google Play) to create and manage their behavioral health record and perform study assessments on their smartphone or computer. Mental and behavioral health information is sensitive, carries a stigma and can be embarrassing to report honestly. For this reason, Storyline exceeds HIPAA, GDPR and CCPA requirements. Participant responses are removed from their vulnerable devices and stored in the cloud using military-grade security. Participant videos are only accessible to approved study researchers and participants retain access and control over their data.

Storyline provides an AI pipeline for deep phenotyping of human facial, speech, and vocal patterns. Researchers select study participant videos for analysis and clicking the “storyARC” button in the “Analytics” section of the user dashboard This activates the Storyline AI-analysis pipeline to extract +20,000 different measures per person of facial, speech, and vocal data for each question. The data are scrubbed for identifiable information. De-identified data are compiled to a “storyARC” data file for analysis and statistical modeling. AI analysis involves the following steps:

**Facial Pattern Analysis:** Based on real-world learning the Storyline facial analysis pipeline is designed to be the leading solution to analyze facial data in video. It uses a custom pipeline to measure features that are not captured by commercial platforms, and are critical for biobehavioral marker discovery. Output is compared to commercial AI platforms (e.g., IBM Watson, Amazon Rekognition, Microsoft Azure) to ensure industry leading accuracy.

**Speech Pattern Analysis:** The Storyline speech analysis pipeline is also a multi-step, micro-services pipeline. The output is tested using 10 leading commercial and open source tools (e.g.,AWS Comprehend Medical, Twinword, IBM Watson Tone Analyzer, IBM Watson Personality Analyzer, Google Text Analysis).

**Vocal Pattern Analysis:** The Storyline vocal analysis pipeline includes the industry leading OpenSMILE platform and incorporates features extracted using other leading Python-based tools for vocal pattern analysis, including Praat and ProsodyPro.

We will be deploying the Storyline Integrative Assessment which incorporates neurological, psychiatric, cognitive, and psychological assessments. This data stands to deeply inform both the recognition and treatment of burnout as well as the nascent science on psychedelic-assisted therapies.

# DRUG INFORMATION

## Psilocybin

### Pharmacology

Psilocybin is a natural product produced by numerous species of *Psilocybe* mushrooms. It is synthesized de novo and manufactured for clinical use to control potency and purity. The Controlled Substances Act currently classifies psilocybin as a Schedule I drug.

### Mechanism of Action

After absorption, psilocybin is enzymatically cleaved to produce psilocin, which acts as an agonist of serotonin receptors, including 5-HT2A. 5-HT2A receptor stimulation depolarizes layer 5 pyramidal neurons leading to an increased firing rate. This increased firing in prefrontal cortex results in increased glutamatergic recurrent network activity.

### Pharmacokinetics

Following oral administration (0.224 mg/kg) of psilocybin, average blood concentration of the active metabolite psilocin was calculated to be 8.2 ± 2.8 ng/mL after 105 ± 37 minutes, yielding an estimated dose-normalized bioavailability of psilocybin to be 52.7 ± 20% (N = 3). Psilocin typically appears in plasma within 15 minutes after oral administration. Psilocin half-life following oral administration of psilocybin was found to be approximately 3 ± 1.1 hours, and is detectable for up to 24 hours after administration. The levels of psilocin peaked at approximately 80 minutes; however, rates can vary between individuals.

Psilocin is metabolized to 4-hydroxyindole-3-acetic acid by deamination and demethylation via liver enzymes such as monoamine oxidase, and aldehyde. Psilocin is also extensively glucuronidated by the UDP-glucuronosyltransferase (UGT) family of enzymes, with the highest glucuronidation activity demonstrated by UGT1A10. The amount of psilocin glucuronide excreted renally has been shown to exceed that of psilocin over a 24-hr time period, and analysis of psilocin in urine over 24 hours after a single dose has shown that less than 4% of the overall clearance of psilocin occurs through renal excretion. The pharmacokinetics of psilocybin (as psilocin) are linear over the dose range of 0.3 – 0.6 mg/kg.

### Physical and Chemical Properties

Psilocybin is a tryptamine derivative presenting as a white crystalline solid with a melting point of 220-228°C. It is stable over extended periods in dark storage at controlled room temperature. Psilocybin is soluble in 20 parts boiling water or 120 parts boiling methanol.

[3-[2-(dimethylamino)ethyl]-1H-indol-4-yl] dihydrogen phosphate


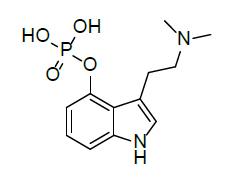


**Figure 1.** Psilocybin molecular structure

Chemical Formula: C_12_H_17_N_2_O_4_P

Molecular Weight: 284.3

### Clinical Safety

The clinical safety of psilocybin has been extensively studied, both as a single agent and as adjunctive treatment in adult populations. Psilocybin is administered orally, and has been studied in open-label, and double-blind, controlled trials. Dosing regimens have ranged from 0.014 mg/kg to 0.6 mg/kg, administered as either a single dose, or multiple doses weeks apart.

The most common adverse experiences are psychological, including anxiety, and the induction of negative emotional states and paranoid/delusional thinking during psilocybin sessions. The most common physical adverse events are cardiovascular (increased blood pressure and heart rate), as well as nausea and headache.

A meta-analysis of eight double-blind, placebo-controlled studies including 110 healthy subjects who had received 1–4 oral doses of psilocybin (45–315 μg /kg body weight) showed that effects of psilocybin are dose-dependent, although other factors such as personality structure and the setting (e.g., environment) appear to modulate its overall effects. Although psilocybin dose-dependently induced profound changes in mood, perception, thought, and self-experience, most subjects described the experience as pleasurable, enriching, and non-threatening. Acute adverse drug reactions, characterized by strong dysphoria and/or anxiety/panic, occurred transiently only in the two highest dose conditions in a relatively small proportion of subjects (5 and 8% respectively). All acute adverse drug reactions were successfully managed by providing interpersonal support and did not require psychopharmacological intervention.

# STUDY DESIGN

## Description

Recruitment will target physicians or nurses with at least 1 month of frontline clinical experience during the COVID pandemic who meet DSM-5 criteria for a depressive disorder, including adjustment disorder with depressed mood.

Participants that meet eligibility criteria and have a screening MBI-HSS (MP) score that meets our working definition of burnout (emotional exhaustion subscale ≥ 27) and a ‘high’ score on one other subscale (either depersonalization ≥ 13 or personal accomplishment ≤ 21) as well as a PHQ-9 depression screen ≥ 10 will be offered enrollment and following a consenting process will be randomized to either MBSR or MBSR + PAP with 12 participants per group. PHQ-9 and MBI-HSS questionnaires may be administered prior to consenting to assess eligibility. Consenting will be completed in person with paper or electronic consenting forms. The MBSR group will complete an 8-week MBSR curriculum (online) through the University of Utah Resiliency Center led by Trinh Mae, LCSW which includes a weekly 2.5-hour sessions over Zoom for 8 weeks and an 8 hour in-person retreat between weeks 6 and 7. If capacity within the University of Utah MBSR groups limits enrollment and feasibility we will incorporate the MBSR programs through Intermountain as well as Davis Behavioral Health. We will run subjects through the MBSR curriculum in flexible numbers concurrently until a total of 12 have completed this arm. The MBSR + PAP group will complete this same curriculum, however will substitute an 8-hour group-format psilocybin-assisted therapy session (with 25 mg psilocybin capsule) with 1:1 therapist to participant ratio instead of the MBSR retreat day. Each participant will have an assigned individual therapist present for the duration of the psilocybin dosing session. A lead therapist (Kelly Lundberg PhD) will also be present for all study sessions including the psilocybin dosing day and there will be at least one lead study monitor present for the duration of the psilocybin dosing day (Benjamin Lewis MD or John Hendrick MD). The psilocybin intervention will be completed in groups of 2-6 individuals per cohort. Additionally, the MBSR + PAP group will have 3, 90-minute group preparatory sessions prior to the psilocybin session and 3 subsequent 90-minute integration sessions. The first integration session will occur 1-2 days following the psilocybin session. A communal music track will be played during the PAP session. After the enrollment and treatment of the first four patients, accrual will be placed on hold to ensure participant safety. If stopping rules are not met, we will continue with enrollment.

PAP Preparation sessions will focus on psychoeducation regarding the effects of psilocybin, guidance as to navigating the experience, establishing safety and trust with the therapist team, and specific mindfulness focused skills (drawing on the MBSR curriculum) that can be employed during the experience. The psilocybin session will employ a 1:1 therapist to participant ratio which is the same model as the HOPE trial run by this team. These sessions will also be video recorded with participant consent. This session will be conducted in a largely non-directive and supportive fashion similar to the current therapy model used in psychedelic-assisted therapy research. However, we will book-end the dosing day with specific mindfulness exercises drawing from the MBSR curriculum. At the end of this 8-hour session all participants will be assessed for safety to leave the premises including a screen for suicidality, a final set of vital signs and medical evaluation, as well as a cognitive assessment as to orientation. Rides and support person will be confirmed by the study team. The first integration session will occur within 48 hours of this dosing day however the therapist team will be available by phone throughout this time in case there is need for additional support. Subsequent integration sessions (2 additional) will be integrated into the MBSR curriculum, following the weekly group MBSR session. These sessions will focus on processing the experience and relevant emotional and psychological material as a group as well as integrating this with perspectives and insights gained through the mindfulness curriculum.

## Number of Patients

This study will enroll a total of 24 patients with 12 patients in each study arm.

## Number of Study Centers

This will be a single-center trial run at the University of Utah Hospital.

## Study Duration

Study therapy will be a total of 9 weeks duration (with 8-week MBSR curriculum) and patients will be followed for 6 months (180 days) after completion of the study intervention. There will be a total of two groups of 12 subjects per study arm. Total duration for the study will be approximately one year.

# ELIGIBILITY CRITERIA

Potential study participants must meet all inclusion criteria and no exclusion criteria to be deemed eligible for trial participation. To ensure subject safety, all patients must be deemed eligible at the time of study registration and must continue to meet eligibility criteria up to the first group therapy session. This eligibility checklist is used to determine subject eligibility and will be filed with the enrolling investigator’s signature in the subject research chart.

**Subject No. ______________________**

**Subject’s Initials: (L,F,M) _________________**

## Inclusion Criteria

___ Participants must be physicians or nurses with at least 1 month of frontline clinical experience during the COVID pandemic.

**Yes/No Eligibility Questions (Response of “no” = subject ineligible)**

- PHQ-9 score ≥ 10 and meet DSM-5 criteria for a depressive disorder, including adjustment disorder with depressed mood.
- Meet our working definition of burnout which will involve a score on the emotional exhaustion subscale (≥ 27) and a ‘high’ score on one other subscale (either depersonalization ≥ 13 or personal accomplishment ≤ 21).
- Not taking regularly scheduled medications to treat depression and/or anxiety, including benzodiazepines, for at least 4 weeks prior to administration of psilocybin, if randomized to that study arm.
- Fluent in English.
- Reading literacy and comprehension sufficient for understanding the consent form and study questionnaires, as evaluated by study staff obtaining consent.
- Able to provide informed consent and willing to sign an approved consent form that conforms to federal and institutional guidelines.
- ECOG Performance Status ≤ 2.
- Have a support person that would be able to escort the subject home on the evening of the psilocybin dosing session. The use of ride services will not be permitted (e.g., Uber, Lift, taxi, etc.)
- (If randomized to psilocybin arm) Adequate liver function as defined as:
  - Total Bilirubin < 1.5x institutional upper limit of normal (ULN) unless elevated bilirubin is related to Gilbert’s Syndrome.
  - AST(SGOT)/ALT(SGPT) <3 x institutional ULN
  - For female subjects: Negative pregnancy test and agreement to use highly effective contraception or evidence of post-menopausal status if randomized to psilocybin arm. The post-menopausal status will be defined as having been amenorrheic for 12 months without an alternative medical cause.
  - For male subjects: agree to condom use during intercourse for 24 hours post-psilocybin dose.
  - Agree to refrain from using any psychoactive drugs, including alcoholic beverages, ondansetron, cannabis, and non-routine PRN medications within 24 hours of the psilocybin administration. Exceptions include daily use of caffeine, nicotine, and opioid pain medication.
  - Agree that for one week preceding the psilocybin session, he/she will refrain from taking any nonprescription medication, nutritional supplement, or herbal supplement except when approved by the research team. Exceptions will be evaluated by the research team and will include acetaminophen, non-steroidal anti-inflammatory drugs, and common doses of vitamins and minerals.
  - Agree not to use nicotine for at least 2 hours before the psilocybin administration and for the duration of the psilocybin session.
  - Agree to consume approximately the same amount of caffeine-containing beverage (e.g., coffee, tea) that he/she consumes on a usual morning, before arriving at the research unit on the morning of the psilocybin session. If the subject does not routinely consume caffeinated beverages, he or she must agree not to do so on the day of psilocybin administration.
  - Subjects requiring opioid use for pain are on a stable pain management regimen. Long-acting opioid medications (e.g., oxycodone sustained-release, morphine sustained release) will be allowed if the last dose occurred at least 6 hours before psilocybin administration; such medication will not be taken again until at least 6 hours after psilocybin administration.

## Exclusion Criteria

**Yes/No Eligibility Questions (Response of “yes” = subject ineligible)**

- Prior systemic antidepressants, antipsychotic, or anxiolytic medication within four weeks prior to administration of psilocybin (if randomized to psilocybin arm).
- Personal history or first- or second-degree relatives with schizophrenia, bipolar affective disorder, delusional disorder, schizoaffective disorder, psychosis, or other psychotic spectrum illness as determined by patient report and chart review.
- Current or history within the last two years of meeting DSM-V criteria of substance use disorder (excluding caffeine and nicotine). Current substance use disorders may be identified through the drug urine screening test as determined by patient report and chart review.
- Currently meeting DSM-V criteria for Dissociative Disorder, or other psychiatric conditions judged to be incompatible with the establishment of rapport or safe exposure to psilocybin as determined by patient report and chart review.
- Currently meeting DSM-V criteria for Cluster B Personality Disorder as determined by patient report and chart review.
- Severe depression requiring immediate standard-of-care treatment (e.g., hospitalization).
- Suicidal ideation over the past month as assessed as a yes to question 3, 4, or 5 on the Columbia-Suicide Severity Rating Scale, Suicidal Ideation section
- Cancer with known CNS involvement, previously treated brain metastasis, or other major CNS disease.
- Employment as house staff/residents
- The subject has uncontrolled significant intercurrent or recent illness including, but not limited to, the following conditions:
- Cardiovascular disorders: Congestive heart failure, including all New York Heart Association Classes.
- Angina pectoris, cardiac hypertrophy, cardiac ischemia, myocardial infarction
- Uncontrolled hypertension at the time of enrollment (BP>140 systolic or 90 diastolic), coronary artery disease, artificial heart valve
- Prolonged or congenital long QT syndrome (>450 ms), serious cardiac arrhythmias, tachycardia, a clinically significant screening ECG abnormality
- Renal insufficiency as defined as creatinine clearance < 40 mL/min calculated by Cockcroft-Gault formula
- Hepatic disorders: Active infection including hepatitis B (known positive HBV surface antigen (HBsAg) result) or hepatitis C.
- Any other condition that would, in the investigator’s judgment, contraindicate the subject’s participation in the clinical study due to safety concerns or compliance with clinical study procedures (e.g., infection/inflammation, intestinal obstruction, unable to swallow medication, [patients may not receive the drug through a feeding tube], social/ psychological issues, etc.)
- Known prior severe hypersensitivity to investigational product or any component in its formulations (NCI CTCAE v5.0 Grade > 3).
- Subjects taking prohibited medications. A washout period of prohibited medications for a period of at least five half-lives should occur prior to psilocybin administration.

**I certify that this subject meets all inclusion and exclusion criteria for enrollment in this study.**

**______________________________ ____________ ___________**

**Investigator Signature Date Time**

## Recruitment Strategies

Potential participants will be recruited from within the University of Utah Healthcare System through internal advertisements, flyers, and through the University of Utah Resiliency Center. If recruitment through internal sources proves challenging, we will expand our efforts to a 25-mile radius from University of Utah Hospitals and Clinics.

# TREATMENT PLAN

## Treatment Schedule

### MBSR Arm

Participants randomized to the MBSR only arm will be enrolled in the MBSR curriculum led by Trinh Mai LCSW through the University of Utah Resiliency Center. Cost for University of Utah employees ($99) will be covered through the study. If capacity limits the feasibility of running this internally we will consider utilizing MBSR groups through Intermountain as well as Davis Behavioral Health and this cost ($250 per participant) will be covered through the study. Mindfulness-Based Stress Reduction, (MBSR), was developed by Jon Kabat-Zinn at the Center for Mindfulness, University of Massachusetts Medical School. MBSR is an intensive training in developing mindful awareness and accessing our innate capacity for health, healing and growth. Groups meet for an orientation, 9 weekly classes and an all-day retreat held in the final two weeks of the curriculum Guided instruction in various practices is provided, including sitting and walking meditation, body scan, gentle yoga, and mindful communication. These practices are enhanced through inquiry exercises, group dialogue, and daily home assignments.

MBSR has been shown to be an effective ameliorative measure for healthcare related burnout (Cohen-Katz, 2005) independent of PAP. Given this, we believe it will be an ideal active comparator to the novel administration of psilocybin in this population.

### MBSR+PAP Arm

Participants randomized to the MBSR + PAP arm will participate in the same curriculum as described above, however, will substitute a group PAP session instead of the group MBSR retreat. The PAP intervention will be facilitated with a 1:1 therapist/participant ratio with an additional lead therapist. Group size will be determined by ease of recruitment but will be no fewer than 2, and no more than 6 participants per PAP session. The group will be overseen by a lead therapist who will be available through the entirety of the dosing session and will not be assigned to any one individual participant. Timing of the PAP session will be during the MBSR curriculum with a goal of scheduling at same time as the MBSR retreat for the other arm. However, scheduling will be flexible depending on participant schedules, particularly given working demands of healthcare providers amid a pandemic: this session may occur before or after the group meditation retreat +/- 1 week. The psilocybin session will be accompanied by 3 preceding group preparatory sessions of 90 minutes each (one of these can be via Zoom per participant) as well as 3 integration sessions of 90 minutes each (up to two of these can be via Zoom per participant). Participants must continue to meet all inclusion criteria as well as hepatic and renal function requirements laid out in inclusion and exclusion criteria to receive psilocybin.

#### Preparatory Sessions

All Preparatory Sessions are group based and approximately 90 minutes duration. They will cover the following topics: establishing safety and boundaries, an introduction of set/setting, description of psilocybin effects, rules of conduct during psilocybin session, and how to handle strong emotions. Up to 1 one of these preparatory sessions can be done remotely via Zoom per participant. Preparatory materials will be buttressed with education materials provided via the Storyline platform.

General supportive-expressive group goals will be followed, including a focus on building rapport and trust with the therapist team, expressing emotions, exploring work related stressors and symptoms of burnout and depression, exploring traumatic work-related events or issues, examining life priorities, and utilizing the support of family and friends.

#### The Psilocybin Session

The psilocybin medication session will occur in a group space in HMHI. This space will have accessibility to a restroom, a private break-out room (to be used as needed), and the HMHI pharmacy. Code Blue response is available within the facility. There will be a 1:1 ratio of therapists to participants present as well as a lead therapist not specifically assigned to any one participant. The room will have a chair for the individual therapists to sit near the subject to offer support and guidance. Participants will be seated in individual “stations” but will be within visual and auditory reach of those in their cohort. There will be a more private space available if the lead therapist and individual therapist deem an individual as needing it, mostly in a scenario in which loud speaking, crying or other behavior becomes disruptive to the group. Video recording equipment will be present throughout the duration of the session and will be part of the consenting process. Participants will be given specific reminders regarding the process for using the restroom or having a light snack. Participants will remain on-site with their therapist team for eight hours after administration of psilocybin and will have arranged transportation from the study site at the end of the day.

Prior to leaving the site at the end of the psilocybin dosing day each participant will be assessed by the lead study monitor (Benjamin Lewis MD or John Hendrick MD) regarding safety to leave. This evaluation will consist of a medical screen for any concerning symptoms, a psychiatric screen for acute suicidality or significant emotional distress, and a cognitive screen assessing orientation. If there are clinical concerns that a given participant is not safe to leave the site along medical, psychiatric, or cognitive assessments the lead study monitor (one of the PIs) and the Lead Therapist will remain on site for ongoing assessment and support until the participant is deemed safe to depart with a designated ride and support person. If a given participant remains unsafe to leave the site despite ongoing assessment and support they will be escorted to either the HMHI Receiving Center (for psychiatric assessment and care) or the University of Utah Emergency Department depending on either psychiatric or medical clinical presentation.

#### Integration Sessions

Three group integration sessions will occur after the Psilocybin Session with the first occurring within 48 hours of the Psilocybin Session. Integration sessions will be 90 minutes in duration and participants may join up to two of these sessions remotely via Zoom at the clinical judgement of the study therapists and study team if there are scheduling difficulties. This material will also be buttressed by specific integration exercises through the Storyline platform. Focus of these sessions includes processing material that arose during the psilocybin session, examining emotional responses to this material, review of significance of this material in light of symptoms of depression and burnout, reflection on how to integrate this material into the workplace and personal life following the psilocybin session, practices to continue integrating this experience moving forward.

## Psilocybin

### Methods of Supply, Storage, Packaging, and Labeling

Usona Institute will provide psilocybin as 25mg capsules that will be packaged individually into high-density polyethylene bottles (30 cc). Bottles will be maintained at room temperature in a locked, secure location within the Investigational Drug Services Pharmacy at Huntsman Cancer Institute and in accordance with the Drug Enforcement Agency (DEA) regulations. Study staff with access to the psilocybin inventory will be pre-defined.

Once IP is prepared for dispense by the IDS pharmacy, the assigned treatment will be picked up from the IDS pharmacy by a study team member.  The individual who is picking up must be part of the research team and holding a Schedule 1 DEA license. No other individuals will be allowed to pick-up and transport the study drug to the study site. A chain of custody form and a medication pick-up log will be completed by both an IDS staff member and the research team member picking up the drug.

### Preparation and Administration

The Investigational Drug Services pharmacy at Huntsman Cancer Institute will prepare and dispense all investigational supplies. A designated study team member with a Schedule 1 DEA license will be responsible for transporting the study drug to the study site at HMHI. Appropriate records will be kept to accurately show all dispensing activates including the return of any unused Investigational Product (IP). IP will be supplied only to subjects deemed eligible for study therapy. IP may not be dispensed to patients who have not been enrolled in the trial.

Patients will be advised to refrain from the following for 24 hours before psilocybin:

- Cannabis
- Alcoholic beverages
- Psychoactive drugs
- Ondansetron
- Non-routine PRN medications

On the day of psilocybin administration, patients on opioids should not take any long-acting opioids within 6 hours prior to or 6 hours proceeding psilocybin administration. Patients should consume approximately the same amount of caffeine-containing beverage (e.g., coffee, tea) that he/she consumes on a usual morning, before arriving at the research unit. If the subject does not routinely consume caffeinated beverages, he or she must agree not to do so on the day of psilocybin administration. Patients should also not use nicotine for at least 2 hours before psilocybin administration.

All doses of psilocybin will be administered at the investigational site by qualified medical staff. The time of administration will be recorded in the patients’ research charts. Any reason for deviation from the protocol-specified dose or schedule will be documented in the subject’s research chart.

### Accountability and Compliance

The Investigational Drug Services Pharmacy at Huntsman Cancer Institute and the HMHI Pharmacy will maintain appropriate documentation of the receipt, dispensing, return, transportation, and destruction of all investigational product. All inventory tracking will be completed in duplicate by IDS pharmacy staff. A log will be maintained of all individuals with access to the IP and its storage location in a securely locked safe. Any theft or significant loss of IP will be reported to the DEA on DEA Form 106 within one business day of first knowledge in accordance with 21 CRF Part 1301.

Study drug will be properly stored in accordance with Drug Enforcement Agency requirements in the Investigational Drug Service Pharmacy and administered to registered study participants at Hunstman Mental Health Institute.

Once IP is prepared for dispense by the IDS pharmacy, the assigned treatment will be picked up from the IDS pharmacy by a study team member with a DEA Schedule I license.  The individual who is picking up must be part of the research team. No other individuals will be allowed to pick-up and transport the study drug to the study site. A chain of custody form and a medication pick-up log will be completed by both an IDS staff member and the research team member picking up the drug.

## Concomitant Medications and Therapies

### Supportive Care

All supportive measures consistent with optimal subject care may be given throughout the study. Patients will be encouraged to have a supportive care relationship with the social worker on their treatment team throughout the duration of the study and will be encouraged to maintain this therapeutic relationship with their social worker following completion of the study.

### Prohibited Therapy

Medications or vaccinations specifically prohibited in the exclusion criteria are not allowed during the active treatment period. Patients are prohibited from receiving the following therapies during the treatment phase of this trial if randomized to the psilocybin arm:

- Other investigational agents
- Herbal remedies known to potentially interfere with major organ function (e.g., hypericin)
- Monoamine oxidase inhibitors
- Medications known to be uridine diphosphate glucuronosyltransferase enzyme modulators
- Selective serotonin reuptake inhibitor/serotonin-norepinephrine reuptake inhibitors (SSRI/SNRI)
- Serotonin 5HT2A receptor antagonist
- Benzodiazepines
- First and second-generation antipsychotics
- St. John’s Wort

## Duration of Therapy

*MBSR arm*: Total duration of the MBSR curriculum is 8 weeks with a once weekly session (over Zoom or in person) for 2 hours and a full day mindfulness retreat.

*MBSR + PAP arm*: Total duration is the same as above for the MBSR curriculum with a 9 week total duration (given additional integration session following MBSR completion). This arm will include an additional time to incorporate the PAP protocol. This includes three 90min preparatory sessions, one 8 hour psilocybin session (in place of the MBSR retreat), and three 90 minute integration sessions.

### Criteria for discontinuation of study treatment

Patients may withdraw from treatment or the study overall at any time at their own request, or they may be withdrawn at the discretion of the Investigator for safety, behavioral reasons, or the inability of the subject to comply with the protocol-required schedule of study visits or procedures. The following will result in treatment discontinuation:

- Subject requests to discontinue the study treatment and/or study procedures.
- Non-compliance as defined as missing any of the MBSR or PAP sessions.
- Pregnancy
- Significant protocol violation
- The subject refused further treatment
- Study terminated by investigator sponsor
- Lost to follow-up
- Concerns about resultant abuse of study drug or related compounds

### Criteria for discontinuation of study (“off study”)

Subjects will be taken off study for the following:

- The subject completed the study follow-up period
- Screen failure
- Subject is lost to follow-up
- If, in the investigator's opinion, the continuation of the trial would be harmful to the subject's well-being.
- Concerns about resultant abuse of study drug or related compounds
- Development of intercurrent illness or situation which would, in the judgment of the investigator, significantly affect assessments of clinical status and trial endpoints.
- Participant requests to be withdrawn from the study
- Death

# TOXICITIES AND DOSE MODIFICATION

Every effort should be made to administer psilocybin at the planned dose and schedule. In the event of study drug-related adverse event, supportive care and therapy will be administered. Patients are to be instructed to notify investigators at the first occurrence of any adverse symptom. Appropriate follow-up assessments should be done until adequate recovery occurs as assessed by the Investigator.

## Guidelines for the Management of Adverse Events

Since the resurgence of interest in psilocybin as a therapeutic tool in the early 1990s, there have been no reports of any medical or psychiatric serious adverse events. This includes over 2000 doses of psilocybin (in various amounts) that have been safely administered to humans in the United States and Europe, in carefully controlled scientific settings. However, to assure the safety of our participants, protocols will be in place should any adverse events occur.

Emotional difficulties, often termed “difficult experiences” are not necessarily pathological in themselves and can be understood as part of the therapeutic process (e.g., working through cancer-related psychological or existential distress). These displays may include shouting, tearfulness, and other emotive displays that the therapist will attempt to work through with the subject. However, in the case of extreme emotional distress (as defined by the subject) or counter-therapeutic experiences, subject physical and psychological safety will continue to be assured in a systematic manner. Subjects will be offered the following in a stepwise fashion:

1. The group leader will provide additional supportive intervention in cooperation with the subject’s assigned therapist such that the subject’s concerns are addressed to the best of their ability, while maintaining the group setting.
2. If support from the group leader cannot address the individual’s concern after 30 minutes, or if the amount of distress is judged by the group leader to be counter-therapeutic or significant enough to require more individual attention, the subject will then be relocated to an attached private room with their individual therapist where they will be provided with supportive therapy and suggestions for mindfulness strategies. The environment will be crafted as such that the subject is both safe and comfortable.
3. If interventions in the individual room do not alleviate the subject’s concerns after 60 minutes, they will be offered orally administered lorazepam 2mg.
4. At the end of the psilocybin dosing day if there are clinical concerns that a given participant is not safe to leave the site along medical, psychiatric, or cognitive assessments the lead study monitor (one of the PIs) and the Lead Therapist will remain on site for ongoing assessment and support until the participant is deemed safe to depart with a designated ride and support person. If a given participant remains unsafe to leave the site despite ongoing assessment and support they will be escorted to either the HMHI Receiving Center (for psychiatric assessment and care) or the University of Utah Emergency Department depending on either psychiatric or medical clinical presentation. Presenting features that would preclude participants from leaving the study site following the psilocybin dosing day include: acute medical conditions or significant side effects to psilocybin that are not improving and represent clinically concerning risk per MD assessment, acute suicidality or thoughts of self harm or severe emotional distress that is judged to represent clinically significant impairment in functioning or safety concerns based on MD assessment, or cognitive impairments deemed to present clinically significant impairments in functioning based on MD assessment.

Of note, there have been no reports of the necessity of pharmacologic interventions for psychological safety in any well-reported clinical trials with oral psilocybin. However, to assure the safety of the subjects, these materials and protocols will be readily available and in place.

Although are no recorded incidents in controlled studies with similar or higher doses that were deemed serious, protocols will be in place for physical adverse events as well. Most commonly, these include transient mild increases in systolic and/or diastolic blood pressure, mild headache, mild tachycardia, and nausea after psilocybin administration. Hydralazine (25mg), acetaminophen (650mg), propranolol (10mg), and promethazine (25mg) will be available to address these concerns if deemed necessary by a licensed physician in the study after a clinical examination of the study subject. Vital signs will be monitored approximately ten minutes before and 30, 60, 90, 120, 180, 240, 300, and 360 minutes after administration consistent with previous studies. This will include heart rate, respiratory rate, blood pressure, and oxygen saturation. If there is a medical emergency, participants will be escorted by an on-call clinician to the University of Utah Emergency Department by EMS. If there is a psychiatric emergency the participant will escorted by an on-call physician to the HMHI Receiving Center for evaluation.

**Table 1: Adverse Event Management**

| **Adverse Event** | **Subject Management** |
| --- | --- |
| Hypertension | - Elevated systolic blood pressure (>180 mm Hg and/or elevated diastolic blood pressure (>120 mm Hg at one or more time-point) with no other symptoms will be re-checked in 10 minutes. - If the acute elevation of blood pressure continues, hydralazine 25mg will be administered to the subject. - If other physical symptoms are present, blood pressure will be re-checked immediately and the licensed physician will decide the appropriate level of care, whether through medication administration or having the subject escorted to the University of Utah Emergency Department. |
| Headache | - Subjects will be examined by a licensed physician and offered acetaminophen 650mg |
| Tachycardia | - Elevated heart rate (100 – 160 bpm) noted on vital sign monitoring will first be met with therapeutic interactions with the therapist given the likelihood of this being secondary to the subject’s experience. This will continue to be monitored, with a licensed physician examining the subject if the heart rate continues to be elevated. - Elevated heart rate (>160 bpm) will prompt examination by a licensed physician who will examine the subject and triage appropriately whether the subject should receive propranolol 10mg and/or be escorted to the University of Utah Emergency Department. |
| Nausea/Vomiting | - Subjects will be assessed by a licensed physician with an emphasis on the least invasive means, avoiding medication administration if possible. - If nausea/emesis is significant and subject requesting antiemetic, promethazine 25mg will be provided. |
| Other | - A licensed physician will examine the subject and determine the appropriate level of care for the subject |

## Contraception

Non-clinical and clinical data describing the effects of oral psilocybin on lactation, sperm, and teratogenicity are not available. Therefore, women of childbearing potential should have a negative pregnancy test at the time of screening and use a highly effective form of contraception for the duration of study participation and for at least one month after psilocybin administration.

Male subjects should be instructed to use a condom for 24 hours after the psilocybin dose.

Acceptable highly effective contraceptive methods include:

- - Bilateral tubal occlusion
  - Vasectomized partner
  - Intra-uterine device (IUD) or hormone-releasing system (IUS)
  - Any hormonal (estrogen combined with progesterone or progesterone alone) contraception associated with inhibition of ovulation: implanted, oral, intravaginal, transdermal, or injectable.
  - The combination of a barrier method with spermicide (i.e., diaphragm, sponge, or male or female condoms).
  - Abstinence from heterosexual intercourse.

# SCHEDULE OF EVENTS

The Schedule of Events table provides an overview of the protocol visits and procedures. Refer to the Study Assessments and Procedures section of the protocol for detailed information on each assessment required for compliance with the protocol. The investigator may schedule visits (unplanned visits) in addition to those listed in the Schedule of Events table in order to conduct evaluations or assessments required to protect the wellbeing of the subject. This Schedule of Events will be followed for the entire study.

**Table 2: Schedule of Events**

| **Week** | **-5 to -1** | | **1** | **2** | **3** | **4** | **5** | **6** | **7** | **8** | **9** | **11** | **33** |
| --- | --- | --- | --- | --- | --- | --- | --- | --- | --- | --- | --- | --- | --- |
| **Day** | **-35 to -7** | | **0-7** | **8-14** | **15-21** | **Etc.** |  |  |  |  |  |  |  |
| **Study Stage** | **Screen** | | **Active** | | | | | | | | | **Follow-up^[[1]](#endnote-1)^** | |
| Informed consent, registration | x |  |  |  |  |  |  |  |  |  |  |  |  |
| Eligibility criteria | x |  |  |  |  |  |  |  |  |  |  |  |  |
| Demographics | x |  |  |  |  |  |  |  |  |  |  |  |  |
| Medical History | x |  |  |  |  |  |  |  |  |  |  |  |  |
| Vitals and PE^[[2]](#endnote-2)^ | x |  |  |  |  |  |  |  |  |  |  |  |  |
| Randomization/assignment | **x** |  |  |  |  |  |  |  |  |  |  |  |  |
| Expectancy Questionnaires |  | **x** | **x** |  |  |  |  |  |  |  |  |  |  |
| **MBSR ARM** |  |  | **1** | **2** | **3** | **4** | **5** | **6** | **7** | **8** | **9** | **11** | **33** |
| **MBSR course** |  |  | **x** | **x** | **x** | **x** | **x** | **x** | **x** | **x** |  |  |  |
| **Retreat** |  |  |  |  |  |  |  |  | **x** |  |  |  |  |
|  |  |  |  |  |  |  |  |  |  |  |  |  |  |
| **PAP-MBSR** |  |  | **1** | **2** | **3** | **4** | **5** | **6** | **7** | **8** | **9** | **11** | **33** |
| Comprehensive Metabolic Panel ^xi.^ |  | x |  |  |  |  |  |  |  |  |  |  |  |
| Urine drug screen |  | **x** |  |  |  |  |  | x (within 24 hrs) |  |  |  |  |  |
| Pregnancy test ^iii^ |  | x |  |  |  |  |  | x (within 8 days of PAP) |  |  |  |  |  |
| MBSR Course |  |  | **x** | **x** | **x** | **x** | **x** | x | x | **x** |  |  |  |
| Prep Sessions 1-3 |  |  |  |  |  |  | **x** | x |  |  |  |  |  |
| PAP Session |  |  |  |  |  |  |  |  | x |  |  |  |  |
| Vitals monitoring |  |  |  |  |  |  |  |  | **x^[[3]](#endnote-3)^** |  |  |  |  |
| Integration Sessions 1-3 |  |  |  |  |  |  |  |  | **x** | **x** | **x** |  |  |
| **Assessments** |  |  | **1^[[4]](#endnote-4)^** | **2** | **3** | **4** | **5** | **6** | **7** | **8^[[5]](#endnote-5)^** | **9** | **11** | **33** |
| PHQ-9 | x |  |  |  |  |  |  |  |  |  |  |  |  |
| MBI-HSS (MP) | x |  | x |  |  |  |  |  |  |  |  | x | **x** |
| QIDS-SR-16 |  |  | x |  |  |  |  |  |  |  |  | x | **x** |
| Demoralization II scale |  |  | x |  |  |  |  |  |  |  |  | x |  |
| PTSD Checklist 5 |  |  | x |  |  |  |  |  |  |  |  | x | **x** |
| MEQ-30 |  |  |  |  |  |  |  |  | x |  |  |  |  |
| Challenging Experience Questionnaire |  |  |  |  |  |  |  |  | x |  |  |  |  |
| Nondual Awareness Dimensional Assessment (state) |  |  |  |  |  |  |  |  | x | **x** |  |  |  |
| Nondual Awareness Dimensional Assessment (trait) |  |  | **x** |  |  |  |  |  |  |  |  | **x** |  |
| Watts’ Connectedness Scale |  |  | x |  |  |  |  |  |  |  |  | x | **x** |
| Toronto Mindfulness Scale, FFMQ-15 |  |  | x |  |  |  |  |  |  |  |  | x | **x** |
| Brief Savoring Inventory |  |  | x |  |  |  |  |  |  |  |  | x |  |
| **Storyline Health Questionnaires** |  |  |  |  |  |  |  |  |  |  |  |  |  |
| **Storyline Integrative Assessment**: Neurological, Psychiatric, Cognitive, Psychological |  |  | x |  |  |  |  |  |  |  |  | x | **x** |
| Qualitative assessment on workplace functionality ^vii.^ |  |  | x |  |  |  |  |  |  |  |  | x |  |
| Qualitative assessment on relationship between mindfulness training and psilocybin session and group format ^x^ |  |  |  |  |  |  |  | **x** |  |  |  | x |  |
| **Safety** |  |  |  |  |  |  |  |  |  |  |  |  |  |
| CTCAE Adverse Event collection^[[6]](#endnote-6)^ | x |  | x |  | x |  | x | x | x | x | x | x | **x** |
| Assess suicidality: C-SSRS ^x^ | x |  |  |  |  |  |  | x |  | x | x | **x** | **x** |
| Assess concomitant meds | x |  | x |  | x |  | x | x | x | x | x | x | **x** |

^i.^ Follow up measures to be administered 14 days (+/- 3) and 180 days (+/-7) after final MBSR session in week 8.

^ii.^ Vital signs include systolic and diastolic blood pressure, heart rate, respiratory rate, pulse oximetry, weight. Weight captured at screening only.

^iii.^ Pregnancy test (serum or urine) must be obtained at screening and ≤ 8 days prior to psilocybin administration for all women of childbearing potential and as clinically indicated while on trial.

^iv.^ On day of psilocybin administration vital signs will be assessed approx. 10 min before dosing and at 30, 50, 90, 120, 180, 240, 300, and 360 minutes after psilocybin administration. The baseline vitals will be allowed a 60-minute window to collect prior to dosing, while each subsequent vital collection will be allowed a window of +/- 15 minutes.

^v.^ All Questionnaires scheduled for MBSR Week 1 to be administered prior to the first MBSR session. Questionnaires and safety measures assessed similarly across both study arms.

^vi.^ Integration sessions #1 and #2 both included in this week for the PAP + MBSR arm. Adverse event collection, suicidality, concomitant meds will all be assessed x 2 during this week for the PAP+MBSR arm.

^vii.^ This will consist of a 3-minute free-form video recorded response on the Storyline platform by participants regarding perceived functioning in the workplace.

^viii.^ This will consist of a 3-minute free-form video recorded response on the Storyline platform by participants regarding perceived reciprocal effects of mindfulness training and psilocybin session.

^ix.^ Adverse event collection will occur during all 3 preparatory sessions and all 3 integration sessions for the PAP+MBSR arm. Adverse event monitoring can occur via phone contact for intervals prior to psilocybin dosing.

^x.^ If subject is rated as moderate or high risk a full C-SSRS will be completed.

^xi.^ A comprehensive metabolic panel will be done by ARUP laboratories and will be done before the start of MBSR curriculum.

# STUDY PROCEDURES

## Screening

For screening procedures see the Schedule of Events and the Assessments Section. Screening activities may only begin after a subject has signed consent. Consent signing can occur electronically via REDCap. All screening activities must take place within ≤ 35 days prior to the first MBSR unless otherwise noted.

During screening, subjects will also be provided with a list of resources including reading materials and on-line resources if they would like to further investigate the topic of psilocybin-assisted psychotherapy. Consenting can be completed via paper or electronic forms. Participants will be administered the MBI-HSS(MP) and the PHQ-9 to evaluate eligibility for participation prior to consent. A waiver of consent will need to be obtained by the IRB to administer questionnaires prior to consenting.

## Randomization and study arm assignment

Participants who meet eligibility criteria and have consented and enrolled in the study will be randomized to either the MBSR Arm or the MBSR + PAP arm using a randomization plan generated by the SAS code, PROC plan. The study will be open label and participants will be informed as to study arm assignment prior to the first MBSR session. There will be rolling enrollment in trial participation up until the cut-off registration date prior to each MBSR course starting date. Each arm will be run independently given likely variation on numbers of participants willing to complete each study arm however efforts will be made to run both arms concurrently depending on recruitment. While the MBSR only arm can be completed on an individual basis for each participant the MBSR + PAP arm includes group-based psilocybin-assisted therapy interventions which requires 2-5 participants per MBSR cohort. We will leave open the option of running group PAP sessions for a cohort of only 2 participants however will have a goal participant number per cohort of 4 with the possibility of running up to 5 participants per cohort through this arm. To assess expectancy effects on treatment response participants will be administered expectancy scales prior to randomization to assess preference as well as post randomization to assess credibility.

## MBSR Arm

Participants randomized to the MBSR only arm will be enrolled in the MBSR curriculum led by Trinh Mai LCSW through the University of Utah Resiliency Center. If group size issues arise to complicate feasibility we will incorporate MBSR groups from outside institutions, also paid for by the study. This course is based on the evidence-based model developed by Jon Kabat-Zin at the University of Massachusetts Medical Center. Cost for University of Utah employees ($99) will be covered through the study. Groups meet for an orientation, 9 weekly classes via Zoom of in person (2-2.5 hours each), and an all-day retreat (in person or via Zoom depending on Covid restrictions at the time) held in the final two weeks of the curriculum Guided instruction in various practices is provided, including sitting and walking meditation, body scan, gentle yoga, and mindful communication. These practices are enhanced through inquiry exercises, group dialogue, and daily home assignments. If capacity of internal MBSR courses limit feasibility of the study we will utilize MBSR groups run through Intermountain or Davis Behavioral Health.

<https://app.healthcare.utah.edu/peakCourseRegistration/byCourse;jsessionid=D5D05B9057AA596202DA3A1DD3A81BDE?primaryId=609>

Instruction focuses on three formal techniques: mindfulness meditation, body scanning, and simple yoga postures. Group discussion is a central part of this program, along with regular mindfulness meditation homework assignments which will ideally be practiced daily by participants. Body scanning is the first prolonged formal mindfulness technique taught over the first four weeks of the curriculum. Body Scanning, also known as progressive relaxation, is a type of meditation that utilizes paying close attention to each body part as a way of focusing the mind and becoming more aware of the present moment. Mindfulness meditation is a technique which will build upon this foundation. A technique which has been practiced for thousands of years, participants will focus on different sensations, such as breathing, with a goal to quiet the mind and increase awareness. Basic yoga postures will be used as a stretching practice and a form of moving meditation. MBSR has been validated to help with anxiety and depression (Khoury 2013) and there is reason to suspect it will be helpful with burnout as well.

## MBSR + PAP arm

### MBSR + PAP Preparatory Sessions

Once a subject has completed screening, has been found to be eligible, and has been registered and assigned to the MBSR +PAP arm, Preparatory Sessions may begin with flexible scheduling as indicated in timeline above given scheduling challenges with healthcare providers. See the Schedule of Events and the Assessments Section for treatment period procedures.

The same general themes will be covered during the group Preparatory Sessions.

#### Pre-Psilocybin Session #1:

The group leader will introduce the following topics: establishing safety and boundaries, an introduction of set/setting, an introduction to psilocybin effects, and a discussion of possible difficult experiences including death/transcendence of ego. Participants will be encouraged to remember the following phrases: “Trust/Let Go/Be Open”, “Accept/Be”, and “All is Welcome”

#### Pre-Psilocybin Session #2:

Topics will include a review of psilocybin treatment setting, rules of conduct during psilocybin session including how to handle occasional bathroom/stretching breaks, review of safety guidelines including the rule about no leaving the treatment area, how to handle fear and other challenging emotions, review of rules around physical touch between therapist and participant, review of the use of eyeshades and headphones, and instructions about music.

#### Pre-Psilocybin Session #3.

Topics for Preparatory Session 3 will include a review of the treatment day, the importance of eating a light breakfast, having limited caffeine intake on treatment day, availability of fruit/cheese/crackers later in the treatment day and possibly a light sandwich, the process of monitoring vital signs, the availability of bins for nausea, other unlikely scenarios that might occur including loss of bladder control, fire alarm procedures, and a private room for anyone experiencing emotional distress. A walkthrough of the treatment space will be incorporated.

### MBSR + PAP Psilocybin Session

Each participant will have an individual station in the large group room. They will be provided with eyeshades, blanket, and pillow. They may bring their own token objects of personal significance or meaning that are related to setting an intention for the session.

A brief group meeting will occur starting at approximately 8:00 a.m. The group leader will review the day’s agenda and set the intention for the group. General safety rules and guidelines will be reviewed, as well as procedures for using the restroom.

Participants will then break out with their assigned therapist to set up their station and get ready for medication administration.

Psilocybin will be administered by the group leader at approximately 9:00 a.m. following obtaining a set of baseline vital signs.

A communal music track will be played.

There will be an adjacent individual “break-out” room that a subject plus therapist can move to if there is a perceived need for a period of privacy or a particularly emotionally challenging session that might risk disrupting the group atmosphere for the other participants.

Light snacks and beverages will be available after approximately 1:00 pm.

If a therapist needs a break, the lead therapist will step over to sit with the subject.

At approximately 4:00 pm, the study team will do a brief assessment of each participant to assess whether the medication effect is tapering downward as expected.

At approximately 4:30, participants will complete a short battery of assessments and questionnaires- see Schedule of Events. All participants will have a brief exit assessment by the lead therapist and study team to ensure the patients are safe to go home. This will include evaluation of physical symptoms and functioning, a psychiatric evaluation as to acute suicidality and significant emotional distress, and a cognitive evaluation as to orientation.

Between 5:00-5:30 pm participants, will be allowed to leave with their escorts and return home. They will be instructed to have a quiet evening and a light meal.

An individual therapist as well as the study team will be available by phone for the entire time period between the completion of the psilocybin session and the first integration session.

### MBSR + PAP Integration Sessions

The first Integration Sessions will occur within ≤ 48 hours of completion of the Psilocybin Session. This will be followed by two additional integration sessions. Integration sessions will consist of large group discussion and sharing of what occurred during the psilocybin session. General themes will be explored including how to integrate the psilocybin experience with daily life. Guidance will be offered regarding how subjects can continue to process their psilocybin experience.

### MBSR + PAP Follow-Up

Subjects will have the phone number of their individual therapist throughout the duration of the follow-up period, which will extend until the second set of questionnaires are administered 2 weeks following the completion of group intervention sessions. Subjects will be followed for acute suicide ideation for 28 days post-psilocybin administration and also assessed for sustained response 180 days (+/-7) post-psilocybin administration as indicated on the Schedule of Events.

# STUDY ASSESSMENTS

Every effort should be made to ensure that the protocol-required tests and procedures are completed as described. However, it is anticipated that from time to time there may be circumstances, outside of the control of the Investigator that may make it unfeasible to perform the test. In these cases, the Investigator will take all steps necessary to ensure the safety and well-being of the subject. When a protocol-required test cannot be performed, the Investigator will document the reason for this and any corrective and preventive actions that he or she has taken to ensure that normal processes are adhered to as soon as possible.

## Physical Examinations and Vital Signs

Participants will have physical examinations to include major body systems, vital signs, assessment of medical history, and weight (weight will be measured at screening only) at the time points described in the Schedule of Events. Vital signs will be monitored within 60 minutes before and 30, 60, 90, 120, 180, 240, 300, and 360 minutes after administration, which is consistent with previous studies.

Vital signs, including blood pressure, pulse rate, respiratory rate, and oxygen saturation will be also recorded at the time points described in the Schedule of Events. Vital signs will be taken prior to the administration of any investigational products at the visit.

## Adverse Events

Adverse events experienced during trial participation will be collected per the Schedule of Events and Adverse Events Section. Each study participant will be questioned about the occurrence of adverse events in a non-leading manner. Should the treating investigator feel that the adverse event is attributed to study therapy, then guidelines for subject management in Section 7.1 will be followed.

An Adverse Event (AE) is defined as any medical occurrence in a participant, including any abnormal sign (e.g., abnormal physical exam or laboratory finding), symptom, or disease, temporally associated with the participant’s involvement in the research, regardless of whether it is considered related to participation in the research. This definition includes concurrent illnesses or injuries and exacerbation of pre-existing conditions.

Events related to planned treatments or physician visits for baseline conditions collected in the medical history will not be collected, unless there is an exacerbation of the condition, in which case they will be actively followed until resolution.

The site physician will be responsible for reviewing and confirming all AEs and SAEs collected during the study. The therapy teams will collect Aes during study visits from Enrollment through Study Termination. Participants will be asked directly how they are feeling during each contact, and Aes may be captured spontaneously during psychotherapy sessions, telephone calls, or other correspondence. Completed measures may create suspicion that an AE occurred; in this case, the site staff should follow-up with the participant.

Study personnel will also assess AEs related to possible abuse of study drug or related compounds and will be trained in the identification of concerning signs of abuse.

## COVID-19 Precautionary Measures

Due to the COVID-19 pandemic, precautionary measures will be taken to ensure subject safety and trial integrity as clinically indicated and based on University of Utah Hospital and Clinics current standards.

1. As indicated per standard clinical proceedings, possible testing for all study participants and therapists within ≤ 48 hrs. of the first group session along with symptom checklist. A positive test is an exclusion.
2. Subjects and therapists will be screened for COVID-19 symptoms and possible sick contacts prior to each group/psilocybin session. The following will exclude them from participation:
   - Current symptoms of COVID-19
   - Recent positive test for COVID-19

Back up therapists will be available if a therapist is unable to participate in a session. These therapists will have completed the same training requirements.

1. As clinically indicated, group sessions will be done with social distancing (6-foot margins). During psilocybin session, subject/therapist pairs will be allowed physical contact, but not between other dyads, and only if #1 and #2 above are verified.
2. Investigators will provide pre-study education regarding the infection precautions and rationale, revisiting these throughout the group work in anticipation of the psilocybin sessions.
3. Therapists and study team will follow COVID-19 protocols as clinically indicated based on institutional policy at the time as it relates to masking.

## Monitoring for Suicide Ideation

Subjects will be monitored for acute suicide ideation after psilocybin administration. This monitoring may occur in person, by telemedicine, or over the phone. An appropriately trained and delegated Investigator will assess the subject for the occurrence of new or increased intensity of suicide ideation or suicide attempts at the time points indicated in the Schedule of Events. A clinical assessment for acute suicidal ideation will be performed following the psilocybin dosing session prior to participants leaving the site.

## Laboratory Assessments

Samples for all laboratory assessments will be drawn at the time points indicated in the Schedule of Events and when clinically indicated. All safety laboratory analyses will be performed by the local laboratory. All safety laboratory assessments must be reviewed by the treating investigator prior to study drug administration. When applicable, results from the pregnancy test must also be available for review prior to dosing. Laboratory screening will only be required for participants randomized to the MBSR + PAP arm to ensure safe and effective administration of study drug.

**Table 3: Laboratory Assessments**

| **Laboratory Assessments** | |
| --- | --- |
| **Complete Metabolic Panel** | - Sodium - Potassium - Chloride - Carbon Dioxide - Alkaline Phosphatase - Aspartate Aminotransferase - Alanine Aminotransferase - Urea Nitrogen - Glucose - Creatinine - Calcium - Protein - Albumin - Bilirubin - Anion Gap |
| **SARS-CoV-2** | - COVID-19 nasopharyngeal swab |
| **Pregnancy** | Beta-hCG Qualitative Serum at screening. Beta-hCG Qualitative Urine within 24 hours of psilocybin administration. |
| **Urine Drug Screen** | - Benzodiazepines - Barbiturates - Stimulant amines - Cocaine - THC - Opioids |

## Response Assessment

The response to study therapy will be assessed utilizing the QIDS-SR-16 and MBI-HSS(MP) at the time points indicated in the Schedule of Events. Subjects will be contacted directly by study personnel and arrangements made for the administration of the questionnaires in a consult room at the Huntsman Mental Health Institute. If necessary, telemedicine or the Storyline Health platform may be used to administer these questionnaires. In either case, the questionnaires will be filled out by an appropriately trained and delegated Investigator upon subject assessment.

# CRITERIA FOR EVALUATION AND ENDPOINT

## Safety

Routine safety and tolerability will be evaluated from the results of reported signs and symptoms, scheduled physical examinations, vital sign measurements, and clinical laboratory test results. More frequent safety evaluations may be performed if clinically indicated or at the discretion of the investigator.

## Efficacy

The efficacy of the study therapy will be determined by the change in depression scores as measured by the QIDS-SR (primary) and burnout scores as measured by the MBI-HSS(MP) (secondary).

## Stopping Rules

For 14 days following psilocybin administration, subjects will be monitored for adverse events. Study will be placed on hold and the study will be reevaluated if there is an occurrence of any grade 3 (or above) adverse event attributed to psilocybin in the first cohort of participants. These include hypertensive emergency or other serious cardiac events and other need for hospitalization attributed to psilocybin administration. For 28 days following psilocybin administration, subjects will be monitored for acute suicide ideation and suicide attempt. Any suicide attempt throughout the trial will also preclude further enrollment. Any attempt whether completed or not will be recognized as a serious adverse event and warrant study closure. Subjects’ physical and psychiatric states will be closely followed throughout the trial and for 28 days after psilocybin administration. If neither stopping rule is met, then the study will open for continuation. There will be no further administrations of study drug within this 28 day window. If the stopping rule is reached prior to the end of enrollment, the analyses will still be performed for those patients who have completed treatment prior to the time the stopping criterion is reached. In this case, the statistical power for the analyses may be reduced.

# STATISTICAL CONSIDERATIONS

## Population for analyses

12.1.1 Safety Data Set. All participants enrolled in the trial will be eligible for the safety data set. AEs and suicidality per C-SSRS will be assessed across both study arms.

12.1.2. Evaluable for intent-to-treat (ITT) analysis. All participants enrolled in the trial and randomized to either MBSR + PAP or MBSR only will be eligible for the (ITT) analysis. The primary analysis will involve intention to treat analyses on all study outcomes.

12.1.2 Evaluable for per-protocol analysis. Participants in the MBSR arm who have completed two-thirds of the MBSR sessions will be considered evaluable for the per-protocol efficacy data set. Participants in the MBSR + PAP arm who have completed two- thirds of the MBSR sessions (excepting the MBSR retreat) *as well as* two out of three of the preparatory sessions, the psilocybin therapy session, two out of the three integration sessions, and a baseline and post-psilocybin QIDS-SR and MBI will be considered evaluable for the per-protocol efficacy data set.

## Statistical Analyses

Aim 1 is to assess the safety and feasibility of MSBR and PAP for burnout. We will track frequency and percentage of adverse events and rates of serious adverse events utilizing the Common Terminology Criteria for Adverse Events (CTCAE) v.5 from study initiation until termination, as well as the Columbia Suicide Severity Rating Scale (C-SSRS). We will also track qualitative data from participant and clinician direct reporting as well as outcomes with the Challenging Experience Questionnaire (ChEQ).

Aim 2 is to assess the efficacy of MBSR+PAP vs. MBSR alone in reducing symptoms of depression, burnout, demoralization, and other clinical symptoms. For continuous outcomes, the analysis will be performed under a generalized linear mixed model (GLMM). The model will include follow-up time as a categorical predictor variable (baseline, 2 weeks, 6 months) as well as the interaction of this categorical factor with the randomized treatment group. Our primary interest lies in the Treatment X Time interaction, expressible as a linear contrast on the β parameters. Repeated effects will incorporate autoregressive dimensioning of the linear predictor, if needed and supported by the Bayesian Information Criterion. In the case that pre-randomization between-groups differences are present in the baseline values of outcome variables, effects will be controlled for chance baseline imbalance using an analysis of covariance strategy that conditions on baseline values. We will use maximum likelihood estimation of missing data for intent-to-treat analyses of all study outcomes.

The statistical model is expressed mathematically as below:

$$Y_{it}=\alpha_{00}+{\alpha_{10}Trt}_{i}+{\alpha_{20}Time1}_{it}+{\alpha_{21}Time2}_{it}+$$

$${\alpha_{30}Trt}_{i}{Time1}_{it}+{\alpha_{31}Trt}_{i}{Time2}_{it}+Z_{i}\beta_{i}+\epsilon_{ijt},$$

$$\beta_{i}\sim N\left( 0, \psi^{2} \right) \left( EQ1 \right)$$

$\epsilon_{it}\sim N\left( 0, \phi^{2}\lambda_{itt} \right), Cov\left( \epsilon_{it}, \epsilon_{it^{'}} \right)=\phi^{2}\lambda_{itt'}$

In EQ1, $Y_{it}$ denotes the continuous outcome measure for depression or burnout score measured at the $t^{th}$time (t=0, 1, 2 for baseline, 2^nd^ week and 6^th^ month) for the $i^{th}$ participant, $\alpha_{00}$ is the intercept, which is the effect for the MBSR alone group at baseline, $\alpha_{10}$ is the treatment effect difference between MBSR+PAP vs. MBSR alone group at baseline, ${Trt}_{i}$ is a 0-1 indicator variable which takes the value 1 when the $i^{th}$ participant is assigned to the MBSR+PAP group, otherwise 0, $\alpha_{20}$ is the difference between the 2^nd^ week and baseline for MBSR alone group, $\alpha_{21}$ is the difference between the 6^th^ month and baseline for MBSR alone group, ${Time1}_{it}$ and ${Time2}_{it}$ are 0-1 indicator variables which take the value 1 when t=1 (2^nd^ week), t=2 (6^th^ month, $\alpha_{30}$ is the extra treatment effect difference between MBSR+PAP and MBSR alone group when compare the 2^nd^ week to baseline, $\alpha_{31}$ is the extra treatment effect difference between MBSR+PAP and MBSR alone group when compare the 6^th^ month to baseline, $Z_{i}$is a 0-1 indicator variable which take the value 1 for the $i^{th}$ participant, otherwise 0, $\beta_{i}$ is the participant effect for the $i^{th}$ participant, $\psi^{2}$is the variance of the random participant effect ($\beta_{i}$), $\epsilon_{it}$ is the residual at the $t^{th}$time for the $i^{th}$ participant, $\phi^{2}\lambda_{itt'}$ is the covariance between the $t^{th}$ and the ${t'}^{th}$time.

The coefficient of $\alpha_{30}$ is the difference of reduction in depression/burnout from baseline to the 2^nd^ week comparing the two groups, which constitutes the efficacy of the MBSR+PAP. The coefficient of $\alpha_{31}$ are the difference from baseline to the 6^th^ month, which constitutes the long-term effect of the outcome. For outcomes with only two timepoints, the coefficient of $\alpha_{31}$ will be dropped.

In an exploratory analysis, burnout measured as a binary variable (e.g., burnout vs. not-burnout) will be evaluated using generalized linear mixed effects models assuming a binomial distribution with probit (cumulative logistic) link. The probit link gives results very similar to the logit link, but yields a more natural interpretation in terms of an underlying propensity.

We will also assess the correlation between magnitude of change in burnout scores on the MBI using Pearson correlation. Path analysis will be used to assess changes in the Mystical Experience Questionnaire (MEQ-30), Challenging Experience Questionnaire, and Nondual Awareness Dimensional Assessment (NADA) as mediators of the effect of treatment (MBSR+PAP vs MBSR alone) on burnout, using bootstrapping to assess the significance of the indirect effect (Preacher & Hayes, 2008).

### Statistical Power

### Power is based on the efficacy of MBSR+PAP vs. MBSR alone in reducing symptoms of depression. A meta-analysis examining the effects of psilocybin on depressive symptoms reported an effect size of Cohen’s d=1.29. Previous meta-analyses of MBSR indicate modest to moderate effect sizes for depressive symptom reduction (Cohen’s d= 0.3 to 0.4). We conducted simulation-based power calculations for the linear mixed-effects model. Assuming a **large between group effect size (Cohen’s d = 1.0)**, alpha=0.05, and a **moderate-to-high intra-class correlation (ICC = 0.5)**reflecting the repeated measures design, the estimated power to detect a **treatment group × time interaction** in the LMM was approximately **81%**. Given the pilot nature of this study, the primary goal was to estimate effect sizes and assess feasibility to inform future larger-scale trials.

### Sample size determination:

QIDS-SR-16: Prior studies that have utilized depression rating scales (HAM-D) (Griffiths et al. 2016) have reported within-group effect sizes of 1.30 and 1.23, respectively (with cancer patients with depression and anxiety symptoms). This was with an 82% completion rate of randomized subjects. Additionally, 4 small studies looking at pre-post measures of depression with a psilocybin-assisted therapy intervention (N=69) have demonstrated a pooled standardized effect size of Hedges g 1.38 (Goldberg 2020).

Determining sample size in relation to the MBI and symptoms of burnout is more difficult given less precedent in the literature. Demoralization is a closely related and overlapping concept and Anderson et al. (2020) investigated response to demoralization (Demoralization II Scale) in a psilocybin-assisted group intervention with patients with AIDS. This study demonstrated an effect size (Cohen’s d) of 0.97. Using this effect size and an alpha of 0.05, a sample size of 18 patients would provide 80% power to detect this difference between groups.

We conducted simulation-based power calculations for the linear mixed-effects model. Assuming a **large between group effect size (Cohen’s d = 1.0)**, alpha=0.05, and a **moderate-to-high intra-class correlation (ICC = 0.5)**reflecting the repeated measures design, the estimated power to detect a **treatment group × time interaction** in the LMM was approximately **81%**. Given the pilot nature of this study, the primary goal was to estimate effect sizes and assess feasibility to inform future larger-scale trials.

### Primary Analysis:

- 1. We will track rates of adverse events and rates of serious adverse events utilizing the CTCAE (NCI Common Terminology Criteria for Adverse Events Version 5.0) for adverse events (AE) and serious adverse events (SAE) reporting, as well as the Columbia Suicide Severity Rating Scale (C-SSRS). We will also track qualitative data from participant and clinician direct reporting as well as outcomes with the Challenging Experience Questionnaire (ChEQ).
  2. *QIDS-SR-16:* The analysis of QIDS-SR-16 will use mixed-effects models with fixed effects for time (enrollment, 2 weeks intervention after the last session in each intervention arm), and a random intercept term. The outcome variables are the estimated change from baseline to 2 weeks post completed intervention (full protocol) for both arms. Change from baseline in total score will be analyzed using a generalized linear mixed model (GLMM). A secondary analysis of these scales to determine long term effects of the intervention will include fixed effects at three time points: enrollment, 2 weeks post intervention, and 6 months post psilocybin session.

### Secondary Analysis:

- 1. *MBI:* Our operationalized definition of burnout is consistent with existing literature and will allow us to track rate of remission in participants. The outcome variable for the MBI is the mean change from baseline to 2 weeks post full intervention and 6 months post intervention. Change from baseline in total score will be analyzed using a generalized linear mixed model (GLMM). In addition, we will examine effects of treatment condition on burnout analyzed as a binary outcome (with participants either surpassing or not surpassing the MBI cut point for burnout: emotional exhaustion subscale ≥ 27; depersonalization subscale ≥ 13; or personal accomplishment subscale ≤ 21).
  2. A secondary analysis will include fixed effects at enrollment, 2 weeks post intervention, and 6 months post psilocybin session
  3. *Demoralization II Scale:* score 0-4 low, 5-16 moderate, 17 and above high. Improvement in 2 points on the 16-item scale is clinically significant. The outcome variable is the mean change from baseline to 2 weeks post full intervention which will be analyzed with a GLMM as described above. Prior studies measuring change on this scale with a PAT intervention suggest an effect size of 0.97 (Anderson 2020).
  4. *PTSD Checklist 5 (PCL-5):* 20-item self-report measure. Will be scored according to total symptom severity score (range 0-80) where a 5-10 point change is seen to represent reliable change and a 10-20 point change is seen to represent clinically significant change. The outcome variable is the mean change from baseline to 2 weeks post full intervention which will be analyzed with the use of a GLMM as described above.
  5. Additional secondary questionnaire efficacy outcome variables will be analyzed using a mixed-effects for time (enrollment and 2 weeks post-completion of group intervention) and a random intercept term. The scales will include the McGill Quality of Life Questionnaire (MQOL) and the Watts’ Connectedness Scale (WCS).

### Alpha determination:

Miller et al. (2019) discuss a method for estimating the optimal alpha that accounts for base rate (<https://www.ncbi.nlm.nih.gov/pmc/articles/PMC6314595/>). Base rate of depression symptom improvement with mindfulness alone was estimated at 66-76% (<https://www.ncbi.nlm.nih.gov/pmc/articles/PMC2791668/#B5>), based on effect sizes from Khoury et al. (2015). Using this base rate and an anticipated sample size <50, alpha was set at a conventional 0.05. Due to the early phase nature of this trial, we will not correct the alpha level of secondary clinical outcomes for multiple comparisons.

### Exploratory Analysis

### Develop an algorithm with Storyline Health technology to rapidly discover the most predictive and information rich components in the assessment of burnout and its treatment response, utilizing the Storyline Integrative Assessment

1. The objective is to create an effective and fast algorithm for feature selection and question selection to build assessments, diagnostic biobehavioral markers and predictive models from Storyline captured speech, vocal and facial data. The proposed algorithm has 3 components, including (1) rapid feature and question filtering based on variance, (2) feature selection using a method that integrates mutual information and random forest importance scores and (3) question selection using a deep learning method that ranks the predictive value of each question using the optimized features. The steps are as follows:

*Rapid Question Filtering* – The first step in the Short Story algorithm will be to filter questions, assessments and A.I. features in the storyARC and storyTIME files that have little information content. To do this, we will compute the variance for each measured feature for each question in the data for all people in a customer’s pilot study. The variance is scaled to account for differences in units. We then sum the variance for each feature for each question, apply a min-max absolute scale for interpretation that scores questions from 0 (low information content) to 100 (high information content). The results will enable users to rapidly screen, rank and prune questions with little to no information content by pruning the bottom 25% of tested candidate questions.

*Feature Selection:* Storyline AI measures over 20,000 speech, vocal and facial features from the responses for each question and many are correlated or not useful for detecting some phenotypes of interest. Therefore, in this second step, Short Story technology performs a feature selection analysis that uncovers the most useful features for each tested question in an assessment for predicting a particular phenotype or outcome. The major objective is to uncover the best questions and features when the available data has limited sample size and classes are imbalanced. Our approach will involve implementing the qualitative mutual information (QMI) feature selection algorithm previously detailed by others and proven for high dimensional data, including microarray data for tens of thousands of genes (Nagpal and Singh, 2019). The algorithm will provide a score that combines random forest (RF) feature importance scores with mutual information to obtain a QMI value for each feature. QMI is more powerful than random forest (RF) importance or mutual information alone for feature selection, as previously described (Nagpal and Singh, 2018). In brief, RF will be applied to the speech, vocal and facial feature sets separately for each question and the importance scores of each feature will be calculated, as well as mutual information. RF importance scores will be converted to Preference scores and multiplied by the mutual information to define QMI values for each feature. The features will then be ranked by QMI values and features with a QMI > 0 will be retained. The results will return the most useful speech, facial and vocal features for each question. The usefulness of the features is determined from their ability to differentiate the two classes in a study, such as control versus sick patients.

This approach has many advantages, including broad utility with few parameters to tune, applications to diverse data types with few samples and acceptable computational speed. RF importance scores are powerful for ranking correlated features. Mutual information, which determines the amount of information one random variable can provide about another variable, is then incorporated to further help remove irrelevant and redundant features. When accounting for accuracy and computational efficiency, this combined approach outperforms most methods, including filter, wrapper, embedded and hybrid feature selection strategies (Nagpal and Singh, 2019).

*Question Selection:* The final step of the Short Story algorithm will provide a score that determines the individual questions that should be retained in a new assessment and those that can be eliminated. In this approach, the input is all of the candidate questions and their optimized speech, vocal and facial features revealed from Short Story Score #2. The individual questions and question combinations that show the most value for predicting the classes of interest will then be determined. We will create a simple two-layer neural network that is architected to take Storyline speech, vocal and facial features as inputs to predict the phenotypic classes of interest. The algorithm will be designed such that questions are tested in all possible pairwise combinations with 5-fold cross validation. The mean accuracy for predicting the target phenotype is determined for each question combination. Each pairwise question combination is then assigned an index. To compute the Short Story score for each question, we multiply the index and the mean classification accuracy for all question pairings in which that question was included in the predictive model. This vector of index*accuracy values is summed to yield a score and then the scores for all of the candidate questions are min-max scaled to create the final Short Story question ranking score from 0-to-100, where a high score indicates important questions, and a low score indicates questions that can be pruned. The final results will return the ranking for each question and the mean classification accuracy values for each question.

**Storyline Health Assessments:**

- Patients will be notified through the Storyline app on their smartphone or smart device to complete the various study assessments. Storyline manages reminders and makes the process simple.

## Storyline Integrative Assessment - Neurological, Psychiatric, Cognitive and Psychological

© Copyright Storyline Health 2022

**Summary:**

This 25-minute assessment enables deep, objective profiling of neurological, psychiatric, cognitive and psychological phenotypes and symptoms using artificial intelligence. Patients perform the interactive video interviews and questionnaires on their smartphone and their responses are video recorded and stored in the cloud in the HIPAA, GDPR and HiTech compliant Storyline Vault, which provides state-of-the-art military grade security and is specialized for health data privacy. The video and audio data is analyzed by Storyline AI algorithms that measure >20,000 facial, vocal and speech micro-expressions to monitor symptoms and discover biomarkers and predictive models. Applications include drug treatment studies, basic research of brain functions and health, monitoring wellbeing, biomarker discovery, predictive model discovery, assessment discovery, disease modeling and precision medicine applications. See Appendix 1.

- The Data Monitoring section outlines how participant data is collected, stored and managed through the Storyline Health platform. Participants are owners of their data and can access it at any time, and even delete it. However, the study will not be able to provide direct meaningful interpretations for participants. Note, that participants may be able to choose some non-research interactions within the Storyline app, such as what celebrities they look like. This is not considered a benefit nor will be included in the research data, it’s for entertainment purposes for the participants. Please see section 15.4 on **Storyline Health Data Confidentiality and Security.**
  1. Toronto Mindfulness Scale (TMS), Five-Facet Mindfulness Questionnaire (FFMQ), CERQ Positive Reappraisal Questionnaire.

**Trait Mindfulness** will be measured the Five Facet Mindfulness Questionnaire (FFMQ), with higher scores indicating greater trait mindfulness. **State Mindfulness** will be measured the Toronto Mindfulness Scale (TMS), with higher scores indicating greater state mindfulness. **Reappraisal** will be measured via the positive reappraisal subscale of the Cognitive Emotion Regulation Questionnaire (CERQ), with higher scores indicating more frequent use of reappraisal. These measures will be used to ascertain whether PAP augments therapeutic mechanisms known to be targeted by MBSR.

Effects of MBSR+PAP vs MBSR on these outcomes will be evaluated using GLMMs similar to those used to examine depression and burnout. Fixed effects consist of a time factor and between-subjects treatment factors. Our primary interest lies in the Treatment X Time interaction, expressible as a linear contrast on the β parameters. Repeated effects will incorporate additional dependence, if needed and supported by the Bayesian Information Criterion (BIC), in the form of autoregressive of the linear predictor.

- 1. Path analysis will assess changes in the Mystical Experience Questionnaire (MEQ-30), Challenging Experience Questionnaire, Nondual Awareness Dimensional Assessment (NADA)- both state and trait versions, and Brief Savoring Inventory (BSI) as mediators of the effect of MBSR+PAP on burnout, using bootstrapping to assess the significance of the indirect effect (Preacher & Hayes, 2008). These will be summarized with descriptive statistics, including mean, median, range, standard deviation, and 95% confidence intervals.

# REGISTRATION GUIDELINES

Study-related screening procedures can only begin once the subject has signed a consent form. Patients must meet all of the eligibility requirements listed in Section 5 prior to registration. Patients must be registered before receiving any study treatment and must begin treatment as soon a logistically possible after registration.

# DATA SUBMISSION SCHEDULE

The Case Report Forms (CRFs) are a set of (electronic or paper) forms for each subject that provides a record of the data generated according to the protocol. CRFs should be created prior to the study being initiated and updated (if applicable) when amendments to the protocol are IRB approved. These forms will be completed on an on-going basis during the study. The medical records will be a source of verification of the data. During the study, the CRFs will be monitored for completeness, accuracy, legibility, and attention to detail by a member of the Research Compliance Office. The CRFs will be completed by the Investigator or a member of the study team as listed on the Delegation of Duties Log. The data will be reviewed no less than annually by the Data and Safety Monitoring Committee. The Investigator will allow the Data and Safety Monitoring Committee or Research Compliance Office personnel access to the subject source documents, clinical supplies dispensing and storage area, and study documentation for the above-mentioned purpose. The Investigator further agrees to assist the site visitors in their activities.

**Data capture should be restricted to endpoints and relevant subject information required for planned manuscripts.**

# ETHICAL AND REGULATORY CONSIDERATIONS

## Human Subject Protections

The study will be conducted in accordance with the appropriate FDA, IRB, ICH GCP, and other federal and local regulatory requirements, as applicable. Informed consent will be obtained from all research participants prior to performing any study procedures using the most recent IRB-approved version. All patients must be at least 18 years of age to participate.

## Institutional Review

Before study initiation, the investigator must have written and dated approval/favorable opinion from the IRB/IEC for the protocol, consent form, subject recruitment materials (e.g., advertisements), and any other applicable subject-facing documents. The investigator should also provide the IRB/IEC with a copy of the Investigator Brochure or product labeling information.

The investigator or designee should provide the IRB/IEC with reports, updates and other information (e.g., expedited safety reports, amendments, and administrative letters) according to regulatory requirements or institution procedures.

## Data and Safety Monitoring Plan (see DSMB protocol)

A Data and Safety Monitoring Board (DSMB) will be established by our team consisting of multiple stakeholders to ensure the well-being of patients enrolled on Investigator Initiated Trials that do not have an outside monitoring review. We intend to follow the roles and responsibilities of the DSMC are set forth in the NCI-approved Data and Safety Monitoring (DSM) plan. The activities of the committee include reviewing adverse events (including SAEs), deviations, important medical events, significant revisions or amendments to the protocol, reviewing CRFs, and approving cohort/dose escalations. If the DSMB and/or the PI have concerns about unexpected safety issues, the study will be stopped and will not be resumed until the issues are resolved.

This study is classified as high risk per the NCI-approved DSM plan.  The IRB and DSMB will be notified of all serious adverse events (SAEs).  All serious events will be reviewed by the full DSMB monthly.

**PI and Co-PI**

**John Hendrick, MD** and **Benjamin Lewis, MD**, both Co-PI's on the trial, will serve as primary safety monitors during this study. Dr. Hendrick is an Assistant Professor of Emergency Medicine and Palliative Care at the University of Utah. He is a board-certified Emergency Physician with more than 7 years of experience in critical care. In addition, Dr. Hendrick is board certified in Hospice and Palliative Medicine. Dr. Lewis is Assistant Professor of Psychiatry at the University of Utah. He is a board-certified clinical psychiatrist He has more than 10 years of experience with clinical care and recently completed the CIIS certification in Psychedelic Science. Both Dr. Hendrick and Dr. Lewis have been involved with the planning and operation of the HOPE trial at Huntsman Cancer Hospital.

**DSMB** **members**

**(chair) Brian James Mickey, MD PhD** (Associate Professor of Psychiatry, University of Utah) is a board-certified Psychiatrist and researcher.

**Brent Kious, MD PhD (Assistant Professor of Psychiatry, University of Utah) is a board certified psychiatrist, researcher, and bioethicist.**

**Timothy Fuller, MD** (Assistant Professor of Emergency Medicine and Palliative Medicine, University of Utah) is board certified in Emergency Medicine and Hospice and Palliative Medicine.

**Eric Dooley-Feldman** (Counseling Psychology Doctoral Candidate, University of Utah) is a PhD candidate in Counseling Psychology.

**Tomas Melicher** (Assistant Professor of Psychiatry, University of Utah) is a board-certified psychiatrist.

Serious adverse events include life-threatening cardiac arrhythmias, respiratory arrest, myocardial infarction, stroke, destabilizing psychiatric event such as sustained psychosis and death.

The most common adverse events associated with psilocybin are mild or moderate in severity and include dysphoria, anxiety or panic attacks, negative emotional states, paranoid/delusional thinking during dosing sessions along with increased blood pressure (BP) heart rate, mild nausea and mild headache. These will be discussed with the DMSB quarterly as the study progresses.

Each participant will be monitored at least 3 times the week before and after psilocybin dosing to assess for any adverse events, including C-SRSS testing and vital sign monitoring.

*Participants will be withdrawn if:* 
1. They experience an unexpected or serious adverse event 
2. Depression or suicidal ideation worsens and a higher level of psychiatric care is needed at the discretion of the PI

3.The study team discovers any new information placing the participant at excessive risk if they continue in the study

*The Data and Safety Monitoring Board will be informed of:* 
1. Adverse events, whether expected or unexpected 
2. Study progress: number of participants enrolled, randomized, completed

There will be monthly monitoring by the study team and a formal review by the DSMB quarterly for the duration of the study.

## Storyline Health Data Confidentiality and Security

- The individuals who can view participant videos will only include the participant and the University of Utah and Storyline Health researchers involved in the study. Participant videos will never be shared with individuals outside of the study.
- All study videos will be collected and stored in the Storyline Health Vault Database, which is an ultra-secure, cloud based data storage platform specially designed for protecting participant videos and related data. It has the following technical features that will protect all data for the study:
- HTTPS Communication protocol: All Storyline participant data is transferred and accessed in an encrypted and secure manner. The Storyline Health database API is only available on port 443 via HTTPS and our public websites force HTTPS with HSTS. This is the most secure system available and prevents unauthorized access.
- Access Management: No one else can access participant data other than the participant and researchers involved in the study. Storyline Health ensures this by maintaining robust defense at each layer of the platform to secure data. The features include immutable audit logs, restrictive network rules, and per-record encryption, which all prevent unauthorized access to personal data.
- Encryption / Decryption: All participant records are encrypted with 256-bit AES encryption keys as soon as they enter Storyline Health's infrastructure. Every record is encrypted with a unique initialization vector by a unique encryption key to achieve semantic security. Storyline verifies each record's integrity on a regular basis and on each record request using a hash-based authentication code (HMAC) calculated using its own unique 256-bit HMAC key. Encryption keys, initialization vectors and HMAC keys are re-keyed and each record re-encrypted on a regular basis.
- Network Security: Multiple subsystems combine to power Storyline Health and secure your data. Each subsystem is totally and completely segmented from one another by software and network security rules to maximize protection. Storyline does not store encrypted records and their encryption keys in the same server cluster. Each subsystem can only be accessed by another subsystem via specific network routes and specific inbound and outbound port rules. These features make Storyline’s system ultra-secure for participant data storage.
- Participant videos only exist within the ultra-secure Storyline Health system, where individual participants will have access to their own data and have control over them. The videos will not be stored on their smartphone, tablet or personal computer, or on any other vulnerable device (such as a researcher laptop). The Storyline Health platform ensures that video data is never left on a device that can be lost or stolen.
- Participants will be given ownership of their data. The Storyline Health platform will make it easy for individual participants to view and delete their videos at any time if they choose. If they choose to delete a video, they will be informed about the impact on the study, the importance of their contributions and the major privacy protection measures in place and confidentiality rules governing their data.
- Any facial or speech micro-features extracted from participant videos using artificial intelligence and other data mining approaches will be stored in specially designed, de-identified Storyline Health StoryARC formatted files that maintain participant confidentiality. StoryARC files are text files containing letters and numbers that detail measures extracted from the videos and enable researchers to analyze study participant behavior patterns using mathematical approaches without revealing participant identity or personal information

## Adverse Events and Serious Adverse Events

This study will utilize the CTCAE (NCI Common Terminology Criteria for Adverse Events) Version 5.0 for AE and SAE reporting.

### Adverse Events (AEs)

An adverse event is the appearance or worsening of any undesirable sign, symptom, or medical condition occurring after starting the study drug even if the event is not considered to be related to study drug. For the purposes of this study, the terms toxicity and adverse event are used interchangeably. Medical conditions/diseases present before starting study drug are only considered adverse events if they worsen after starting the study drug. Abnormal laboratory values or test results constitute adverse events only if they induce clinical signs or symptoms, are considered clinically significant, or require therapy.

The collection of adverse events will begin with the dosing of psilocybin and end 14 days after the dose of study drug. However, patients will be monitored for suicide ideation, attempt, and/or occurrence for 28 days following psilocybin administration. There will be no further administrations of study drug during this 28 day monitoring window.

Information about all adverse events, whether volunteered by the subject, discovered by investigator questioning, or detected through physical examination, laboratory test, or other means, will be collected, recorded, and followed as appropriate.

The adverse event should be evaluated to determine:

1. The severity grade based on CTCAE v5.0 (grade 1-5)
2. Its relationship to the study drug(s) (definite, probable, possible, unlikely, not related)
3. Its duration (start and end dates or if continuing at the final exam)
4. Action taken (no action taken; study drug dosage adjusted/temporarily interrupted; study drug permanently discontinued due to this adverse event; concomitant medication taken; non-drug therapy given; hospitalization/prolonged hospitalization)
5. Whether it constitutes an SAE

All adverse events will be treated appropriately. Once an adverse event is detected, it should be followed until its resolution, and assessment should be made at each visit (or more frequently, if necessary) of any changes in severity, the suspected relationship to the study drug, the interventions required to treat it, and the outcome.

Information about common side effects already known about psilocybin are described in the Drug Information (Section 3) and Investigator’s Brochure. This information will be included in the informed consent and will be discussed with the subject during the study as needed.

All adverse events will be recorded in the subject research chart.

### Serious Adverse Event (SAE)

Information about all serious adverse events will be collected and recorded. A serious adverse event is an undesirable sign, symptom, or medical condition which:

- Is fatal or life-threatening
- Results in persistent or significant disability/incapacity
- Is medically significant, i.e., defined as an event that jeopardizes the subject or may require medical or surgical intervention to prevent one of the outcomes listed above
- Causes congenital anomaly or birth defect
- Requires inpatient hospitalization or prolongation of existing hospitalization, unless hospitalization is for:
- Routine treatment or monitoring of the studied indication, not associated with any deterioration in condition (procedures such as central line placements, paracentesis, pain control)
- Elective or pre-planned treatment for a pre-existing condition that is unrelated to the indication under study and has not worsened since the start of study drug
- Treatment on an emergency outpatient basis for an event not fulfilling any of the definitions of an SAE given above and not resulting in hospital admission
- Social reasons and respite care in the absence of any deterioration in the subject’s general condition

The collection of serious adverse events will begin at the time of psilocybin dosing and end 28 days after the dose of study drug.

Any death from any cause while a subject is receiving treatment on this protocol or up to 28 days after the last dose of study drug will be reported. Any death, including suicide, which occurs after that time, which is felt to be treatment-related must be reported as an SAE.

Toxicities that fall within the definitions listed above must be reported as an SAE regardless if they are felt to be treatment-related or not. Toxicities unrelated to treatment that does NOT fall within the definitions above must simply be documented as AEs in the subject research chart.

## SAE Reporting Requirements.

SAEs must be reported to the DSMB, the FDA, the IRB, and Usona according to the requirements described below:

### DSMB Notifications:

A study team member will process and submit the MedWatch 3500A form to the proper DSMB member as necessary. This study team member will summarize and present all reported SAEs according to the Data and Safety Monitoring Board at the quarterly DSMB meeting.

### FDA Notifications:

Adverse events occurring during the course of a clinical study that meet the following criteria will be promptly reported to the FDA:

- Serious
- Unexpected
- Definitely, Probably, or Possibly Related to the investigational drug

Fatal or life-threatening events that meet the criteria above will be reported within 7 calendar days after first knowledge of the event by the investigator; followed by as complete a report as possible within 8 additional calendar days.

All other events that meet the criteria above will be reported within 15 calendar days after the first knowledge of the event by the investigator.

A designated study team member will review the MedWatch report for completeness, accuracy, and applicability to the regulatory reporting requirements. This designated individual will ensure the complete, accurate, and timely reporting of the event to the FDA. The Regulatory Coordinator will submit the report as an amendment to the IND application.

All other adverse events and safety information not requiring expedited reporting that occur or are collected during the course of the study will be summarized and reported to the FDA through the IND Annual Report.

### IRB Notifications:

Events meeting the University of Utah IRB or local IRB reporting requirements will be submitted per local guidelines.

### Usona Notifications:

SAEs must be reported to Usona within one business day of first knowledge of the event. Reports should be emailed to:

Email: [usonaSAE@usonainstitute.org](mailto:usonaSAE@usonainstitute.org)

# Minimum data to be reported via Usona forms for SAEs.

-Date of Report – Initial Report or Follow-up Report

-Protocol ID, Patient ID, Patient Information

-Serious Adverse Event Term

-Protocol Defined Severity

-Onset Date, End Date

-Relationship to Study Drug

-Action taken toward Study Drug

-Outcome, If Recovered with Sequalae, elaborate in narrative

-Seriousness Criteria / Reason for Report

-Patient Narrative

**Pregnancy**

# Initial report

-Gravidity

-Parity

-Estimated delivery date

-Prenatal care (including initiation relevant to gestation)

-Pregnancy complications and dates

-Medications used during pregnancy

# Follow-up report: Delivery

-Gravidity

-Parity

-Estimated delivery date

-Pregnancy outcome

-Duration of labor

-Type of delivery

-Postpartum complications

-Infant birth weight

-Apgar scores (1 min, 5 min)

-NICU admission

-Complications of newborn during hospitalization

-Date of hospital discharge or death

-Birth defects noted at birth

**Follow-up report: Well Child Visit**

-Summary of diagnostic tests

-Complications

-Updates on past findings/anomalies

**Annual Reporting:**

The PI must report data to Usona by February 5^th^ of each year during the study. Each report shall contain data from January 22 of the prior year through January 21 of the current year. This data includes dosing information, adverse events (AEs), concomitant medications, serious adverse events (SAEs), and pregnancy. This data set is also reported within 30 days of end of study.

## Reporting of Pregnancy

Although pregnancy is not considered an adverse event, any exposure to the investigational products during pregnancy or breastfeeding must be reported promptly. Exposure may occur by a woman actively receiving study therapy. Any possible pregnancy or breastfeeding exposure during study therapy and up to 30 days after the last dose of psilocybin must be reported within one business day of awareness regardless of the occurrence of an SAE. Should a woman on study therapy become pregnant, she should immediately discontinue study treatment.

Women exposed to IP during pregnancy or while breastfeeding will be followed for pregnancy outcome and neonate health. Pregnancy outcomes may meet criteria as an SAE if ectopic pregnancy, spontaneous abortion, intrauterine fetal demise, neonatal death, or congenital anomaly occurs. Congenital anomalies that occur in a live-born baby, a terminated fetus, an intrauterine fetal demise, or a neonatal death should be reported as an SAE. Any neonatal deaths that occur up to 30 days after birth or breastfeeding exposure should be reported as an SAE. All live births must be followed for a minimum of 30 days or to the first well-baby visit. Further follow-up on birth outcomes and neonate health will be handled on a case-by-case base.

## Protocol Amendments

Any amendments or administrative changes to an IRB approved protocol will not be initiated without submission of an amendment for IRB review and approval.

These requirements for approval will in no way prevent any immediate action from being taken by the investigator in the interests of preserving the safety of all subjects included in the trial.

Any amendments to the protocol that significantly affect the safety of subjects, the scope of the investigation, or the scientific quality of the study are required to submit the amendment for FDA review.

## Protocol Deviations

A protocol deviation (or violation) is any departure from the defined procedures and treatment plans as outlined in the protocol version submitted and previously approved by the IRB. Protocol deviations have the potential to place participants at risk and can also undermine the scientific integrity of the study thus jeopardizing the justification for the research. Protocol deviations are unplanned and unintentional events.

Because some protocol deviations pose no conceivable threat to participant safety or scientific integrity, reporting is left to the discretion of the PI within the context of the guidelines below. The sponsor requires the **prompt reporting** to RCO of protocol deviations which are:

- Exceptions to eligibility criteria.
- Intended to eliminate an apparent immediate hazard to a research participant or
- Harmful (caused harm to participants or others, or place them at increased risk of harm - including physical, psychological, economic, or social harm), or
- Possible serious or continued noncompliance

## FDA Annual Reporting

An annual progress report will be submitted to the FDA within 60 days of the anniversary of the date that the IND went into effect. (21 CFR 312.33).

## DEA Registration and Reporting

Before study initiation, appropriate registration and licensing will be gained through the DEA in compliance with 21 CRF Part 1301 for the investigational use of a controlled substance. Any theft or significant loss of IP will be reported to the DEA on DEA Form 106 within one business day of first knowledge. If over the course of the investigation, it is deemed necessary to increase the amount of Schedule I product, the DEA registration will be modified per 21 CRF Part 1301.51 before the initiation of the protocol amendment.

## Clinical Trials Data Bank

The study will be registered on [**http://clinicaltrials.gov**](http://clinicaltrials.gov)**.**

## Record Keeping

Per 21 CFR 312.57, the Investigator records shall be maintained for a period of 2 years following the date a marketing application is approved; or, if no application is filed or the application is not approved, until 2 years after the investigation is discontinued and the FDA is notified.

# BIBLIOGRAPHY

## Key References

Agin-Liebes, G. I., Malone, T., Yalch, M. M., Mennenga, S. E., Ponté, K. L., Guss, J., Bossis, A. P., Grigsby, J., Fischer, S., & Ross, S. (2020). Long-term follow-up of psilocybin-assisted psychotherapy for psychiatric and existential distress in patients with life-threatening cancer. Journal of Psychopharmacology, 34(2), 155–166. https://doi.org/10.1177/0269881119897615

Ameli, R, Sinaii, N, West, C, Luna, M, Panahi, S. (2020) Effect of a brief mindfulness-based program on stress in healthcare professionals at a US biomedical research hospital: a randomized clinical trial. JAMA Network Open; 3(8) e2013424. doi:10.1001/jamanetworkopen.2020.13424

Blanchard, E. B., Jones-Alexander, J., Buckley, T. C. & Forneris, C. A. Psychometric properties of the PTSD Checklist (PCL). Behav Res Ther 34, 669–673 (1996).

Bogenschutz, M. P., Forcehimes, A. A., Pommy, J. A., Wilcox, C. E., Barbosa, P., & Strassman, R. J. (2015). Psilocybin-assisted treatment for alcohol dependence: A proof-of-concept study. Journal of Psychopharmacology, 29(3), 289–299. https://doi.org/10.1177/0269881114565144

Brady, K. Ni, P, Sheldrick R, Trockel, M, Shanafelt, T. (2020) Describing the emotional exhaustion, depersonalization and low personal accomplishment symptoms associated with Maslach Burnout Inventory subscale scores in US physicians: an item response theory analysis. J Patient Reported Outcomes, 4 (42) <https://doi.org/10.1186/s41687-020-00204-x>

Carhart-Harris, R. L. (2018). The entropic brain - revisited. In Neuropharmacology (Vol. 142, pp. 167–178). Elsevier Ltd. https://doi.org/10.1016/j.neuropharm.2018.03.010

Carhart-Harris, R. L., & Friston, K. J. (2019). REBUS and the anarchic brain: Toward a unified model of the brain action of psychedelics. Pharmacological Reviews, 71(3), 316–344. https://doi.org/10.1124/pr.118.017160

Carhart-Harris, R. L., Leech, R., Hellyer, P. J., Shanahan, M., Feilding, A., Tagliazucchi, E., Chialvo, D. R., & Nutt, D. (2014). The entropic brain: A theory of conscious states informed by neuroimaging research with psychedelic drugs. Frontiers in Human Neuroscience, 8(1 FEB), 20. https://doi.org/10.3389/fnhum.2014.00020

Cohen-Katz J, Wiley SD, Capuano T, Baker DM, Kimmel S, Shapiro S. The effects of mindfulness-based stress reduction on nurse stress and burnout, Part II: A quantitative and qualitative study. Holist Nurs Pract. 2005 Feb;19(1):26–35.

Davis, A. K., Barrett, F. S., May, D. G., Cosimano, M. P., Sepeda, N. D., Johnson, M. W., Finan, P. H., & Griffiths, R. R. (2021). Effects of Psilocybin-Assisted Therapy on Major Depressive Disorder: A Randomized Clinical Trial. JAMA Psychiatry, 78(5), 481–489. <https://doi.org/10.1001/jamapsychiatry.2020.3285>

Delgadillo, J. et al. Brief case finding tools for anxiety disorders: validation of GAD-7 and GAD-2 in addictions treatment. Drug Alcohol Depend 125, 37–42 (2012).

Fendel, J, Burkle, J, Goritz, A. (2019) Mindfulness-based interventions to reduce burnout and stress in physicians: a study protocol for a systeemaatiic review and meta-analysis. BMJ Open 2019;9:e032295. doi:10.1136/bmjopen-2019-032295ew and meta-analysis.

Gazelle, G., Liebschutz, J. M., & Riess, H. (2015). Physician Burnout: Coaching a Way Out. Journal of General Internal Medicine, 30(4), 508–513. https://doi.org/10.1007/s11606-014-3144-y

Goldberg SB, Pace BT, Nicholas CR, Raison CL, Hutson PR. The experimental effects of psilocybin on symptoms of anxiety and depression: a meta-analysis. Psychiatry Res 2020; 284-112749.

Griffiths, R. R., Johnson, M. W., Carducci, M. A., Umbricht, A., Richards, W. A., Richards, B. D., Cosimano, M. P., & Klinedinst, M. A. (2016). Psilocybin produces substantial and sustained decreases in depression and anxiety in patients with life-threatening cancer: A randomized double-blind trial. Journal of Psychopharmacology, 30(12), 1181–1197. <https://doi.org/10.1177/0269881116675513>

Griffiths, R. R., Johnson, M. W., Richards, W. A., Richards, B. D., Jesse, R., MacLean, K. A., Barrett, F. S., Cosimano, M. P., & Klinedinst, M. A. (2018). Psilocybin-occasioned mystical-type experience in combination with meditation and other spiritual practices produces enduring positive changes in psychological functioning and in trait measures of prosocial attitudes and behaviors. Journal of Psychopharmacology, 32(1), 49–69. <https://doi.org/10.1177/0269881117731279>

Griffiths, R. R., Richards, W. A., Mccann, U., & Jesse, R. (2006). Psilocybin can occasion mystical-type experiences having substantial and sustained personal meaning and spiritual significance. Psychopharmacology, 187(3), 268–283. <https://doi.org/10.1007/s00213-006-0457-5>

Grob, C. S., Bossis, A. P., & Griffiths, R. R. (2013). Use of the classic hallucinogen psilocybin for treatment of existential distress associated with cancer. In Psychological Aspects of Cancer: A Guide to Emotional and Psychological Consequences of Cancer, Their Causes and Their Management (Vol. 9781461448662, pp. 291–308). Springer US. <https://doi.org/10.1007/978-1-4614-4866-2_17>

Han, S., Shanafelt, T. D., Sinsky, C. A., Awad, K. M., Dyrbye, L. N., Fiscus, L. C., Trockel, M., & Goh, J. (2019). Estimating the attributable cost of physician burnout in the United States. Annals of Internal Medicine, 170(11), 784–790. https://doi.org/10.7326/M18-1422

Hanley, A. W., Nakamura, Y., & Garland, E. L. (2018). The Nondual Awareness Dimensional Assessment (NADA): New tools to assess nondual traits and states of consciousness occurring within and beyond the context of meditation. Psychological Assessment, 30(12), 1625–1639. <https://doi.org/10.1037/pas0000615>

Hartzband, P., & Groopman, J. (2020). Physician Burnout, Interrupted. New England Journal of Medicine, 382(26), 2485–2487. https://doi.org/10.1056/nejmp2003149

Johnson, M. W., Garcia-Romeu, A., & Griffiths, R. R. (2017). Long-term follow-up of psilocybin-facilitated smoking cessation. American Journal of Drug and Alcohol Abuse, 43(1), 55–60. <https://doi.org/10.3109/00952990.2016.1170135>

Kabat-Zinn J (2013). Full Catastrophe Living: Using the Wisdom of Your Body and Mind to Face Stress, Pain, and Illness. New York: Bantam Dell. [ISBN](https://wiki2.org/en/ISBN_(identifier)) [978-0345539724](https://wiki2.org/en/Special:BookSources/978-0345539724).

Khoury B, Lecomte T, Fortin G, Masse M, Therien P, Bouchard V, et al. Mindfulness-based therapy: a comprehensive meta-analysis. Clin Psychol Rev. 2013 Aug;33(6):763–71.

Khoury B, Sharma M, Rush S, Fournier C. (2015) Mindfulness-based stress reduction for healthy individuals: a metaanalysis. J Psychosomatic Research, 78: 519-528

Löwe, B., Kroenke, K. & Gräfe, K. Detecting and monitoring depression with a two-item questionnaire (PHQ-2). J Psychosom Res 58, 163–171 (2005).

MacLean, K. A., Johnson, M. W., & Griffiths, R. R. (2011). Mystical experiences occasioned by the hallucinogen psilocybin lead to increases in the personality domain of openness. Journal of Psychopharmacology, 25(11), 1453–1461. <https://doi.org/10.1177/0269881111420188>

Maslach, C., & Leiter, M. P. (2016). Understanding the burnout experience: Recent research and its implications for psychiatry. World Psychiatry, 15(2), 103–111. https://doi.org/10.1002/wps.20311

Maslach, C., Leiter, M. P., & Jackson, S. E. (2012). Making a significant difference with burnout interventions: Researcher and practitioner collaboration. Journal of Organizational Behavior, 33(2), 296–300. https://doi.org/10.1002/job.784

Moreno, F. A., Wiegand, C. B., Taitano, E. K., & Delgado, P. L. (2006). Safety, tolerability, and efficacy of psilocybin in 9 patients with obsessive-compulsive disorder. Journal of Clinical Psychiatry, 67(11), 1735–1740. <https://doi.org/10.4088/JCP.v67n1110>

Nagpal, A., and Singh, V. (2019). Feature selection from high dimensional data based on iterative qualitative mutual information. J Intell Fuzzy Syst 36, 5845–5856.

Nichols, David. (2016) Psychedelics. Pharmacological Reviews 68: 264-355

Panagioti, M., Geraghty, K., Johnson, J., Zhou, A., Panagopoulou, E., Chew-Graham, C., Peters, D., Hodkinson, A., Riley, R., & Esmail, A. (2018). Association Between Physician Burnout and Patient Safety, Professionalism, and Patient Satisfaction. JAMA Internal Medicine, 178(10), 1317. <https://doi.org/10.1001/jamainternmed.2018.3713>

Payne, J, Chambers, R, Liknaitzky P. (2021) Combining psychedelic and mindfulness interventions: synergies to inform clinical practice. ACS Pharmacol. Transl. Sci. 4,2, 416-423. <https://doi.org/10.1021/acsptsci.1c00034>

Pokorny T, Preller KH, Kometer M, Dziobek I, Vollenwider FX. (2017) Effect of psilocybin on empathy and moral decision-making. International J of Neuropsychopharmacology, 20(9) 747-57

Preacher, K. J., & Hayes, A. F. (2008). Asymptotic and resampling strategies for assessing and comparing indirect effects in multiple mediator models. Behavior research methods, 40(3), 879-891.

Ross, S., Bossis, A., Guss, J., Agin-Liebes, G., Malone, T., Cohen, B., Mennenga, S. E., Belser, A., Kalliontzi, K., Babb, J., Su, Z., Corby, P., & Schmidt, B. L. (2016). Rapid and sustained symptom reduction following psilocybin treatment for anxiety and depression in patients with life-threatening cancer: a randomized controlled trial. Journal of Psychopharmacology, 30(12), 1165–1180. <https://doi.org/10.1177/0269881116675512>

Salvado, M, Marques, D, Pires, I, Silva, N. (2021) Mindfulness-based interventions to reduce burnout in primary healthcare professionals: a systematic review and meta-analysis. Healthcare, 9 1342 https://doi.org/10.3390/healthcare9101342

Shanafelt, T. D., Balch, C. M., Dyrbye, L., Bechamps, G., Russell, T., Satele, D., Rummans, T., Swartz, K., Novotny, P. J., Sloan, J., & Oreskovich, M. R. (2011). Special report: Suicidal ideation among American surgeons. Archives of Surgery, 146(1), 54–62. https://doi.org/10.1001/archsurg.2010.292

Shear, M. K., Vander Bilt, J., Rucci, P., Endicott, J., Lydiard, B., Otto, M. W., Pollack, M. H., Chandler, L., Williams, J., Ali, A., & Frank, D. M. (2001). Reliability and validity of a structured interview guide for the Hamilton Anxiety Rating Scale (SIGH-A). Depression and Anxiety, 13(4), 166–178. <https://doi.org/10.1002/da.1033>

West, C. P., Tan, A. D., & Shanafelt, T. D. (2012). Association of resident fatigue and distress with occupational blood and body fluid exposures and motor vehicle incidents. Mayo Clinic Proceedings, 87(12), 1138–1144. <https://doi.org/10.1016/j.mayocp.2012.07.021>

##### Storyline Assessments

## Assessment Components

Neurological Assessment

A clinical neurological assessment developed by Dr. Fanny Elahi, a clinical neurologist at Mt Sinai Hospital, NY. Patients perform each assessment by following an instructional video on their phone. It takes ~5 minutes to complete.

IntroNeuro.mp4 *- Introduction to Neurological assessment*

Facial movement *- Interactive video assessment*

0minutes21seconds

Eye movement *- Interactive video assessment*

0minutes29seconds

Opposite Eye Movement *- Interactive video assessment*

0minutes30seconds

Speech *- Interactive video assessment*

0minutes27seconds

Repeat after me… *- Interactive video assessment*

0minutes30seconds

Count backwards *- Interactive video assessment*

0minutes22seconds

Hand Movements *- Interactive video assessment*

0minutes47seconds

Finger movements *- Interactive video assessment*

0minutes30seconds

Finger Tapping *- Interactive video assessment*

0minutes41seconds

Finger Accuracy *- Interactive video assessment*

0minutes42seconds

Draw a smiley face on the screen using your mouse or fingertip, then click continue. *- Drawing question*

Memorize the image in as much detail as you can for 10 seconds

0minutes10seconds

Now, draw the previous image with as much detail and accuracy as you can. Click save when done. *- Drawing question*

Using your mouse pad or on your screen, draw a round clock and fill in the numbers for the hours. Then add the clock hands to show a time of 10 minutes after 11. *- Drawing question*

You are all finished with this part {firstName}! That was great. Thank you!

Psychiatric and Wellness Assessment

Please talk about how you have you been feeling physically and mentally over the past couple of weeks? Any health problems or concerns? Take your time. - *Video question*

3minutes0seconds

Please talk about what you are most worried about in your life right now? - *Video question*

3minutes0seconds

How much social support do you have in your life? How do other people treat you? Are you lonely? - *Video question*

3minutes0seconds

Click start and name as many animals as you can in the next minute. (It will automatically stop after 1 minute.) - *Video question*

1minute0seconds

#### The Caterpillar Question

1 question

Make up and tell me the most interesting story you can about a caterpillar. - *Video question*

3minutes0seconds

Psychological Assessment

#### Storyline Association - 4 Question Set

4 questions

Describe how this image makes you feel *- Mountain image*

1minute0seconds

Describe how this image makes you feel. *- Dead Body image*

1minute0seconds

Describe how this image makes you feel. *- Sick dog image*

1minute0seconds

Describe how this image makes you feel. *- Happy puppy image*

1minute0seconds

Clinical Questionnaires

#### Storyline ASQ-5 + Video v2

7 questions

In the past few weeks, have you wished you were dead?

Unlimited

In the past few weeks, have you felt that you or your family would be better off if you were dead?

Unlimited

In the past week, have you been having thoughts of killing yourself?

Unlimited

Have you ever tried to kill yourself?

Unlimited

Skip 1

Please describe how and when you tried to kill yourself.

3minutes0seconds

Are you having thoughts of killing yourself right now?

Unlimited

Skip 1

How serious are you about committing suicide? Please describe your plans to kill yourself.

3minutes0seconds

#### PCL-C Questionnaire

In the past month, how much were you bothered by:

9 Questions

#### PHQ-9 Questionnaire

Over the last 2 weeks, how often have they been bothered by the following problems?

9 Questions

#### GAD-7 Questionnaire

Over the last 2 weeks, how often have you been bothered by the following problems?

7 questions

#### Basic Neurological Symptoms  Questionnaire

12 questions

#### Mental Health and Addiction Symptoms Questions

Have you had any of these symptoms recently?

Unlimited

Have you ever been diagnosed with any addiction, behavioral, or mental illness?

Unlimited

Skip 1

Which of the following have you been diagnosed with? (Select all that apply.)

Unlimited

List all legally prescribed medications are you taking.

Unlimited

Do you commonly use any of the following? (Select all that apply)

Unlimited

Have you used any of the following in the last 24 hours? (Select all that apply)

Unlimited

List any drugs or substances have you taken in the last 24 hours.

Unlimited

How easy was this online assessment for you to do? (0- very difficult, 10- very easy)

Unlimited

Please share any difficulties you encountered or recommendations to improve our platform.

Unlimited

**All done. Thank you.**

##### Quick Inventory of Depressive Symptomatology (Self-Report) (QIDS-SR16)

NAME: TODAY’S DATE:

Please circle the one response to each item that best describes you for the past seven days.

1. Falling Asleep:
   1. I never take longer than 30 minutes to fall asleep.
   2. I take at least 30 minutes to fall asleep, less than half the time. 2 I take at least 30 minutes to fall asleep, more than half the time.

3 I take more than 60 minutes to fall asleep, more than half the time.

1. Sleep During the Night:
2. I do not wake up at night.
3. I have a restless, light sleep with a few brief awakenings each night. 2 I wake up at least once a night, but I go back to sleep easily.

3 I awaken more than once a night and stay awake for 20 minutes or more, more than half the time.

1. Waking Up Too Early:

0 Most of the time, I awaken no more than 30 minutes before I need to get up. 1 More than half the time, I awaken more than 30 minutes before I need to get up.

1. I almost always awaken at least one hour or so before I need to, but I go back to sleep eventually.
2. I awaken at least one hour before I need to, and can’t go back to sleep.
3. Sleeping Too Much:

0 I sleep no longer than 7–8 hours/night, without napping during the day. 1 I sleep no longer than 10 hours in a 24-hour period including naps.

2 I sleep no longer than 12 hours in a 24-hour period including naps. 3 I sleep longer than 12 hours in a 24-hour period including naps.

Enter the highest score on any 1 of the 4 sleep items (1–4 above)

1. Feeling Sad:
2. I do not feel sad
3. I feel sad less than half the time. 2 I feel sad more than half the time. 3 I feel sad nearly all of the time.
4. Decreased Appetite:
5. There is no change in my usual appetite.
6. I eat somewhat less often or lesser amounts of food than usual. 2 I eat much less than usual and only with personal effort.

3 I rarely eat within a 24-hour period, and only with extreme personal effort or when others persuade me to eat.

1. Increased Appetite:
2. There is no change from my usual appetite.
3. I feel a need to eat more frequently than usual.
4. I regularly eat more often and/or greater amounts of food than usual. 3 I feel driven to overeat both at mealtime and between meals.
5. Decreased Weight (Within the Last Two Weeks): 0 I have not had a change in my weight.

1 I feel as if I’ve had a slight weight loss. 2 I have lost 2 pounds or more.

3 I have lost 5 pounds or more.

1. Increased Weight (Within the Last Two Weeks): 0 I have not had a change in my weight.

1 I feel as if I’ve had a slight weight gain. 2 I have gained 2 pounds or more.

3 I have gained 5 pounds or more.

Enter the highest score on any 1 of the 4 appetite/weight change items (6–9 above)

1. Concentration/Decision Making:

0 There is no change in my usual capacity to concentrate or make decisions. 1 I occasionally feel indecisive or find that my attention wanders.

2 Most of the time, I struggle to focus my attention or to make decisions. 3 I cannot concentrate well enough to read or cannot make even minor decisions.

1. View of Myself:

0 I see myself as equally worthwhile and deserving as other people. 1 I am more self-blaming than usual.

1. I largely believe that I cause problems for others.
2. I think almost constantly about major and minor defects in myself.
3. Thoughts of Death or Suicide:
4. I do not think of suicide or death.
5. I feel that life is empty or wonder if it’s worth living.
6. I think of suicide or death several times a week for several minutes.
7. I think of suicide or death several times a day in some detail, or I have made specific plans for suicide or have actually tried to take my life.
8. General Interest:
9. There is no change from usual in how interested I am in other people or activities.
10. I notice that I am less interested in people or activities.
11. I find I have interest in only one or two of my formerly pursued activities. 3 I have virtually no interest in formerly pursued activities.
12. Energy Level:

0 There is no change in my usual level of energy. 1 I get tired more easily than usual.

1. I have to make a big effort to start or finish my usual daily activities (for example, shopping, homework, cooking or going to work).
2. I really cannot carry out most of my usual daily activities because I just don’t have the energy.
3. Feeling Slowed Down:
4. I think, speak, and move at my usual rate of speed.
5. I find that my thinking is slowed down or my voice sounds dull or flat
6. It takes me several seconds to respond to most questions and I’m sure my thinking is slowed.
7. I am often unable to respond to questions without extreme effort.
8. Feeling Restless:
9. I do not feel restless.
10. I’m often fidgety, wringing my hands, or need to shift how I am sitting. 2 I have impulses to move about and am quite restless.

3 At times, I am unable to stay seated and need to pace around.

Enter the highest score on either of the 2 psychomotor items (15 or 16 above)

**Total Score: (Range 0–27)**

##### MBI Human Services Survey for Medical Personnel

| **How often:** | **0** | **1** | **2** | **3** | **4** | **5** | **6** |
| --- | --- | --- | --- | --- | --- | --- | --- |
|  | Never | A few times a year or less | Once a month or less | A few times a month | Once a week | A few times a week | Every day |

| **How often ( 0-6)** | **Statements:** |
| --- | --- |
| 1. | I feel emotionally drained from my work. |
| 2. | I feel used up at the end of the workday. |
| 3. | I feel fatigued when I get up in the morning and have to face another day on the job. |
| 4. | I can easily understand how my patients feel about things. |
| 5. | I feel I treat some patients as if they were impersonal objects. |
| 6. | Working with people all day is really a strain for me. |
| 7. | I deal very effectively with the problems of my patients. |
| 8. | I feel burned out from my work. |
| 9. | I feel I'm positively influencing other people's lives through my work. |
| 10. | I've become more callous toward people since I took this job. |
| 11. | I worry that this job is hardening me emotionally. |
| 12. | I feel very energetic. |
| 13. | I feel frustrated by my job. |
| 14. | I feel I'm working too hard on my job. |
| 15. | I don't really care what happens to some patients. |
| 16. | Working with people directly puts too much stress on me. |
| 17. | I can easily create a relaxed atmosphere with my patients. |
| 18. | I feel exhilarated after working closely with my patients. |
| 19. | I have accomplished many worthwhile things in this job. |
| 20. | I feel like I'm at the end of my rope. |
| 21. | In my work, I deal with emotional problems very calmly. |
| 22. | I feel patients blame me for some of their problems. |

(Administrative use only)

EE Total score: ___ DP Total score: PA Total score:

##### Demoralization Scale-II (DS-II)


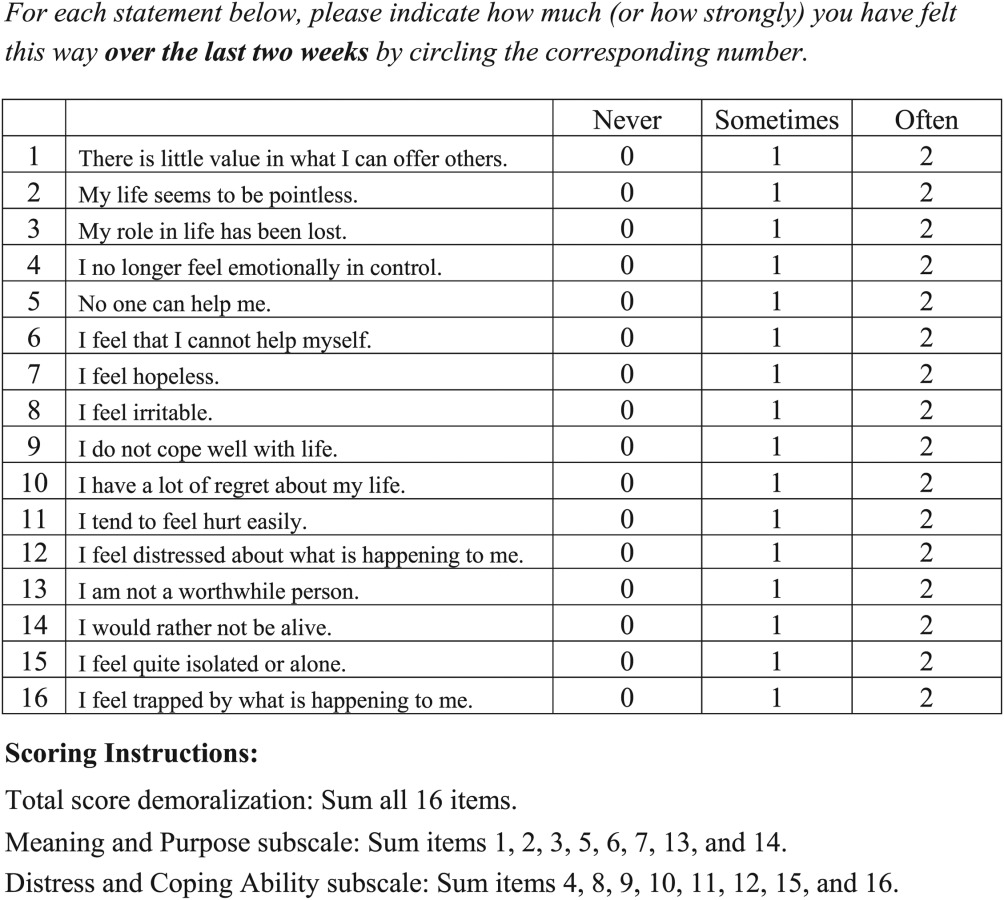


#####
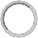

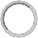

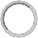

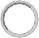

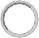

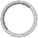

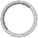

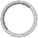

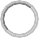

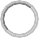

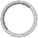

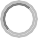

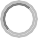

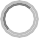

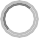

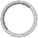

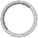

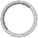

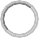

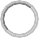

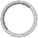

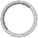

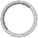

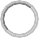

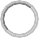

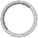

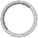

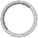

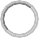

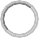

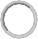

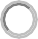

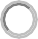

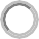

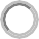

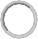

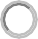

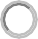

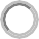

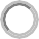

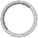

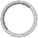

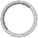

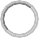

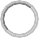

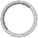

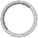

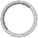

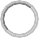

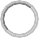

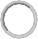

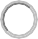

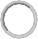

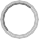

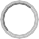

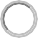

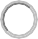

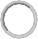

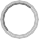

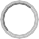

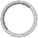

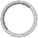

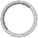

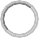

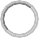

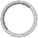

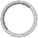

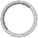

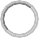

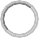

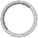

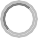

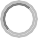

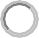

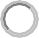

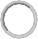

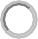

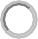

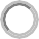

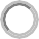

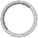

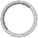

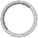

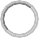

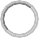
PCL-5


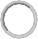

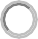

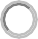

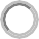

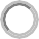
**Instructions:** Below is a list of problems that people sometimes have in response to a very stressful experience. Please read each problem carefully and then circle one of the numbers to the right to indicate how much you have been bothered by that problem in the past month.

| **In the past month, how much were you bothered by:** | **Not at all** | **A little bit** | **Moderately** | **Quite a bit** | **Extremely** |
| --- | --- | --- | --- | --- | --- |
| 1. Repeated, disturbing, and unwanted memories of the stressful experience? | 0 | 1 | 2 | 3 | 4 |
| 2. Repeated, disturbing dreams of the stressful experience? | 0 | 1 | 2 | 3 | 4 |
| 3. Suddenly feeling or acting as if the stressful experience were actually happening again (as if you were actually back there reliving it)? | 0 | 1 | 2 | 3 | 4 |
| 4. Feeling very upset when something reminded you of the stressful experience? | 0 | 1 | 2 | 3 | 4 |
| 5. Having strong physical reactions when something reminded you of the stressful experience (for example, heart pounding, trouble breathing, sweating)? | 0 | 1 | 2 | 3 | 4 |
| 6. Avoiding memories, thoughts, or feelings related to the stressful experience? | 0 | 1 | 2 | 3 | 4 |
| 7. Avoiding external reminders of the stressful experience (for example, people, places, conversations, activities, objects, or situations)? | 0 | 1 | 2 | 3 | 4 |
| 8. Trouble remembering important parts of the stressful experience? | 0 | 1 | 2 | 3 | 4 |
| 9. Having strong negative beliefs about yourself, other people, or the world (for example, having thoughts such as: I am bad, there is something seriously wrong with me,  no one can be trusted, the world is completely dangerous)? | 0 | 1 | 2 | 3 | 4 |
| 10. Blaming yourself or someone else for the stressful experience or what happened after it? | 0 | 1 | 2 | 3 | 4 |
| 11. Having strong negative feelings such as fear, horror, anger, guilt, or shame? | 0 | 1 | 2 | 3 | 4 |
| 12. Loss of interest in activities that you used to enjoy? | 0 | 1 | 2 | 3 | 4 |
| 13. Feeling distant or cut off from other people? | 0 | 1 | 2 | 3 | 4 |
| 14. Trouble experiencing positive feelings (for example, being unable to feel happiness or have loving feelings for people close to you)? | 0 | 1 | 2 | 3 | 4 |
| 15. Irritable behavior, angry outbursts, or acting aggressively? | 0 | 1 | 2 | 3 | 4 |
| 16. Taking too many risks or doing things that could cause you harm? | 0 | 1 | 2 | 3 | 4 |
| 17. Being “superalert” or watchful or on guard? | 0 | 1 | 2 | 3 | 4 |
| 18. Feeling jumpy or easily startled? | 0 | 1 | 2 | 3 | 4 |
| 19. Having difficulty concentrating? | 0 | 1 | 2 | 3 | 4 |
| 20. Trouble falling or staying asleep? | 0 | 1 | 2 | 3 | 4 |

**PCL-5 (**11 April 2018) National Center for PTSD Page 1 of 1

##### FFMQ-15

**Subject number_________** **Date_________**

**Please rate each of the following statements using the scale provided.  Write the number in the blank that best describes your own opinion of what is generally true for you.**

| **1** | **2** | **3** | **4** | **5** |
| --- | --- | --- | --- | --- |
| **never or very rarely true** | **rarely**  **true** | **sometimes**  **true** | **often**  **true** | **very often or always true** |

_____1.  When I take a shower or a bath, I stay alert to the sensations of water on my body.

_____2. I’m good at finding words to describe my feelings.

_____3. I don’t pay attention to what I’m doing because I’m daydreaming, worrying, or

otherwise distracted.

_____4. I believe some of my thoughts are abnormal or bad and I shouldn’t think that way.

_____5. When I have distressing thoughts or images, I “step back” and am aware of the thought

 or image without getting taken over by it.

_____6. I notice how foods and drinks affect my thoughts, bodily sensations, and emotions.

_____7. I have trouble thinking of the right words to express how I feel about things.

_____8. I do jobs or tasks automatically without being aware of what I’m doing.

_____9. I think some of my emotions are bad or inappropriate and I shouldn’t feel them.

_____10. When I have distressing thoughts or images I am able just to notice them without

 reacting.

_____11. I pay attention to sensations, such as the wind in my hair or sun on my face.

_____12. Even when I’m feeling terribly upset I can find a way to put it into words.

_____13. I find myself doing things without paying attention.

_____14. I tell myself I shouldn’t be feeling the way I’m feeling.

_____15. When I have distressing thoughts or images I just notice them and let them go.

##### TMS

| **Instructions:** We are interested in what you just experienced. Below is a list of things that people sometimes experience. Please read each statement. Next to each statement are five choices: “not at all,” “a little,” “moderately,” “quite a bit,” and “very much.” Please indicate the extent to which you agree with each statement. In other words, how well does the statement describe what you just experienced, just now? | **Not at all** | **A little** | **Moderately** | **Quite a bit** | **Very much** |
| --- | --- | --- | --- | --- | --- |
| 1. I experienced myself as separate from my changing thoughts and feelings. | 0 | 1 | 2 | 3 | 4 |
| 1. I was more concerned with being open to my experiences than controlling or changing them. | 0 | 1 | 2 | 3 | 4 |
| 1. I was curious about what I might learn about myself by taking notice of how I react to certain thoughts, feelings or sensations. | 0 | 1 | 2 | 3 | 4 |
| 1. I experienced my thoughts more as events in my mind than as a necessarily accurate reflection of the way things ‘really’ are. | 0 | 1 | 2 | 3 | 4 |
| 1. I was curious to see what my mind was up to from moment to moment. | 0 | 1 | 2 | 3 | 4 |
| 1. I was curious about each of the thoughts and feelings that I was having. | 0 | 1 | 2 | 3 | 4 |
| 1. I was receptive to observing unpleasant thoughts and feelings without interfering with them. | 0 | 1 | 2 | 3 | 4 |
| 1. I was more invested in just watching my experiences as they arose, than in figuring out what they could mean. | 0 | 1 | 2 | 3 | 4 |
| 1. I approach each experience by trying to accept it, no matter whether it was pleasant or unpleasant. | 0 | 1 | 2 | 3 | 4 |
| **Instructions:** We are interested in what you just experienced. Below is a list of things that people sometimes experience. Please read each statement. Next to each statement are five choices: “not at all,” “a little,” “moderately,” “quite a bit,” and “very much.” Please indicate the extent to which you agree with each statement. In other words, how well does the statement describe what you just experienced, just now? | **Not at all** | **A little** | **Moderately** | **Quite a bit** | **Very much** |
| 1. I remained curious about the nature of each experience as it arose. | 0 | 1 | 2 | 3 | 4 |
| 1. I was aware of my thoughts and feelings without overidentifying with them. | 0 | 1 | 2 | 3 | 4 |
| 1. I was curious about my reactions to things. | 0 | 1 | 2 | 3 | 4 |
| 1. I was curious about what I might learn about myself by just taking notice of what my attention gets drawn to. | 0 | 1 | 2 | 3 | 4 |

##### Nondual Awareness Dimensional Assessment (NADA) (trait)

Looking back on your lifetime, please rate the degree to which at any time during your life you experienced the following phenomena. Answer each question according to your feelings, thoughts, and experiences at the time.

|  | Never or Very Rarely | Rarely | Sometimes | Often | Very Often or Always |
| --- | --- | --- | --- | --- | --- |
| I have had an experience in which I felt myself to be absorbed as one with all things |  |  |  |  |  |
| I have had an experience in which the boundaries of my self dissolved |  |  |  |  |  |
| I have had an experience in which all things seemed to be unified into a single whole |  |  |  |  |  |
| I have had an experience in which my mind expanded into space |  |  |  |  |  |
| I have experienced all notion of self and identity dissolve away |  |  |  |  |  |
| I have experienced the insight that “all is One” |  |  |  |  |  |
| I have experienced a feeling of oneness in which the boundaries between what is me and what is not me has dissolved |  |  |  |  |  |
| I have experienced a melting or merging with the others; I became others and they became me |  |  |  |  |  |
| It has seemed to me that my environment and I were one |  |  |  |  |  |
| I have experienced an all-embracing love |  |  |  |  |  |
| I have been surrounded and filled with a blissful warmth or energy |  |  |  |  |  |
| I have felt a sense of awe and wonder |  |  |  |  |  |
| I have experienced a perfectly peaceful state |  |  |  |  |  |

___________________________________________ __________________

Signature Date

| **NONDUAL AWARENESS DIMENSIONAL ASSESSMENT – STATE** | | | | | | | | | | | |
| --- | --- | --- | --- | --- | --- | --- | --- | --- | --- | --- | --- |
| *Please read each statement and indicate the extent to which you agree with each statement. In other words, how well does the statement describe what you just experienced, just now?* | **Not at all** | | | | |  | **Very much** | | | | |
| 1. I experienced all things seeming to unify into a single whole. | 0 | 1 | 2 | 3 | 4 | 5 | 6 | 7 | 8 | 9 | 10 |
| 2. I experienced all sense of self and identity dissolve away. | 0 | 1 | 2 | 3 | 4 | 5 | 6 | 7 | 8 | 9 | 10 |
| 3. I felt surrounded and filled with a blissful warmth or energy. | 0 | 1 | 2 | 3 | 4 | 5 | 6 | 7 | 8 | 9 | 10 |

**NADA-S SCORING**

Full Scale Score: Average all items.

|  | | | | | | |
| --- | --- | --- | --- | --- | --- | --- |
|  | |  |  |  |  | |
|  | | |  |  | | |
|  |  |  |  |  |  |  |
|  |  |  |  |  |  |  |

##### Mystical Experience Questionnaire (MEQ30)

Looking back on the entirety of your psychedelic session, please rate the degree to which at any time during the session you experienced the following phenomena. Answer each question according to your feelings, thoughts, and experiences at the time of the psychedelic session. In making each of your ratings use the following scale:

0 – none; not at all

1 – so slight cannot decide

2 – slight

3 – moderate

4 – strong (equivalent in degree to any other strong experience)

5 – extreme (more than any other time in my life and stronger than 4)

|  | 0 | 1 | 2 | 3 | 4 | 5 |
| --- | --- | --- | --- | --- | --- | --- |
| Loss of your usual sense of time. |  |  |  |  |  |  |
| Experience of amazement. |  |  |  |  |  |  |
| Sense that the experience cannot be described adequately in words. |  |  |  |  |  |  |
| Gain of insightful knowledge experienced at an intuitive level. |  |  |  |  |  |  |
| Feeling that you experienced eternity or infinity. |  |  |  |  |  |  |
| Experience of oneness or unity with objects and/or persons perceived in your surroundings. |  |  |  |  |  |  |
| Loss of your usual sense of space. |  |  |  |  |  |  |
| Feelings of tenderness and gentleness. |  |  |  |  |  |  |
| Certainty of encounter with ultimate reality (in the sense of being able to “know” and “see” what is really real at some point during your experience. |  |  |  |  |  |  |
| Feeling that you could not do justice to your experience by describing it in words. |  |  |  |  |  |  |
| Loss of usual awareness of where you were. |  |  |  |  |  |  |
| Feelings of peace and tranquility. |  |  |  |  |  |  |
| Sense of being “outside of” time, beyond past and future. |  |  |  |  |  |  |
| Freedom from the limitations of your personal self and feeling a unity or bond with what was felt to be greater than your personal self. |  |  |  |  |  |  |
| Sense of being at a spiritual height. |  |  |  |  |  |  |
| Experience of pure being and pure awareness (beyond the world of sense impressions). |  |  |  |  |  |  |
| Experience of ecstasy. |  |  |  |  |  |  |
| Experience of the insight that “all is One”. |  |  |  |  |  |  |
| Being in a realm with no space boundaries |  |  |  |  |  |  |
| Experience of oneness in relation to an “inner world” within. |  |  |  |  |  |  |
| Sense of reverence. |  |  |  |  |  |  |
| You are convinced now, as you look back on your experience, that in it you encountered ultimate reality (i.e., that you “knew” and “saw” what was really real). |  |  |  |  |  |  |
| Feeling that you experienced something profoundly sacred and holy. |  |  |  |  |  |  |
| Awareness of the life or living presence in all things. |  |  |  |  |  |  |
| Experience of the fusion of your personal self into a larger whole. |  |  |  |  |  |  |
| Sense of awe or awesomeness. |  |  |  |  |  |  |
| Experience of unity with ultimate reality. |  |  |  |  |  |  |
| Feeling that it would be difficult to communicate your own experience to others who have not had similar experiences. |  |  |  |  |  |  |
| Feelings of joy. |  |  |  |  |  |  |

___________________________________________ __________________

Signature Date

##### Brief Savoring Inventory

When I experienced positive events in the past week…

1. I tried to intensify the moment by focusing on it. (1 = strongly disagree, 5 = strongly agree)

1 2 3 4 5

1. I felt grateful for the pleasant event. (1 = strongly disagree, 5 = strongly agree)

1 2 3 4 5

1. I tried to share the positive aspects of that event with another person. (1 = strongly disagree, 5 = strongly agree)

1 2 3 4 5

1. In the last week, I was aware of and appreciated pleasant events. (1 = strongly disagree, 5 = strongly agree)

1 2 3 4 5

1. In the last week, I enjoyed the little things in life more fully. (1 = strongly disagree, 5 = strongly agree)

1 2 3 4 5

1. In the last week, I noticed pleasant things in the face of difficult circumstances. (1 = strongly disagree, 5 = strongly agree)

1 2 3 4 5

___________________________________________ __________________

Signature Date

##### Patient-Reported Experience on Study Therapy

(COMPLETED ON STORYLINE)

Video-response questions: (administered at 2 weeks post MBSR retreat or PAP dosing session)

Please take 3-5 minutes to reflect on how you have been functioning at work, how you have been relating to patients, co-workers, and administrators, and how you have been feeling in your work environment. Note any changes in how you have been feeling or functioning. How have you felt emotionally at work?

1. Please take 3-5 minutes to describe your sense of where you are with mindfulness training. How has the retreat or psilocybin session impacted your practice? How successful have you been with daily practice? How successful have you been in integrating practice into your life even in non-formal meditation settings?
2. If you were in the psilocybin arm of the study please take 3 minutes or so to comment on your impressions of the group format for the psilocybin session. What worked, what didn't work, what would you have liked to see done differently? Please comment on format, music, group process, and anything else you think is relevant.

 Rating questions: : (administered at 2 week mark and at 2 weeks post MBSR retreat or PAP dosing session)

One question that arises in the field of psychedelic medicine is to what extent it is important for therapists to have had prior personal experience with psychedelic medicines themselves.  This is a complicated question and one that raises a number of ethical issues related to potential bias as well as problematics around personal disclosure.  Please answer the following questions as to your perceptions.

(-5 to +5 scale)

1. After your experience how important does it feel to you to have your therapist have had prior personal experience with psilocybin?

-5 -4 -3 -2 -1 0 1 2 3 4 5

1. After your experience how important does it feel to you that the study team has had prior personal experience with psilocybin?

  -5 -4 -3 -2 -1 0 1 2 3 4 5

       3. To what extent does it feel to you that prior personal experience with psilocybin among members of the study team introduces bias in a clinical trial?

  -5 -4 -3 -2 -1 0 1 2 3 4 5

Date

##### PHQ-9

##### QIDS-SR-16

**Quick Inventory of Depressive Symptomatology (Self-Report) (QIDS-SR16)**

1. Falling Asleep:
   1. I never take longer than 30 minutes to fall asleep.
   2. I take at least 30 minutes to fall asleep, less than half the time.
   3. I take at least 30 minutes to fall asleep, more than half the time.
   4. I take more than 60 minutes to fall asleep, more than half the time.
2. Sleep During the Night:
   1. I do not wake up at night.
   2. I have a restless, light sleep with a few brief awakenings each night.
   3. I wake up at least once a night, but I go back to sleep easily.
   4. I awaken more than once a night and stay awake for 20 minutes or more, more than half the time. 3. Waking Up Too Early:
   5. Most of the time, I awaken no more than 30 minutes before I need to get up.
   6. More than half the time, I awaken more than 30 minutes before I need to get up.
   7. I almost always awaken at least one hour or so before I need to, but I go back to sleep eventually.
   8. I awaken at least one hour before I need to, and can’t go back to sleep. 4. Sleeping Too Much:

0 I sleep no longer than 7–8 hours/night, without napping during the day. 1 I sleep no longer than 10 hours in a 24-hour period including naps.

2 I sleep no longer than 12 hours in a 24-hour period including naps. 3 I sleep longer than 12 hours in a 24-hour period including naps.

1. Feeling Sad:
   1. I do not feel sad
   2. I feel sad less than half the time.
   3. I feel sad more than half the time. 3 I feel sad nearly all of the time.
2. Decreased Appetite:
   1. There is no change in my usual appetite.
   2. I eat somewhat less often or lesser amounts of food than usual.
   3. I eat much less than usual and only with personal effort.
   4. I rarely eat within a 24-hour period, and only with extreme personal effort or when others persuade me to eat.
3. Increased Appetite:
   1. There is no change from my usual appetite.
   2. I feel a need to eat more frequently than usual.
   3. I regularly eat more often and/or greater amounts of food than usual. 3 I feel driven to overeat both at mealtime and between meals.
4. Decreased Weight (Within the Last Two Weeks): 0 I have not had a change in my weight.

1 I feel as if I’ve had a slight weight loss. 2 I have lost 2 pounds or more.

3 I have lost 5 pounds or more.

1. Increased Weight (Within the Last Two Weeks): 0 I have not had a change in my weight. 1 I feel as if I’ve had a slight weight gain. 2 I have gained 2 pounds or more.

3 I have gained 5 pounds or more.

10. Concentration/Decision Making:

1. There is no change in my usual capacity to concentrate or make decisions.
2. I occasionally feel indecisive or find that my attention wanders.
3. Most of the time, I struggle to focus my attention or to make decisions. 3 I cannot concentrate well enough to read or cannot make even minor decisions. 11. View of Myself:
4. I see myself as equally worthwhile and deserving as other people.
5. I am more self-blaming than usual.
6. I largely believe that I cause problems for others.
7. I think almost constantly about major and minor defects in myself.
8. Thoughts of Death or Suicide:
   1. I do not think of suicide or death.
   2. I feel that life is empty or wonder if it’s worth living.
   3. I think of suicide or death several times a week for several minutes. 3 I think of suicide or death several times a day in some detail, or I have made specific plans for suicide or have actually tried to take my life.
9. General Interest:
   1. There is no change from usual in how interested I am in other people or activities.
   2. I notice that I am less interested in people or activities.
   3. I find I have interest in only one or two of my formerly pursued activities. 3 I have virtually no interest in formerly pursued activities.
10. Energy Level:
    1. There is no change in my usual level of energy.
    2. I get tired more easily than usual.
    3. I have to make a big effort to start or finish my usual daily activities (for example, shopping, homework, cooking or going to work).
    4. I really cannot carry out most of my usual daily activities because I just don’t have the energy.

1. Feeling Slowed Down:
   1. I think, speak, and move at my usual rate of speed.
   2. I find that my thinking is slowed down or my voice sounds dull or flat 2 It takes me several seconds to respond to most questions and I’m sure my thinking is slowed.

3 I am often unable to respond to questions without extreme effort.

1. Feeling Restless:
   1. I do not feel restless.
   2. I’m often fidgety, wringing my hands, or need to shift how I am sitting. 2 I have impulses to move about and am quite restless.

3 At times, I am unable to stay seated and need to pace around.

##### Challenging Experience Questionnaire

- **0 – none; not at all**
- **1 – so slight cannot decide**
- **2 – slight**
- **3 – moderate**
- **4 – strong**
- **5 – extreme (more than ever before in my life)**
- ______ 1. Isolation and loneliness
- ______ 2. Sadness
- ______ 3. Feeling my heart beating
- ______ 4. I had the feeling something horrible would happen
- ______ 5. Feeling my body shake/tremble
- ______ 6. Feelings of grief
- ______ 7. Experience of fear
- ______ 8. Fear that I might lose my mind or go insane
- ______ 9. I felt like crying
- ______ 10. Feeling of isolation from people and things
- ______ 11. Feelings of despair
- ______ 12. I had the feeling that people were plotting against me
- ______ 13. I was afraid that the state I was in would last forever
- ______ 14. Anxiousness
- ______ 15. I felt shaky inside
- ______ 16. I had the profound experience of my own death
- ______ 17. I felt my heart beating irregularly or skipping beats
- ______ 18. Pressure or weight in my chest or abdomen
- ______ 19. I experienced a decreased sense of sanity
- ______ 20. I felt as if I was dead or dying
- ______ 21. Panic
- ______ 22. Experience of antagonism toward people around me
- ______ 23. Despair
- ______ 24. I felt isolated from everything and everyone
- ______ 25. Emotional and/or physical suffering
- ______ 26. I felt frightened

##### Watts’ Connectedness Scale (WCS)

**Reference**

Watts R, Kettner H, Geerts D, Gandy S, Kartner L, Mertens L, Timmermann C, Nour M, Kaelen M, Carhart-Harris R, Roseman L (2022). The Watts Connectedness Scale: a new scale for measuring a sense of connectedness to self, others, and world. *Psychopharmacology*

**Instructions**

Reflecting on how you have felt over the past 2 weeks, please rate the following items on a scale from **'Not at all' to 'Entirely'** according to how you have felt over this time period. Please answer every item, even if you are unsure or feel the item is unclear or poorly worded. Drag the indicator to a position on the scale that shows how much you agree or disagree with each of the following statements.

Response format

Each item is rated on a 0 – 100 visual analogue scale with the anchors 0 = Not at all, 100 = Entirely

| Final items  *1. I have felt trapped in my mind.* |
| --- |
| *2. My mind has felt connected to my heart/emotion.* |
| *3. I have felt connected to my senses (touch, taste, sight smell, hearing).* |
| *4. I have felt connected to a range of emotions.* |
| *5. If I had chosen to, I could have ‘sat with’ painful memories.* |
| *6. I have felt connected to my body.* |
| *7. I have been able to fully experience emotion, whether positive or negative.* |
| *8. I have felt alone.* |
| *9. I have felt connected to friends and/or family.* |
| *10. I have felt connected to a community.* |
| *11. I have felt connected to all humanity.* |
| *12. I have felt unwelcome amongst others.* |
| *13. I have felt separate from the world around me.* |
| *14. I have felt connected to a purpose in life.* |
| *15. I have felt connected to nature.* |
| *16. I have felt connected to a spiritual essence (in the secular or religious sense).* |
| *17. I have felt connected to a source of universal love.* |
| *18. I have seen things from a broad perspective, ‘the bigger picture’.* |
| *19. I have felt that everything is interconnected.* |

Scoring

Connectedness to Self (CTS): (WCS2 + WCS3 + WCS4 + WCS5 + WCS6 + WCS7) / 6

Connectedness to Others (CTO): ((100 – WCS1) + (100 – WCS8) + WCS9 + WCS10 + (100 – WCS12) + (100 – WCS13)) / 6

Connectedness to World (CTW): (WCS11 + WCS14 + WCS15 + WCS16 + WCS17 + WCS18 + WCS19) / 7

General Connectedness (WCS): (CTS + CTO + CTW) / 3

# PAPR Preference/ Expectancy

Subject ID#:

Subject Initials:

Date: / /

Visit: Screen

# VISUAL ANALOGUE SCALE

Please indicate your **opinion** on the following questions.

1. On a scale of 0 to 100, how **effective** would psilocybin-assisted therapy plus MBSR

be in treating your symptoms? (circle one)

0

10

20

30

40

50

60

70

80

90

Not

Effective

100

Very Effective

2. On a scale of 0 to 100, how **effective** would just MBSR

be in treating your symptoms? (circle one)

0

10

20

30

40

50

60

70

80

90

Not

Effective

100

Very Effective

3. Which do you think will be **more** effective in treating your symptoms? (circle one)

5

Psilocybin

More Effective

4

3

2

1

0

Equally Effective

1

2

3

4 5

MBSR

More Effective

4. Which therapy would you **prefer** for treating your symptoms? (circle one)

MBSR

5

4

3

2

1

0

No Preference

1

2

3

4

5

Prefer psilocybin

Prefer

Staff Initials:

Database entry by: on / /

**PAPR Credibility/Expectancy**

**Subject ID#:**

**Subject Initials:**

**Date: / /**

**Visit: __**

CREDIBILITY / EXPECTANCY QUESTIONNAIRE

We would like you to indicate below how much you believe, **right now**, that the therapy you are going to recieve will help to reduce your anxiety. Belief usually has two aspects to it: (1) what one **thinks** will happen and (2) what one **feels** will happen. Sometimes these are similar; sometimes they are different.

**Set 1**: Answer in terms of what you **think**

1. At this point, how **logical** does the therapy offered to you seem?

1

2

3

4

5

somewhat logical

6

7

8

9

not at all logical

very logical

2. At this point, how successfully do you **think** this treatment will be in reducing your symptoms?

1

2

3

4

5

somewhat useful

6

7

8

not at all useful

9

very useful

3. How **confident** would you be in recommending this treatment to a friend who experiences similar problems?

1

2

3

4

5

somewhat confident

6

7

8

9

not at all confident

very confident

4. By the end of the therapy period, how much improvement in your symptoms do you **think** will occur?

0% 10% 20% 30% 40% 50% 60% 70% 80% 90% 100%

**Set 2**: Answer in terms of what you really and truly **feel** about the therapy and its likely success.

1. At this point, how much do you really **feel** that therapy will help you to reduce your symptoms?

1

not at all

2

3

4

5

somewhat

6

7

8 9

very much

2. By the end of the therapy period, how much improvement in your symptoms do you really **feel** will occur?

0% 10% 20% 30% 40% 50% 60% 70% 80% 90% 100%

Staff Initials:

Database entry by: on / /

v.Jan2011 Page 1 of 1

1. Follow up measures to be administered 14 days (+/- 3) and 180 days (+/-7) after final MBSR session in week 8. [↑](#endnote-ref-1)
2. Vital signs include systolic and diastolic blood pressure, heart rate, respiratory rate, pulse oximetry, weight. Weight captured at screening only. [↑](#endnote-ref-2)
3. On day of psilocybin administration vital signs will be assessed approx. 10 min before dosing and at 30, 50, 90, 120, 180, 240, 300, and 360 minutes after psilocybin administration. [↑](#endnote-ref-3)
4. All Questionnaires scheduled for MBSR Week 1 to be administered prior to the first MBSR session. [↑](#endnote-ref-4)
5. Integration sessions #1 and #2 both included in this week for the PAP + MBSR arm. Adverse event collection, suicidality, concomitant meds will all be assessed x 2 during this week for the PAP+MBSR arm. [↑](#endnote-ref-5)
6. Adverse event collection will occur during all 3 preparatory sessions and all 3 integration sessions for the PAP+MBSR arm. [↑](#endnote-ref-6)
