# Supplementary material for: Psilocybin-assisted group psychotherapy and mindfulness-based stress reduction for frontline healthcare provider COVID-19-related depression and burnout: A randomized controlled trial
Source: PLoS Med. 2025 Sep 19;22(9):e1004519. doi: 10.1371/journal.pmed.1004519 (PMC12459851; doi:10.1371/journal.pmed.1004519)
Supplement: S1 — Extension checklist for reporting trials modified due to extenuating circumstances. (DOCX) [file pmed.1004519.s004.docx]

# CONSERVE-CONSORT Checklist

## I. Extenuating Circumstances

The trial was conducted during the COVID-19 pandemic, which represented a major extenuating circumstance affecting study operations, recruitment, and participant burden. Additionally, a minor protocol modification occurred: increasing the planned enrollment from 24 to 25 participants.

## II. Important Modifications

1. Enrollment increased from 24 to 25 participants. This was to allow for a total of 12 participants to enroll in the MBSR+PAP arm. This had no substantive impact on design, power, or interpretation.

2. Trial operations were adapted to the COVID-19 pandemic context. Extenuating circumstances included scheduling difficulties due to healthcare system strain, social distancing and masking requirements, and the need for additional infection control measures. These did not alter the core design, interventions, or outcomes, but impacted logistics, recruitment pace, and participant flow.

## III. Responsible Parties

The modifications were reviewed and approved by the PI and study team, with input from the trial statistician. The IRB approved the final enrollment count. COVID-19 related adaptations were managed institutionally in accordance with institutional policy and public health and safety protocols.

## IV. Interim Data

No modifications were based on interim trial outcome data. The decision to increase enrollment to 25 was administrative. COVID-19 operational adjustments were external and not informed by trial data.

## CONSORT Items

| CONSORT Item | No Change | Impact | Mitigating Strategy |
| --- | --- | --- | --- |
| 1. Title/Abstract | X |  |  |
| 2. Introduction | X |  |  |
| 3. Methods: Trial Design | X |  |  |
| 4. Methods: Participants |  | COVID-19 context required masking, distancing, and altered logistics | Ensured safety protocols and scheduling flexibility |
| 5. Methods: Interventions | X |  |  |
| 6. Methods: Outcomes | X |  |  |
| 7. Methods: Sample Size |  | Increased from 24 to 25 participants | Allowed final eligible participant to be included |
| 8-10. Methods: Randomisation | X |  |  |
| 11. Methods: Blinding | X |  |  |
| 12. Methods: Statistical Methods | X |  |  |
| 13. Results: Participant Flow |  | Recruitment during pandemic; final N=25 | Documented adaptations and extended recruitment timeline |
| 14. Results: Recruitment |  | COVID-19 slowed recruitment pace | Flexible scheduling, remote screening where feasible |
| 15. Results: Baseline Data | X |  |  |
| 16. Results: Numbers Analysed |  | Analyses reflect N=25 | Analyses conducted with all randomized participants (mITT) |
| 17. Results: Outcomes and Estimation | X |  |  |
| 18. Results: Ancillary Analyses | X |  |  |
| 19. Results: Harms | X |  |  |
| 20. Discussion: Limitations |  | COVID-19 context and small N may limit generalizability | Explicitly noted in discussion |
| 21. Discussion: Generalisability |  | Limited by pandemic-specific context | Reported transparently |
| 23. Registration | X |  |  |
| 24. Protocol | X |  |  |
| 25. Funding | X |  |  |
