# Supplementary material for: Psilocybin-assisted group psychotherapy and mindfulness-based stress reduction for frontline healthcare provider COVID-19-related depression and burnout: A randomized controlled trial
Source: PLoS Med. 2025 Sep 19;22(9):e1004519. doi: 10.1371/journal.pmed.1004519 (PMC12459851; doi:10.1371/journal.pmed.1004519)
Supplement: Attachment — Submitted filename: Review of PMEDICINE.docx [file pmed.1004519.s006.docx]

**Review of PMEDICINE-D-24-04423R1**

This manuscript presents results from a clinical trial comparing *MBSR+PAP to MBSR-only in terms of symptoms of depression and burnout among frontline clinical workers during the COVID-19 pandemic. My comments are:*

- *The analysis of depressive symptoms suggests a significant difference at 2 weeks but not at 6 months. This needs to be better reflected in the manuscript (discussion, results, and abstract). For example, “Meanwhile, MBSR+PAP was associated with clinically and statistically significant decreases in depressive symptoms” should be augmented with at 2 weeks but not at 6 months. I think some discussion of why the effect is not sustained (at least at the same magnitude) is also warranted.*
- *Did the linear mixed model involve the baseline scores (e.g., by including them in the model or by modeling change from baseline)? Given the small sample size and what seem like non-trivial difference at baseline in the figures, accounting for baseline scores would strengthen the results.*
- *The protocol suggests that there are two primary endpoints (at 2 weeks and six months) and the analysis should be multiplicity adjusted.*
- *The COVID pandemic created unique situation on frontline workers. How does that affect the generalizability of the results?*
- *“Patients were recruited from December….” Are these patients (i.e., do they have a disese)?*
- *The list of secondary outcomes in the protocol has a lot of analysis not outcomes and these should be listed under secondary analysis not outcomes.*
- *Please specify what statistical test was used in the power analysis and whether a one sided or a two sided test was used.*
- *What distributional assumptions were made on the random intercept in the mixed effect model.*
- *Did the mixed effect model have any convergence issues (often a problem with small sample sizes)?*
- *There are pretty large gender differences between arms (50% vs 92%). This suggest that gender should be accounted for in the analysis.*
- *Was there any missing data? If yes, how was that adjusted for?*
- *Was there any dropout? If yes, please specify how much.*
- *Is there possibility that the group sessions create some within group correlations? If yes, these should be adjusted for in the analysis.*
