# Supplementary material for: Psilocybin-assisted group psychotherapy and mindfulness-based stress reduction for frontline healthcare provider COVID-19-related depression and burnout: A randomized controlled trial
Source: PLoS Med. 2025 Sep 19;22(9):e1004519. doi: 10.1371/journal.pmed.1004519 (PMC12459851; doi:10.1371/journal.pmed.1004519)
Supplement: Attachment — Submitted filename: Response to Reviewers, final.docx [file pmed.1004519.s008.docx]

**Review of PMEDICINE-D-24-04423R1: Response to Reviewer Comments**

We thank the reviewers of this manuscript and the PLOS Medicine editorial staff for this opportunity to substantially improve our paper and presentation of study results. I have responded to each reviewer comment here with notation as to relevant changes in the manuscript, data presentation, and Supplementary Appendix. I have included text that has been added or changed and referenced the line numbers (in accordance with the attached clean copy of the manuscript).

**Reviewer 1:**

This manuscript presents results from a clinical trial comparing *MBSR+PAP to MBSR-only in terms of symptoms of depression and burnout among frontline clinical workers during the COVID-19 pandemic. My comments are:*

1. *The analysis of depressive symptoms suggests a significant difference at 2 weeks but not at 6 months. This needs to be better reflected in the manuscript (discussion, results, and abstract). For example, “Meanwhile, MBSR+PAP was associated with clinically and statistically significant decreases in depressive symptoms” should be augmented with at 2 weeks but not at 6 months. I think some discussion of why the effect is not sustained (at least at the same magnitude) is also warranted.*

Thanks for this important suggestion. We have revised the manuscript to better characterize this effect at 2 weeks but not 6 months. We have added language to the Results section (under ‘preliminary efficacy’ and Discussion section in this regard as well as made changes to the Abstract.

**see Abstract lines 120-122**: “The MBSR+PAP arm evidenced a significantly larger decrease in QIDS-SR-16 score than the MBSR-only arm from baseline to 2-weeks post-intervention (an effect that waned at the 6-month follow-up)”

**Results lines 391-394**: “There were no significant between-group differences in QIDS-SR-16 scores at the 6-month endpoint, with participants in both arms showing significant decreases in depression symptoms from baseline. However the mixed model analysis across all 3 time points showed a significant difference favoring MBSR+PAP (p=0.02).”

**Discussion lines 493-497**: “Notably, the antidepressant effects of PAP were strongest at the 2-week endpoint. By the 6-month endpoint depression scores for participants in MBSR-only approached those of participants in MBSR+PAP, suggesting that psilocybin may accelerate the therapeutic benefits of mindfulness.[44]

2. *Did the linear mixed model involve the baseline scores (e.g., by including them in the model or by modeling change from baseline)? Given the small sample size and what seem like non-trivial difference at baseline in the figures, accounting for baseline scores would strengthen the results.*

The reviewer makes an excellent point. While it is common to adjust for baseline scores as covariates in mixed models, for our *a priori* pre-specified analysis we originally chose to compute linear mixed models including the baseline measurement point as a repeated measure in the analysis. This approach produces the equivalent of change scores which allowed us to directly assess symptom improvement and present results in a clinically intuitive way to readers. However, we agree that given the small sample size, accounting for baseline scores could be helpful. To address concerns here we have performed an additional sensitivity analysis with a mixed effects repeated measures ANCOVA model using baseline scores as a covariate, with the outcome at 2 weeks and 6 months as repeated measures. The primary fixed effect of interest in these models is the baseline adjusted treatment main effect, which estimates the overall benefit of MBSR+PAP vs. MBSR averaged across both follow-ups. Models included random intercepts for participant and for group cohort, to account for clustering effects. We have reported those results in the Supplementary Appendix (eTables 12-16). In this analysis the main effect of study arm was significant for QIDS-SR-16, MBI(EE), MBI(DP), and WCS(GC) favoring the MBSR+PAP group. (Given only 2 timepoints in DSII this analysis was not performed). We have included these baseline-adjusted mixed model results in the Supplementary Appendix however our primary conclusions remain unchanged.

I have described this analysis in the Results section lines 396-400: “To account for possible effects of baseline differences across outcome measures we conducted mixed RM-ANCOVA analyses adjusting for baseline differences, which showed a significant main effect of treatment across the 2-week and 6-month endpoints favoring MBSR+PAP. These are reported in **S12-S16 Tables**.”

(also see Reviewer 3, #3).

3. *The protocol suggests that there are two primary endpoints (at 2 weeks and six months) and the analysis should be multiplicity adjusted.*

Based on this reviewer suggestion, we have adjusted for multiple comparisons in several ways. This is expanded on in response to Reviewer #2 below. To adjust for 2 primary endpoints (2 weeks and 6 months) with our primary outcome measure (QIDS-SR-16) we used a Bonferroni correction with resultant p-value of 0.008 at the 2-week endpoint and 0.76 for the 6-month endpoint. (corrected for 2 measures). See below responses to Reviewer #2 for corrections for multiple comparisons across secondary outcomes. In addition, the new sensitivity analysis (mixed effects repeated measures ANCOVA) analyzes the effect of study arm across both endpoints (2 weeks and 6 months) simultaneously and thus no multiplicity adjustment is needed for these analyses. This is now described in the Statistical Analysis section and Results.

4. *The COVID pandemic created unique situation on frontline workers. How does that affect the generalizability of the results?*

The reviewer makes a good point. We have added this sentence to the discussion (lines 548-551): “The COVID-19 pandemic created unique challenges for frontline workers and it is possible that symptoms of depression and burnout engendered in this specific milieu may not generalize more widely across healthcare environments. However literature supports the amplifying effect of the pandemic on pre-existing vulnerabilities.”

5. *“Patients were recruited from December….” Are these patients (i.e., do they have a disease)?*

Thanks for catching this, we have corrected to ‘participants’. While some study participants were pre-existing patients receiving mental health treatment some participants had not yet sought care.

6. *The list of secondary outcomes in the protocol has a lot of analysis not outcomes and these should be listed under secondary analysis not outcomes.*

We have changed the language in the protocol to reflect ‘analysis’ instead of ‘outcomes’.

7. *Please specify what statistical test was used in the power analysis and whether a one sided or a two sided test was used.*

Thank you for this opportunity to better describe our power analysis.

### We have added this language in the ‘Statistical Analysis’ section to expanded on the sample size calculations as follows (lines 307-315): “A meta-analysis examining the effects of psilocybin on depressive symptoms reported an effect size of Cohen’s d=1.29. (40)  Previous meta-analyses of MBSR indicate modest to moderate effect sizes for depressive symptom reduction (Cohen’s d= 0.3 to 0.4). (41) We conducted simulation-based power calculations for the linear mixed-effects model. Assuming a **large between group effect size (Cohen’s d = 1.0)**, alpha=0.05, and a **moderate-to-high intra-class correlation (ICC = 0.5)** reflecting the repeated measures design, the estimated power to detect a **treatment group × time interaction** in the LMM was approximately **81%**. Given the pilot nature of this study, the primary goal was to estimate effect sizes and assess feasibility to inform future larger-scale trials.

8. *What distributional assumptions were made on the random intercept in the mixed effect model.*

We assumed a normal distribution with mean of 0.

9. *Did the mixed effect model have any convergence issues (often a problem with small sample sizes)?*

No, there were no convergence issues with our mixed models.

10. *There are pretty large gender differences between arms (50% vs 92%). This suggests that gender should be accounted for in the analysis.*

We accounted for gender as a covariate in the mixed model and the results remained unchanged for all outcomes. We have added a sentence in the Results section under preliminary efficacy to this effect for clarification with the following language (lines 394-396): “We conducted sensitivity analyses adjusting for baseline differences in depression, gender, and clustering within group cohorts and significant effects were unchanged.”

11. *Was there any missing data? If yes, how was that adjusted for?*

We did have baseline data for participants who dropped out of the study and these participants were included in ITT analysis. Mixed model analysis with maximum likelihood estimation of missing data was used to handle missing data (missing data were MCAR according to Little’s test) so that we did not need to use any imputation for missing data.

12. *Was there any dropout? If yes, please specify how much.*

Lines 346-348 in Results specify 1 drop out in PAP+MBSR and 2 drop outs in MBSR only. This is also reflected in the CONSORT diagram.

13. *Is there possibility that the group sessions create some within group correlations? If yes, these should be adjusted for in the analysis.*

The reviewer makes a good point – group sessions could create some clustering effects. We ran an additional analysis including group cohort clustering as a random effect in the mixed model analysis. Also, the sensitivity analysis via the mixed effects repeated measures ANCOVA included group cohort as a random effect. The intraclass correlations (ICCs) for group cohort were ≤.02. Adding group cohort to the models did not change the significance of any observed effects. We have added the sensitivity analysis to the Results section, as described above: “We conducted sensitivity analyses adjusting for baseline differences in depression, gender, and clustering within group cohorts and significant effects were unchanged.”

.

**Reviewer #2:**

*This manuscript reports findings from a randomized controlled trial comparing MBSR alone versus MBSR plus psilocybin-assisted psychotherapy (PAP) for frontline healthcare providers with depression and burnout related to COVID-19. The study found that MBSR+PAP produced greater improvements in depression symptoms, burnout measures, reduced demoralization, and increased connectedness at the 2-week endpoint compared to MBSR alone, with no serious adverse events reported.
The work is timely and present a novel combination of mindfulness training with psilocybin in a group format, and is compelling in regards to the design in that the study uses an active control condition (MBSR) rather than waitlist or placebo only.
This work is a valuable contribution that addresses an important clinical need with a novel therapeutic approach.*

Thank you for recognizing the value of this manuscript.

*1.      Clarify the primary justification for the imbalance in therapeutic contact time between arms.*

We agree this is an important point to be clarified. We have added the following language under ‘Interventions’ (lines 259-261): “The difference in therapeutic contact time between study arms was based on the priority for ensuring safety of psilocybin administration while minimizing total intervention time for busy healthcare professionals.” We have also addressed this imbalance in the discussion (see below).

*2.      Address how this contact time difference could influence interpretation of results*

(see below)

*3.      Consider reporting a sensitivity analysis adjusting for total therapeutic contact hours.*

There was a significant difference between study arms in terms of total therapeutic contact time (approx. 28 hrs for MBSR only and 40 hours for MBSR+PAP accounting for the addition of preparatory and integration sessions. Given that study arm assignment is already a proxy for this difference in therapeutic contact hours (that is, a variable representing total contact hours is extremely highly colinear with the study arm assignment variable), we re-ran the mixed model analysis using MBSR attendance as a covariate. (MBSR attendance was variable across study arms and was the primary source of individual difference in therapeutic time for participants in each arm, particularly given essentially 100% attendance at the preparatory and integration sessions). There was no significant effect for MBSR attendance as a covariate, nor did adding this covariate to the models change the study results. We have reported this in the Results section.

*4.      Include a correction for multiple comparisons given the numerous outcome measures*

In response to this reviewer comment, we have addressed this suggestion in two ways. As per response to Reviewer 1, #3 we corrected for multiple comparisons for our primary outcome measure (QIDS-SR-16) across 2 primary endpoints with a Bonferroni correction with corrected p-values of 0.008 at the 2-week endpoint and 0.76 for the 6-month endpoint.

The study was powered to detect effects on the **primary outcome only** (QIDS-SR-16) with an alpha of 0.05, and the secondary outcomes were intended to **generate hypotheses and guide future investigation**, not to support confirmatory conclusions. In this regard, to account for the increased risk of Type I error due to multiple comparisons across secondary outcomes, we applied a False Discovery Rate (FDR) correction using the Benjamini-Hochberg procedure.

Using a FDR correction for 9 tests across the secondary outcomes (accounting for 2-week and 6-month outcome measurements) and rank ordering secondary outcome effect sizes we retain significance only for the WCS(GC) at 2 weeks, (adjusted p = (p × 9 / i)). Raw and FDR-adjusted p-values are reported below and in the Supplementary Appendix (eTable 11). I have added these findings to the Results and Discussion sections.

| Outcome | Raw p-value | FDR-adjusted p-value |
| --- | --- | --- |
| WCS(GC) at 2 weeks | **0.005** | **0.045** |
| MBI(EE) at 6 months | **0.016** | 0.072 |
| DSII at 2 weeks | **0.029** | 0.087 |
| MBI(DP) at 2 weeks | **0.038** | 0.086 |
| MBI(DP) at 6 months | 0.067 | 0.1206 |
| MBI(EE) at 2 weeks | 0.078 | 0.1206 |
| WCS(GC) at 6 months | 0.090 | 0.1206 |
| MBI(PA) at 2 weeks | 0.150 | 0.1688 |
| MBI(PA) at 6 months | 0.260 | 0.260 |

We have added the following language to the Statistical Analysis section (lines 324-327): “A Bonferroni correction was used to correct for multiple comparisons across the primary outcome measure and False Discovery Rate (FDR) correction using the Benjamini-Hochberg procedure was used to correct for multiple comparisons across secondary outcomes.”

In addition, the new sensitivity analysis (mixed effects repeated measures ANCOVA) analyzes the effect of study arm across both endpoints (2 weeks and 6 months) simultaneously.

*5.      Include absolute scores at each time point in tables, not just change scores*

We have changed table 3 to include baseline, 2-week, and 6-week scores in addition to change scores. I have also adjusted this table to present both adjusted p-values as well as effect sizes (Cohen’s d).

*6.      Provide more detailed information about clinical significance (what percentage of participants achieved remission?)*

We have added the following language to clarify this in Results (lines 387-391): “A QIDS-SR-16 score of less than or equal to 5 is considered remission. At the 2-week endpoint 6 participants (46%) in the MBSR+PAP arm achieved remission vs. 1 participant (8.3%) in MBSR only. At the 6-month endpoint 7 participants in the MBSR+PAP arm achieved remission (53.8%) vs. 2 in MBSR only (16.7%).”

*7.      Include a supplementary table showing correlations between all outcome measures 10x10 matrix for 2 week data.*

We have created **eTable 10** in the Supplementary Appendix which shows correlations (and p-values) between all outcome measures at the 2-week endpoint. We have indicated this in the Results section as well.

*8.      Expand the limitations section to fully acknowledge the potential confounding effect of therapeutic contact time*

We have added the following language to the Discussion section (lines 549-553): “PAP+MBSR arm received a larger amount of total therapeutic contact time given the additional preparatory and integration sessions. This difference in therapeutic contact time may have had a confounding effect on outcomes. Future studies should equate therapeutic contact time between study arms.” Also see response to #1 above.

*9.      Provide a more detailed analysis of how expectancy effects might have influenced outcomes*

We have added some additional language to the Discussion section on expectancy effects (lines 523-525): “It is possible however that associated disappointment in not being randomized to the psilocybin condition (may have had an effect on outcomes in the MBSR-only arm, as well as higher drop-out rate in that arm.”

*10.     Elaborate on the potential mechanisms of action, particularly regarding connectedness measures*

Thanks for this opportunity to elaborate on potential mechanisms. This is clearly preliminary based on this study’s outcomes and available evidence to date. I have added this clause to the Discussion section (lines 506-511): “Psilocybin has been recognized for its capacity to enhance feelings of social connectedness, empathy, and interpersonal openness. It is possible that the combination of neurobiological effects, subjective experiences of unity and empathy occasioned by psilocybin, and interpersonal processes within the group format may contribute to prosocial emotional processing and reduced self-isolation which are mechanisms underlying improvement in social connectedness and well-being.” I included here a citation for the paper Carhart-Harris et al (2018) Psychedelics and Connectedness. *Psychopharmacology* DOI: [10.1007/s00213-017-4701-y](https://doi.org/10.1007/s00213-017-4701-y)

*Minor revisions:* 
*a. Include more details about MBSR trainer qualifications and fidelity monitoring*

We have added the following language (Methods lines 234-240): “Participants enrolled were enrolled in a standard MBSR course led by a certified instructor trained in MBSR through the Brown University Mindfulness Center which involved eight weekly, two-hour group sessions in which mindfulness meditation training (e.g., mindful breathing, body scan) and psychoeducation.(29) The MBSR certification pathway involves formal instruction, personal practice, teaching practice, and structured supervision. This includes video and audio review of sessions with structured feedback and evaluation with standardized fidelity measures including the MBSR Teacher Competency Assessment (MBI-TAC).”

*b. Specify how blinding was maintained for outcome assessments*

We have expanded the description under Masking and Randomization (lines 228-230): “Assessments were conducted as self-report measures by participants. To maintain blinding, the study key with allocations was inaccessible to the statistician and outcome assessors.”

*c. Clarify the procedure for tapering antidepressants in the MBSR+PAP arm.*

We have described under ‘Interventions’ the following (lines 258-259): “Medication taper was overseen by the study team in conjunction with the participant’s outpatient provider.” I have also added under ‘Results’ lines 254-256): “While a majority of study participants had a history of antidepressant treatment only 2 participants in the PAP+MBSR arm required supervised taper of existing antidepressant medication.”

We have also added language to the Discussion section elaborating on this (lines 557-562): “The study protocol required that participants randomized to MBSR+PAP taper existing antidepressant medication whereas those randomized to MBSR only were allowed to continue existing treatment. This was done to minimize potential harm of antidepressant discontinuation on participants assigned to MBSR only however may have influenced outcomes with possible worsening of symptoms for participants required to taper with possible blunting of effects seen in the MBSR+PAP arm.”

*4.      Report adherence rates for MBSR sessions by study arm separately .*

We have added under ‘Safety and Feasibility’ (lines 374-377): “Attendance of scheduled MBSR sessions was 80.9% across both arms with no significant difference between arms (85.4% +/- 6.12 in MBSR+PAP vs 79.2% +/- 6.09 in MBSR only).”

*5.      Include more detailed information about missing data and the approach to handling it*

As per Reviewer 1, #11: We did have baseline data for participants who dropped out of the study and these participants were included in ITT analysis. Mixed model analysis with maximum likelihood estimation of missing data was used to handle missing data (missing data were MCAR according to Little’s test) so that we did not need to use any imputation for missing data. Little’s MCAR test: Chi-Squared=0.009, DF=1, Sig=0.0925. See **S17 Table** in Supplement.

*6.      Provide data on potential predictors of response (e.g., prior psychedelic use)*

We have included the following in ‘Results’ (lines 441-442): “t-tests on change scores in QIDS-SR-16 at the 2-week endpoint did not show any effect of prior psychedelic experience on outcomes.” We have also included gender as a covariate in the mixed model analysis with no significant effect on outcomes.

**Reviewer #3:**

*The investigators are to be congratulated on doing an important small-scale study with a highly novel design that is both clinically relevant and scientifically interesting. The comparison of MBSR vs. MBSR + PAP is really novel.*

Thank you for recognizing the importance of this study.

*1. I suggest providing more information on the day-long MBSR retreat in the manuscript itself. There is a lot of info in the supplement but many readers won't get there and better understanding what was done that day is important because it is the stand-in for the PAP--really the only comparator element. Also I didn't see a mention in the limitations section regarding the fact that it appears the PAP group got additional interventional time compared to the MBSR group. Not a fatal flaw but a potential confound.*

Thank you for this suggestion. We have added language about the MBSR retreat day to the Methods section as well as language to the Discussion section addressing potential confounding effects of differential therapeutic contact time: (Methods line 253-256) “This silent meditation retreat occurred after week 6 of the MBSR curriculum and was held in-person over an 8 hour day where participants engaged in silent meditation practice, body scan techniques, gentle yoga practice, and a brief group check-in at the end of the day, all led by the certified MBSR instructor.”

Discussion (lines 550-554): “PAP+MBSR arm received a larger amount of total therapeutic contact time given the additional preparatory and integration sessions. This difference in therapeutic contact time may have a confounding effect on outcomes. Future studies should equate therapeutic contact time between study arms.”

*2. The sample size description in the manuscript is a bit confusing. A large effect size was anticipated, but I didn't see any mention of the expected between group difference effect size vs. MBSR. With 25 total sample one either expects huge psilocybin effects or very modest MBSR effects for their to be a group difference. A bit of elaboration of what the projected effect size represents (between group difference, difference from baseline etc) would be helpful.*

Thank you for this opportunity to better describe our power analysis.

### We have added this language in the ‘Statistical Analysis’ section (lines 307-315) to expanded on the sample size calculations as follows: “A meta-analysis examining the effects of psilocybin on depressive symptoms reported an effect size of Cohen’s d=1.29. (40)  Previous meta-analyses of MBSR indicate modest to moderate effect sizes for depressive symptom reduction (Cohen’s d= 0.3 to 0.4). (41) We conducted simulation-based power calculations for the linear mixed-effects model. Assuming a **large between group effect size (Cohen’s d = 1.0)**, alpha=0.05, and a **moderate-to-high intra-class correlation (ICC = 0.5)** reflecting the repeated measures design, the estimated power to detect a **treatment group × time interaction** in the LMM was approximately **81%**. Given the pilot nature of this study, the primary goal was to estimate effect sizes and assess feasibility to inform future larger-scale trials.

*3. It is not clear whether baseline values of the outcome measures were included in the LMMs? If they were this should be mentioned. If not, adding them would be standard and might further sharpen outcome results.*

The reviewer makes an excellent point. While it is common to adjust for baseline scores as covariates in mixed models, for our *a priori* pre-specified analysis we originally chose to compute linear mixed models including the baseline measurement point as a repeated measure in the analysis. This approach produces the equivalent of change scores which allowed us to directly assess symptom improvement and present results in a clinically intuitive way to readers. However, we agree that given the small sample size, accounting for baseline scores could be helpful. To address concerns here we have performed an additional sensitivity analysis with a mixed effects repeated measures ANCOVA model using baseline scores as a covariate, with the outcome at 2 weeks and 6 months as repeated measures. The primary fixed effect of interest in these models is the baseline adjusted treatment main effect, which estimates the overall benefit of MBSR+PAP vs. MBSR averaged across both follow-ups. Models included random intercepts for participant and for group cohort, to account for clustering effects. We have reported those results in the Supplementary Appendix (eTables 12-16). In this analysis the main effect of study arm was significant for QIDS-SR-16, MBI(EE), MBI(DP), and WCS(GC) favoring the MBSR+PAP group. (Given only 2 timepoints in DSII this analysis was not performed). We have included these baseline-adjusted mixed model results in the Supplementary Appendix however our primary conclusions remain unchanged.

I have described this analysis in the Results section lines 396-400: “To account for possible effects of baseline differences across outcome measures we conducted mixed RM-ANCOVA analyses adjusting for baseline differences, which showed a significant main effect of treatment across the 2-week and 6-month endpoints favoring MBSR+PAP. These are reported in **S12-S16 Tables**.”

(Also see Reviewer 1, #2).

*4. Correlations were used to examine associations of acute psychedelic effects with outcomes and if I'm reading the results and discussion section rightly no independent effect of any of the experiential scales (e.g., MEQ) independently predicted outcome. But it might still be worth using a regression model that adjusts for baseline score and treatment arm to confirm that these outcomes did not differentially predict behavioral response to PAP vs. MBSR. It was also not clear from either the methods or results section that correlations between MEQ etc. and outcomes was being done on the population as a whole. it was only in the discussion that this seems to have been the case. Being clearer on what was done prior to that would strengthen the paper.*

The reviewer makes a good point. We have addressed this point in the following ways: we added a table that presents correlations between experiential scales and outcomes by study arm (eTable 9 in Supplementary Appendix) to present this in addition to the population as a whole. We also clarified the language in the manuscript in the Results section to better characterize and expand on these correlations. Based on this reviewer’s suggestion we additionally ran a regression analysis looking at the interaction term of treatment arm x MEQ (after controlling for the baseline value of the outcome) and found no significant interaction effect.

*5. I'd recommend reordering the results section so that all week 2 findings are presented first and then all 6 month findings are presented next--that makes it seem less like cherry picking what is being presented in the manuscript. On that note, in the written results section I'd suggest adding the fact that there was no between group difference in the QIDS at 6 months. On that note, the figures really suggest that PAP produced a more rapid benefit but that this benefit peaked at 2 weeks and did not mature into even further improvement with time, whereas MBSR didn't do much initially but seemed to catch up over time. And for QIDS it seems that the psychedelic benefit actually waned over time. This sort of pattern was seen in all the scales and it might mean something important—*

Thank you for this suggestion and observations. It is true that the benefit of psilocybin waned over time. We have reorganized the Results section along these lines for clarity and have better articulated the absence of between group differences in the QIDS-SR-16 at the 6-month endpoint in the Results (lines 391-394) and Discussion (lines 493-497) sections.
