# Supplementary material for: Psilocybin-assisted group psychotherapy and mindfulness-based stress reduction for frontline healthcare provider COVID-19-related depression and burnout: A randomized controlled trial
Source: PLoS Med. 2025 Sep 19;22(9):e1004519. doi: 10.1371/journal.pmed.1004519 (PMC12459851; doi:10.1371/journal.pmed.1004519)
Supplement: Attachment — Submitted filename: Final Response to Reviewers and Editor 8_26.docx [file pmed.1004519.s009.docx]

Dear Reviewers and PLOS Medicine Editorial Staff,

Thank you for your thoughtful engagement with our manuscript and helpful suggestions. I have responded to these below, with references to changes in the manuscript text where applicable.


------------------------------------------------------------
Requests from Editors:

EDITORIAL REQUESTS
* At this stage, we ask that you include a short, non-technical Author Summary of your research to make findings accessible to a wide audience that includes both scientists and non-scientists. The Author Summary should immediately follow the Abstract in your revised manuscript. This text is subject to editorial change and should be distinct from the scientific abstract. Ideally each sub-heading should contain 2-3 single sentence, concise bullet points containing the most salient points from your study. In the final bullet point of ‘What Do These Findings Mean?’ Please include the main limitations of the study in non-technical language.

**We have included an Author Summary below the abstract in the manuscript.**

* Please confirm that your title complies with to PLOS Medicine's style. Your title must be nondeclarative and not a question. It should begin with main concept if possible. "Effect of" should be used only if causality can be inferred, i.e., for an RCT. Please place the study design ("A randomized controlled trial," "A retrospective study," "A modelling study," etc.) in the subtitle (ie, after a colon). Please remove the ‘+’ sign from the title.

**I have edited the title to: Psilocybin-Assisted Group Psychotherapy and Mindfulness Based Stress Reduction for Frontline Healthcare Provider COVID-19 Related Depression and Burnout: A Randomized Clinical Trial**

* Please confirm that your abstract complies with our requirements, including format (three sections: Background, Methods and Findings, and Conclusions) and providing all the information relevant to this study type <https://journals.plos.org/plosmedicine/s/submission-guidelines#loc-abstract>

**Have revised the abstract along these guidelines.**

* Please remove the “Objective” subtitle in the Abstract and start with Background, before you explain what is the objective of this trial.

**Completed.**

* Please also note that all abbreviations must be spelled out at first use and abbreviations should not be used in the title.

**Have ensured all abbreviations are spelled out at first use and abbreviations are not used in the title.**

* Please use the active voice in the manuscript and throughout the text.

**We have made revisions to utilize the active voice rather than passive voice through the manuscript.**

* The Abstract must be rewritten to conform to CONSORT 2025 guidelines. The primary objective and endpoint must be stated in the Abstract and please indicate if met. Please also state if descriptive statistics were used.

**Have revised the abstract accordingly.**

* Please clarify in the Abstract when the study was performed.

**Have included dates of enrollment.**

* If all secondary outcomes described in the protocol are included in the manuscript, please briefly describe them in the Abstract. If only a subset is reported in this study, please clarify why in the Methods and do not report in the Abstract.

**Have removed secondary outcomes from the abstract so as to elaborate in the Methods. See lines 327-329.**

* If safety is an outcome, please so state.

**Have included the following in the abstract: “Safety was a study outcome and assessed by rate and severity of AEs and any incident suicidality or significant mental health symptoms.”**

* Please ensure that the Introduction ends with a clear description of the study question or hypothesis.

**Have included this sentence in the Background section: “We hypothesized that psilocybin would augment and accelerate antidepressant effects of MBSR in this population.”**

* The Methods and Findings subtitle should include the results.

**Have included results for QIDS scores. As per above we removed discussion of secondary outcomes here to elaborate in the Methods section.**

* Please ensure that you have included statistics for any difference indicates as statistically significant (with confidence intervals). Differences observed that were not statistically significant must be qualified as such.

**Have ensured these are included.**

* Please confirm that all numbers presented in the abstract are present and identical to numbers presented in the main manuscript text.

**Confirmed.**

* In the last sentence of the Abstract Methods and Findings section, please describe the main limitation(s) of the study's methodology.

**Have included this sentence: “Study limitations that affect the generalizability of results include a small sample size, homogenous study population, and significant differences in intervention intensity.”**

* In the abstract, please include the important dependent variables that are adjusted for in the analyses.

**Included the following: “A mixed RM-ANCOVA was conducted to control for baseline differences in outcome measures. Sensitivity analyses were conducted adjusting for baseline differences in gender and clustering within group cohorts.” We have also included tables S1b, S2b, S3b, S4b in the Supplementary Appendix which show mixed model analyses adjusting for gender and group cohort (as fixed effects).**

* Dates of first and last participant enrolment must be included in the manuscript.

**We described this in the manuscript in the Methods section in relation to recruitment and in the Results section (December 2022 and February 2024, respectively)**

* Consent is not described as ‘informed’. Please confirm that you obtained informed consent.

**Yes, confirmed and have included ‘informed’ in the description.**

* Please ensure that where relevant figures include 95% CIs.

**We have recreated the figures using 95% CIs instead of standard errors. (Have also adjusted these to discrete time points as opposed to continuous lines as suggested in a later comment).**

* If a Data Safety Monitoring Board was involved in the study, please identify it in the Methods. If it was not, please clarify who assessed safety and adverse events.

**We have described in Methods under the Ethics Statement that we used a DSMB in this study.**

* Please clarify recruitment method.

**We have described recruiting methods: using a combination of electronic advertisements on the University of Utah IRB website as well as study posters placed.**

* Please move the CONSORT diagram to Figure 1.

**Corrected.**

* Please include a completed CONSORT 2025 checklist and ensure that the diagram adheres to the CONSORT 2025 statement.

**We have completed a CONSORT 2025 checklist which adheres to the CONSORT 2025 statement.**

* In Figures, where data points are discrete, please ensure that they are depicted in the figures as discrete data and not as a continuous line.

**We have submitted revised figures according to these guidelines (and utilizing 95% CIs).**

* Please provide the unadjusted comparisons as well as the adjusted comparisons in all relevant Tables.

**We have also included tables S1b, S2b, S3b, S4b in the Supplementary Appendix which show mixed model analyses adjusting for gender and group cohort (as fixed effects). Also see S5 table which has been revised to include adjusted p-values as well reflecting inclusion of gender and group cohort as fixed effects. Of note, we had previously described in the manuscript under Primary Outcome Results that adjusted comparisons did not change significance of effect. We have elaborated in the Secondary Outcome Results that adjusted comparisons retained all significant effects in secondary outcomes with the exception of the MBI(EE) subscale which we had not previously included.**

* Please specify the variables controlled for in all relevant Tables

**We have specified variables controlled for in all relevant tables, see Tables S1b, S2b, S3b, S4b in the Supplementary Appendix which show mixed model analyses adjusting for gender and group cohort (as fixed effects). Also see S5 table which has been revised to include adjusted p-values as well reflecting inclusion of gender and group cohort as fixed effects. Also see Tables S12-S16. We have also clarified in this in our manuscript Results section to elaborate in relation to Secondary Outcomes which we had not done previously.**

* Please discuss the potential for selection/recruitment bias of participants.

**We have included this sentence in the Discussion: “nonetheless, selection bias may have played a role in the participants enrolling in the study.”**

* Please also caveat claims of safety--i.e. discuss the role of oversight/supervision of psilocybin use in treatment settings.

**Have included this sentence in the discussion of safety outcomes in the discussion: “however it is important to note the role of oversight, supervision, and screening procedures in this regard.”**


For RCTs:

* Please complete the CONSORT 2025 checklist and ensure that all components of CONSORT 2025 are present in the manuscript, including how randomization was performed, allocation concealment, blinding of intervention, definition of lost to follow-up, power statement. When completing the checklist, please use section and paragraph numbers, rather than page numbers. The checklist should be included as supporting information, and should be cited in the article.

**We have completed the CONSORT 2025 checklist according to these guidelines. I have cited line numbers for reference.**

* As your trial had to undergo important modifications in response to extenuating circumstances, please complete the CONSERVE-CONSORT checklist and provide in your Supporting Information.

**We have included a CONSERVE-CONSORT checklist describing extenuating circumstances related to the COVID-19 pandemic as well as change in enrollment from 24-25 participants. This change in enrollment was made given an early drop out in the MBSR+PAP arm and desire to have 12 participants in that intervention arm.**

* PLOS Medicine requires that all trials be prospectively registered in one of registries recognized by WHO. Please provide information on study registration in the Methods section.

**Have included www.clinicaltrials.gov registration information.**

* Your trial was registered after the participants were randomized. Please explain in the paper why your trial was registered late. In your rebuttal letter, please indicate if you are conducting or have conducted any related or similar trials, and confirm that those have been registered.

**To clarify, the clinicaltrials.gov registration for this trial (NCT05557643)** **was slightly delayed due to administrative issues but was registered as of September 26, 2022 prior to the consenting and randomization of our first participant (January 2023). You may be confused by the fact that we conducted a similar trial with group psilocybin assisted therapy (NCT04522804,** [**https://clinicaltrials.gov/study/NCT04522804?term=HOPE%20psilocybin&rank=1**](https://clinicaltrials.gov/study/NCT04522804?term=HOPE%20psilocybin&rank=1)**) which was also preregistered.**

* Some of the outcome measures or methods appear to differ between the submitted manuscript and the trial registry and/or protocol. Please clarify and explain the discrepancy. If the outcomes were not prespecified in the protocol, please indicate that they were post hoc and explain why they were added. Post hoc comparisons should be presented as hypothesis generating rather than conclusive.

**The clinicaltrials.gov registration specifies 24 participants and we enrolled 25 total after submitting an IRB amendment so as to complete 12 total participants in the MBSR+PAP arm (due to one dropout). The McGill Quality of Life Inventory was not administered due to transcription errors with REDCap design.**

**Mindfulness related measures (state and trait mindfulness) described in the registration and the protocol are not addressed in this manuscript as they will be included in a subsequent manuscript looking specifically at mindfulness outcomes. Similarly, we obtained video-based behavioral data using the Storyline Health smart phone application and that data is not presented here as it is still being analyzed and will be included in a future publication focused on mechanistic outcomes.**

**Results for the PTSD Checklist for DSM-5 (PCL-5) were initially not presented here because we intended to explore them in a future manuscript. We have now included these outcomes here which have resulted in a significant change to the FDR-corrected secondary outcome p-values (notably the WCS(GC))- see description below. We have adjusted the manuscript accordingly, as well as all relevant tables so as to include these results. Notably this is relevant for Reviewer #2’s comments below regarding multiple comparison corrections for secondary outcome measures.**

**All additional measures will be made available upon request and/or in subsequent publications to ensure transparency and completeness of reporting. We have added language to the manuscript to clarify why these exploratory measures have been left out of this analysis given intention for future publications.**

* In accordance with ICMJE requirements, PLOS Medicine requires prospective, public registration of a data sharing plan (as part of mandatory clinical trials registration) for all clinical trials that began enrollment on or after January 1, 2019.

**(See below on data sharing)**

* The trial registration or protocol lists secondary outcomes can you please present those results as part of this manuscript, or indicate why that is not possible? If this is not possible, can you please indicate when you plan to publish those results?

**We initially did not present data from the PCL-5 (listed as a secondary measure in the protocol) but upon reflection have now included this in our manuscript and Supplementary Appendix. The initial intention was to present this data in a subsequent manuscript given that while there were no significant results with this measure at 2 weeks or 6 months there were interesting post-hoc analyses that seemed worth exploring but for which we did not have space here to address. In virtue of including the PCL-5 outcomes in this analysis this has changed our FDR-adjusted p-value calculations for the secondary outcome measures. Notably the WCS(GC) 2 week adjusted p-value is now changed from 0.045 to 0.055 (losing significance with alpha = 0.05). This is the only notable change and we have revised our manuscript and all relevant tables to account for this. Notably, this is relevant for Reviewer #2’s comments here related to multiple comparison corrections on secondary measures. Of note including PCL-5 outcomes has changed Table 2, S2 table, S5 table, S8-11 Tables.**

**We recognize that incorporating these additional outcomes alters the multiple comparison adjustments and slightly shifts the interpretation of one of our secondary outcomes- the WCS(GC) 2 week outcomes. We believe, however, that inclusion of all prespecified secondary outcomes strengthens the report, provides readers with a complete view of the trial results, adheres to CONSORT reporting guidelines, and avoids any appearance of selective reporting. The primary study results related to primary outcome measures remain unchanged. More detailed exploratory analyses of PCL-5 trajectories and mechanisms will still be pursued in a future manuscript, but the core findings are now summarized here for completeness**.

**We have other exploratory outcomes that were not possible to include in this manuscript for space reasons as we wanted to focus this manuscript on our primary and main outcomes from the trial. These additional outcomes not included here include mindfulness related outcomes (state and trait) as well as behavioral phenotyping outcomes we obtained using a smartphone based AI tool called Storyline Health. We are currently working on subsequent manuscripts which we intend to submit following publication of this primary outcome manuscript.**

* The sample size listed in the submitted manuscript and the trial registry differ. Please explain the discrepancy.

**We submitted an amendment to the IRB to allow us to enroll a total of 25 participants so as to be able to complete 12 participants in the MBSR + PAP arm total and preserve group size in the context of a study drop out. We have mentioned this in the manuscript.**

* The main analysis should be intention to treat (ie, all individuals randomized are included in the analysis in the groups to which they were originally assigned. If the study included dropouts, specify whether their data are imputed and if so using what method. Please refer to as modified ITT).

**To clarify, we included all individuals randomized in the analysis in the groups to which they were originally assigned, thus our analysis was an ITT analysis. While ITT includes *all* randomized participants regardless of protocol adherence, a modified ITT selectively excludes certain individuals after randomization—typically those who:**

- **Never received the intervention**
- **Lack baseline or post-baseline data**
- **Were found ineligible after randomization**
- **Withdrew before any meaningful participation**

**We did not exclude individuals for any of these reasons. As such, it is accurate to characterize our study as following an ITT approach.**

**Also, to clarify, we did not use any imputations. The ITT analysis was performed using mixed-effects models, which use all available data without dropping participants. No last-observation-carried-forward or multiple imputation was applied.**

* The CONSORT flowchart should be figure 1, please revise.

**Have corrected.**

* In the flow diagram, please indicate the number of individuals in each group analyzed in the ITT analysis.

**Done, included in CONSORT flowchart.**

* Please present the safety data for the study including numbers of specific events and whether or not adverse events are thought to be related to treatment.

**Included in table of Adverse Events.**

* In this test of superiority it is not possible to determine that the intervention and control are equivalent, just that the intervention is not superior to the control, please update the manuscript accordingly.

**We have adjusted language in the manuscript to this effect.**

* Causal language - In trials, there is usually a distinction in the language in terms of causal vs associational for primary and secondary trial outcomes. It would be beneficial to use associational language in the discussion and other sections for secondary outcomes.

**We have made relevant changes in this regard to employ associational language in both the Results section and Discussion section.**

* Please report your abstract according to CONSORT for abstracts, following the PLOS Medicine abstract structure (Background, Methods and Findings, Conclusions) <https://www.equator-network.org/reporting-guidelines/consort-abstracts/>

**Have revised the abstract accordingly.**

* Please include the clinical trial registry number in the abstract.

**Have included.**

Per CONSORT, please note that only the primary outcome of the trial should be reported in your Abstract. Secondary outcomes should only be included in the Abstract if all secondary outcomes are fully reported. For trials that have many secondary outcomes, the Abstract should be limited to reporting the primary outcome.

**Have revised the abstract accordingly.**

GENERAL
* Please review your text for claims of novelty or primacy (e.g. 'for the first time') and remove this language. In addition, please check that any use of statistical terms (such as trend or significant) are supported by the data, and if not please remove them.

**Have corrected.**

* Statistical reporting: Please revise throughout the manuscript, including tables and figures.
- Please report statistical information as follows to improve clarity for the reader "22% (95% CI [13,28]; p</=)".

**Have corrected.**

- Please separate upper and lower bounds with commas instead of hyphens as the latter can be confused with reporting of negative values.

**Have corrected.**

- Please repeat statistical definitions (HR, CI etc.) for each set of parentheses.

**Have corrected.**

* Please verify PLOS style of referencing (references before, not after periods).

**We have corrected all referencing.**

* Please avoid claims of causality in the Discussion related to observations at 6 months. Please refer to observed associations, specifically in the paragraph staring line 474.

**Thanks for this suggestion. We have altered this language to emphasize association, rather than implied causality and I removed the phrase “suggesting that psilocybin may accelerate the therapeutic effects of mindfulness.”**

* The Data Availability Statement (DAS) requires revision. For each data source used in your study: 

a) If the data are freely or publicly available, note this and state the location of the data: within the paper, in Supporting Information files, or in a public repository (include the DOI or accession number).

b) If the data are owned by a third party but freely available upon request, please note this and state the owner of the data set and contact information for data requests (web or email address). Note that a study author cannot be the contact person for the data.

c) If the data are not freely available, please describe briefly the ethical, legal, or contractual restriction that prevents you from sharing it. Please also include an appropriate contact (web or email address) for inquiries (again, this cannot be a study author).
*Please ensure that there are no secondary identifiers in the participant information.

**This study enrolled a small, narrowly defined professional population (frontline healthcare workers) with unique ethical vulnerabilities in terms of identification, involved an intervention with a Schedule 1 compound with heightened ethical concerns around confidentiality and disclosure, and also includes sensitive mental-health information that could theoretically have impact on professional standing and employment. As such there is a material risk of re-identification even after standard HIPAA de-identification. Our informed consent process and IRB approval did not permit unrestricted public posting of row-level data. Consistent with journal policies allowing controlled access when legal/ethical restrictions apply, we will share a de-identified, analysis-ready dataset with qualified researchers with a data sharing agreement upon request, in conjunction with the University of Utah Institutional Review Board.**

**Access will be granted to qualified researchers for non-commercial scientific purposes subject to a Data Use Agreement, documentation of human-subjects/IRB approval or exemption, and approval by the chair of our Data Safety and Monitoring Committee (DSMB). Due to ethical and legal constraints related to participant privacy and re-identification risk, public posting of the full row-level dataset is not permitted. We feel that a controlled-access model best balances participant privacy with reproducibility.**

**The chair of our DSMB (Brian Mickey MD, PhD- brian.mickey@hsc.utah.edu) will be the point of contact for data sharing inquiries.**

FUNDING STATEMENT
* The funding statement should include: specific grant numbers, initials of authors who received each award, URLs to sponsors’ websites. Grant numbers are missing and there is no mention of NIH grants. Also, please state whether any sponsors or funders (other than the named authors) played any role in study design, data collection and analysis, the decision to publish, or preparation of the manuscript. If they had no role in the research, include this sentence: “The funders had no role in study design, data collection and analysis, decision to publish, or preparation of the manuscript.”

COMPETING INTERESTS STATEMENT
* All authors must declare their relevant competing interests per the PLOS policy, which can be seen here: <https://journals.plos.org/plosmedicine/s/competing-interests> For authors with ties to industry, please indicate whether any of the interests has a financial stake in the results of the current study.
For other authors that have no competing interests, please so state.

**We have completed the competing interests statement and funding statement which is at the end of the manuscript after the references and legend of supporting information.**


Comments from Reviewers:

Reviewer #1: All my comments have been addressed
**Thank you for your thoughtful attention to our manuscript and your suggested revisions.**


Reviewer #2: Major Concerns

1. The primary finding that psilocybin benefits were significant at 2 weeks but waned by 6 months deserves more thorough discussion. This raises important questions about durability of effects and optimal follow-up interventions. The authors should consider whether this pattern suggests need for booster sessions or different integration protocols.

**Thanks for drawing our attention to the need to better articulate this waning effect. We have elaborated with the following: “However, this between-groups difference waned at the 6-month endpoint such that participants in both study arms were reporting lower levels of depression, raising questions as to durability of effect and the possible need for booster sessions or more extended integration periods.”**

2. The MBSR+PAP arm received substantially more therapeutic contact (40 vs 28 hours). While the authors conducted sensitivity analyses, this remains a significant limitation that could account for observed differences. Future studies should match contact time between arms.

**Thanks for emphasizing this point. We have addressed this with the following language in the discussion:**  **“The interventions differed between arms, with the PAP group participating in a psilocybin dosing day, while the MBSR-only group attended a silent meditation retreat. This design ensured that both groups received a form of intensive experience, although the nature of these experiences were not equivalent and the PAP+MBSR arm received a larger amount of total therapeutic contact time given the additional preparatory and integration sessions. This difference in therapeutic contact time may have a confounding effect on outcomes. Future studies should equate therapeutic contact time between study arms.”**

3. With only 25 participants, 96% white, and 72% female, generalizability is severely limited. The authors appropriately acknowledge this but should discuss implications for different populations more thoroughly.

**Thank you for stressing this point. We have elaborated further in the following way: “The homogeneity of our sample, consisting predominantly of white female participants, further restricts the generalizability of our findings to more diverse populations: it remains an open question whether these effects would be extended to minority population healthcare workers who can face additional workplace stressors.”**

Minor Comments
1. The multiple comparisons corrections are appropriate, and it's notable that only the connectedness measure retained significance after FDR correction for secondary outcomes.

**Thank you for this feedback. Of note we have now included outcomes for the PTSD Checklist for DSM-5 (PCL-5). This questionnaire was included in the protocol as secondary outcome but was not included in the initial draft given intention to explore these effects in a separate manuscript given that while the overall analysis did not show significant between group differences on this measure there were post-hoc analyses performed that were of interest and we were unable to explore these sufficiently in this manuscript, particularly in relation to post traumatic stress symptoms in this study population. We have now decided to include this outcome measure here for consistency and transparency of our outcome reporting as well as consistency with CONSORT reporting standards. This has affected the FDR corrections for secondary outcome measures such that the effect for WCC (GC) is now insignificant (changed from 0.045 to 0.055). We have made relevant changes in this regard to the manuscript as well as included tables as has been described above to ensure that these corrected p-values are clear. We recognize that incorporating these additional outcomes alters the multiple comparison adjustments and slightly shifts the interpretation of one of our secondary outcomes (WCS(GC)). We believe, however, that inclusion of all prespecified secondary outcomes strengthens the report, provides readers with a complete view of the trial results, and avoids any appearance of selective reporting. More detailed exploratory analyses of PCL-5 trajectories and mechanisms will still be pursued in a future manuscript, but the core findings are now summarized here for completeness**.

2. The finding that expectancy was associated with outcomes in the MBSR-only arm but not the psilocybin arm is intriguing and warrants further discussion about mechanisms of action.

**Yes, this is an interesting observation and it is not entirely clear what to make of this relationship between expectancy and outcomes in the MBSR-only arm. There were no clear relationships between expectancy for MBSR and either adherence to MBSR or attendance at MBSR sessions (which might be one predicted relationship that could partially account for this finding). We have added the following sentence to the discussion to elaborate on this: “It is also possible that expectancy for MBSR-only predicted level of engagement with mindfulness practice not captured by our measurements and analysis here.”**

Reviewer #3: Excellent revision and responsiveness to all reviewers. No further comments
**Thank you for your thoughtful attention and contributions to our manuscript.**

-----------------------------------------------------------------------------------------------------------------------

*Additional Technical Requests per email from Sara Gheit 8/27/25:*

Ref: Psilocybin-Assisted Group Psychotherapy + Mindfulness Based Stress Reduction (MBSR) for Frontline Healthcare Provider COVID-19 Related Depression and Burnout: A Randomized Clinical Trial
PMEDICINE-D-24-04423R2

Dear Dr. Lewis ,

Thank you for your patience while we performed technical checks on your submission. Please see below for a list of requirements for you to address while conducting your revisions. 

Journal Requirements:

1) We have noticed that you have uploaded Supporting Information files, but you have not included a complete list of legends. Please add a full list of legends for your Supporting Information files (CONSORT Checklist.doc and Protocol and Statistical Analysis Plan- revised.docx   )after the references list.

**Thank you, we have completed a full list of legends following the references list.**

2) Please ensure that your Competing Interest Statement is updated in the online submission form beginning with the statement "I have read the journal's policy and the authors of this manuscript have the following competing interests:" as provided in PLOS Medicine Competing Interests Statement.docx.

**Thank you, I will ensure this Competing Interests Statement is correctly updated.**
